# Anti-Proliferative Effects of Iridoids from *Valeriana fauriei* on Cancer Stem Cells

**DOI:** 10.3390/ijms232214206

**Published:** 2022-11-17

**Authors:** Hayato Yoshikawa, Takahiro Matsumoto, Takahiro Kitagawa, Masaya Okayama, Tomoe Ohta, Tatsusada Yoshida, Tetsushi Watanabe

**Affiliations:** 1 Department of Public Health, Kyoto Pharmaceutical University, 1 Misasagi-Shichono-cho, Yamashina-ku, Kyoto 607-8412, Japan; 2 Faculty of Pharmaceutical Sciences, Nagasaki International University, Nagasaki 859-3298, Japan

**Keywords:** *Valeriana fauriei*, valerianairidoid, ECD, cancer stem cell, cancer treatment, cancer prevention

## Abstract

We isolated seven new iridoid glucosides (valerianairidoids I–VII; **1**–**3**, **6**, **7**, **9**, and **12**) and six known compounds from the methanol extract of the dried rhizomes and roots of *Valeriana fauriei*. Chemical and spectroscopic data were used to elucidate the chemical structures of the seven new iridoid glucosides, and their absolute configurations were determined by comparing their electronic circular dichroism (ECD) spectra with those determined experimentally. Aglycones **1a**, **6a**, and **9a**, which were obtained by enzymatic hydrolysis of the isolated iridoid glucosides, exhibited anti-proliferative activities against cancer stem cells (CSCs) established by a sphere-formation assay using human breast cancer (MDA-MB-231) and human astrocytoma (U-251MG) cells. Interestingly, these iridoids selectively showed anti-proliferative activities against CSCs from MDA-MB-231 cells. These results suggest that the iridoids obtained in this study may have potency as a breast cancer treatment and as preventive agent via exterminating CSCs.

## 1. Introduction

*Valeriana fauriei* Briquet (Valerianaceae) is abundant in both Japan and China and has been used for centuries as a sedative and antispasmodic agent [1]. Iridoids bearing isovaleryl moieties [2] and cyclized guaiane-type sesquiterpenes [3], among others, have been isolated as *V. fauriei* constituents. As part of our ongoing research aimed at discovering new cancer-treatment and preventive agents [4,5,6,7], we found that cyclized guaiane-type sesquiterpenes and lignans from *V. fauriei* show cell-death-inducing activities against adriamycin-treated (ADR-treated) HeLa cells by inhibiting heat-shock protein (HSP), and through anti-proliferative effects against cancer stem cells (CSCs) and human astrocytoma cells [8]. As part of our continuing study, we isolated iridoid glycosides and evaluated their anti-proliferative activities against CSCs.

CSCs have been identified in many types of malignancy—including leukemia and breast, colorectal, and brain cancers [9]—which are leading causes of cancer recurrence following anti-cancer drug treatment because these cells are resistant to current anti-cancer drugs and radiation therapy, and play important roles in metastasis by acquiring mesenchymal properties, including improved motility and enhanced invasiveness [10]. Therefore, compounds that are anti-proliferative against CSCs are potentially useful in cancer treatment and as preventive agents. Pyranocoumarin [11], lignans [12], and sesquiterpenes [13] have been reported as naturally occurring agents that are anti-proliferative toward CSCs. In this report, we describe the isolation, structure determination, and anti-proliferative activities of isolated iridoid glycosides and their derivatives against CSCs obtained using a sphere-formation assay with MDA-MB-231 and U-251MG cells.

## 2. Results and Discussion

### 2.1. Isolating Constituents

**Figure 1:**
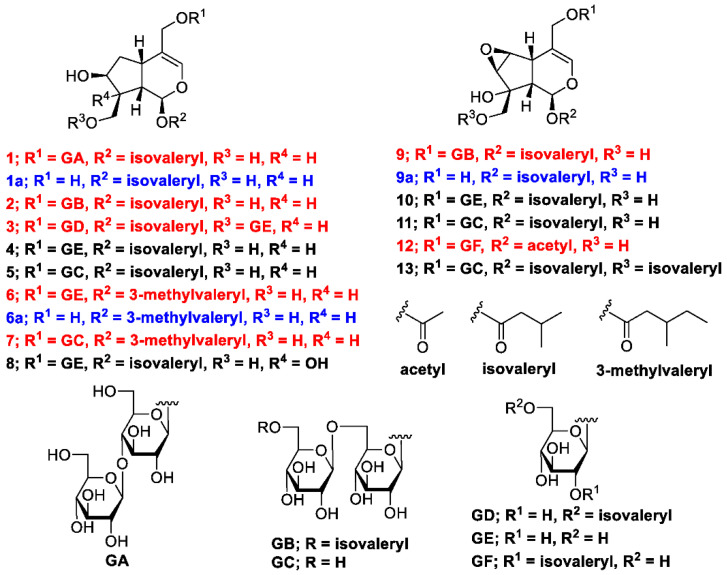
Chemical structures of constituents **1**–**13** isolated from *V. fauriei* and their derivatives **1a**, **6a**, and **9a**.

The methanol (MeOH) extract of the dried rhizomes and roots of *V. fauriei* was partitioned in ethyl acetate–H_2_O (1:1, *v*/*v*) to furnish an ethyl acetate fraction and an aqueous layer. The latter was partitioned with *n*-BuOH to yield a BuOH fraction (2.4%), which was subsequently separated by normal- and reverse-phase silica-gel column chromatography and high-performance liquid chromatography (HPLC) to yield seven new compounds with the yields (%, weight/weight from the weight of the isolated compounds and dried plant) described later: valerianairidoid I (**1**, 0.0017%), valerianairidoid II (**2**, 0.00066%), valerianairidoid III (**3**, 0.0025%), valerianairidoid IV (**6**, 0.0012%), valerianairidoid V (**7**, 0.0027%), valerianairidoid VI (**9**, 0.00076%), and valerianairidoid VII (**12**, 0.00011%), together with six known compounds, namely patrinoside (**4**, 0.012%) [14], kanokoside D (**5**, 0.0022%) [14], suspensolide F (**8**, 0.0027%) [15], kanokoside A (**10**, 0.0020%) [14], kanokoside C (**11**, 0.0036%) [14], and 10-isovaleryloxykanokoside C (**13**, 0.0043%) [2] (Figure 1).

### 2.2. Determining the Structures of Valerianairidoids I–VII (***1***–***3***, ***6***, ***7***, ***9***, and ***12***)

**Figure 2:**
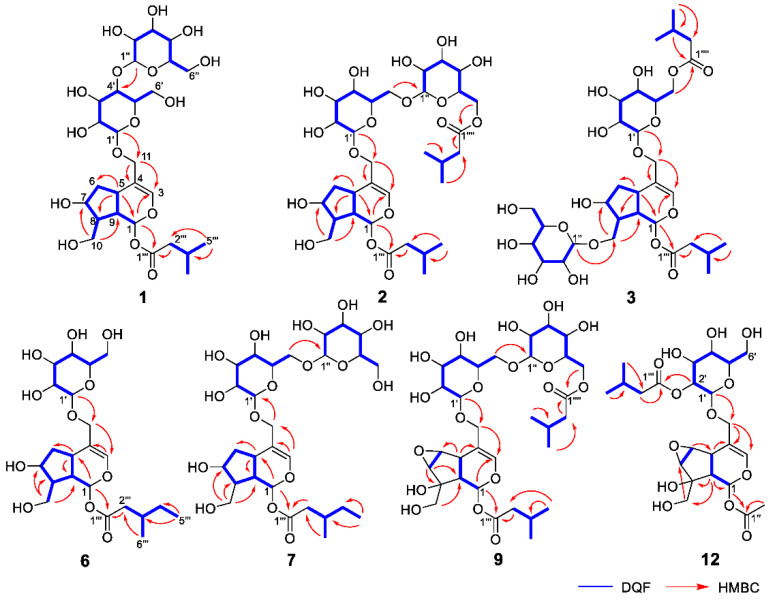
Important 2D NMR correlations observed for new compounds **1**–**3**, **6**, **7**, **9**, and **12**.

**Figure 3:**
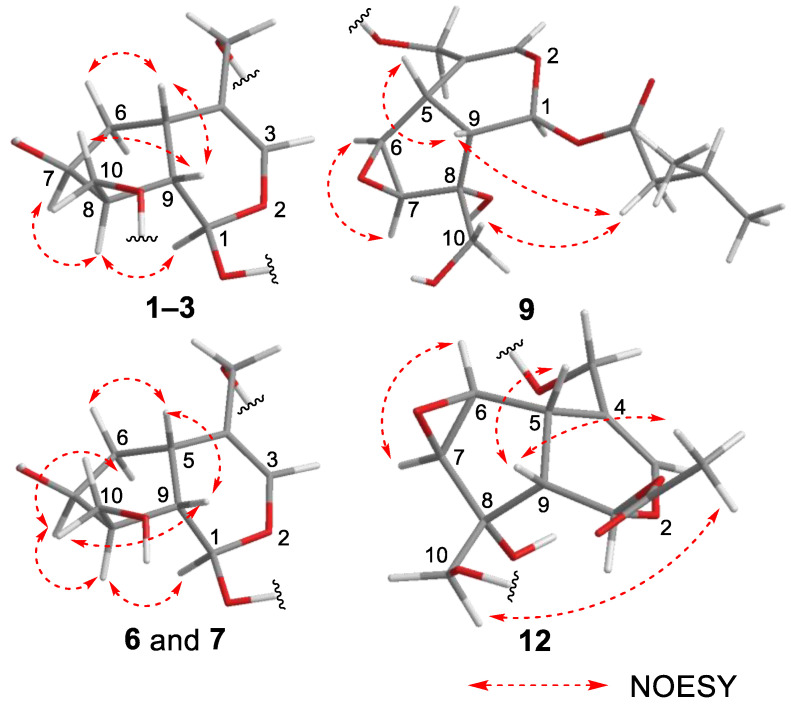
Important NOESY correlations observed for new compounds **1**–**3**, **6**, **7**, **9**, and **12**.

**Figure 4:**
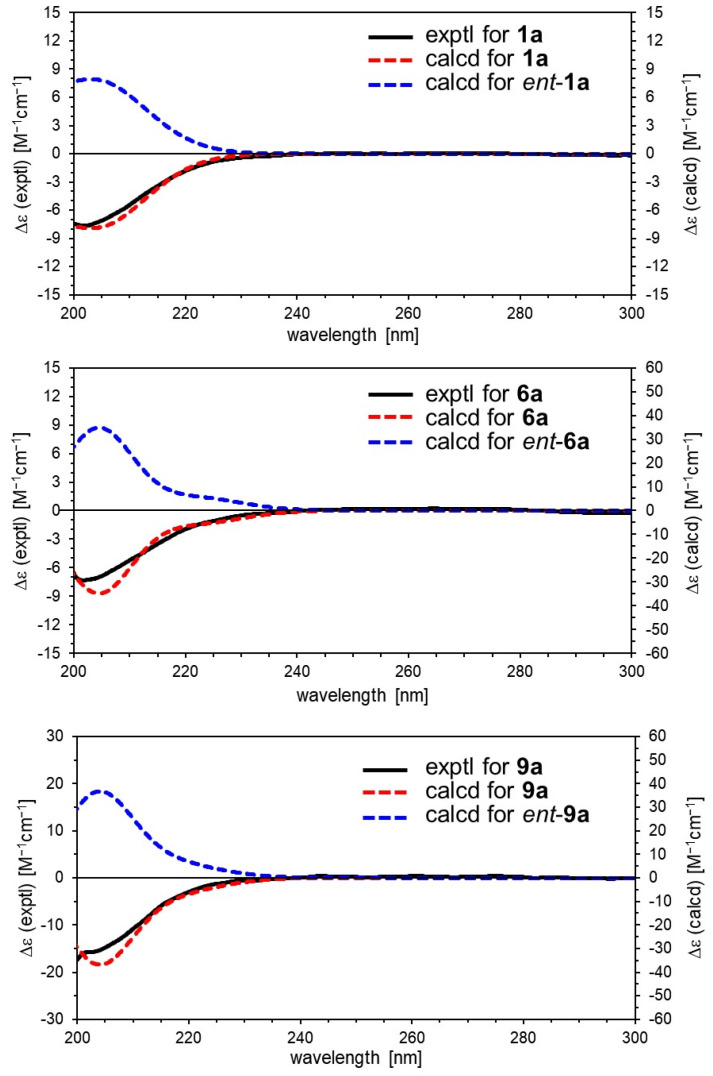
Experimental and calculated ECD spectra for **1a**, **6a**, and **9a**.

Valerianairidoid I (**1**) was isolated as an amorphous solid with a negative optical rotation ([*α*]^25^_D_ −36.3 in MeOH). Its molecular formula (C_27_H_44_O_16_) was determined by high-resolution mass spectrometry (HRMS) and ^13^C nuclear magnetic resonance (NMR) spectroscopy (*m/z* 647 [M+Na]^+^). The ^1^H and ^13^C NMR (CD_3_OD) spectra of **1** (Table 1) show signals consistent with an iridoid moiety {a methylene [*δ* _H_ 1.82 (m, H-6 *α*) and 2.06 (m, H-6 *β*)], two methylenes, each bearing an oxygen functional group [*δ* _H_ 3.73 (dd, *J* = 5.5, 11.0, H-10a), 3.80 (m, H-10b), 4.08 (d, *J* = 11.0, H-11a), and 4.23 (d, *J* = 11.0, H-11b)], a methine bearing an oxygen function group [*δ* _H_ 4.33 (m, H-7)], three methines [*δ* _H_ 3.01 (dd, *J* = 7.6, 15.8, H-5), 1.94 (m, H-8), and 2.17 (m, H-9)], an olefinic group [*δ*_C_ 140.1 (C-3) and 116.4 (C-4)], an acetal moiety [*δ* _C_ 93.6 (C-1)]}, an isovaleryl moiety {two methyl groups [*δ* _H_ 0.96 (d, *J* = 6.9, H-4‴ and 5‴)], a methylene [*δ* _H_ 2.23 (d, *J* = 6.9, H-2‴)], a methine [*δ* _H_ 2.09 (m, H-3‴)], and an ester group [*δ* _C_ 173.3 (C-1‴)]}, and a cellobiose moiety. The presence and position of the abovementioned cellobiose moiety were determined using double-quantum-filtered homonuclear correlation spectroscopy (DQF COSY) and heteronuclear multiple bond coherence (HMBC) spectroscopy (Figure 2). Long-range H-1′/C-11, H-1″/C-4′, and H-1/C-1‴ correlations were observed, suggesting that the cellobiose moiety was attached at C-11 and the isovaleryl moiety was attached at C-1. The relative configuration was determined using nuclear Overhauser enhancement spectroscopy (NOESY) (Figure 3), which revealed H-1/H-8, H-6*α*/H-7, and H-7/H-8 NOESY cross-peaks that indicate that H-1, H-6*α*, H-7, and H-8 are located on the same side in **1**. In addition, H-5/H-9, H-5/H-6*β*, and H-9/H-10 NOESY cross-peaks suggest that H-5, H-6*β*, H-9, and H-10 are located on the same side. We obtained aglycone **1a** by enzymatically hydrolyzing **1**. While **1a** was identified to be a patrinoside-aglucone by NMR spectroscopy and MS, its absolute configuration was not previously discussed in [16]. Therefore, we determined the absolute configuration of **1a** by calculating its electronic circular dichroism (ECD) spectrum. The calculated ECD data for 1*S*,5*S*,7*S*,8*S*,9*S*-configured **1a** are in good agreement with the experimental data, whereas the calculated ECD spectrum of *ent*-**1a** (1*R*,5*R*,7*R*,8*R*,9*R*) is essentially the mirror image of that acquired experimentally (Figure 4). Finally, **1** was subjected to acid hydrolysis using 20% aqueous H_2_SO_4_ in 1,4-dioxane, which yielded d-glucose by HPLC separation of its diastereomeric tolylthiocarbamoyl thiazolidine derivative [17]. The coupling constants (*J* = 7.6 Hz) for the anomeric position of the two glucoses suggested that they have *β*-configurations at the glycosidic bonds. We conclude that the chemical structure of valerianairidoid I (**1**) is as shown in Figure 1, based on the evidence provided above.

Valerianairidoids II and III (**2** and **3**) were isolated as amorphous powders with negative optical rotations (**2**: [*α*]^25^_D_ −31.3; **3**: [*α*]^25^_D_ −20.5 in MeOH). Their molecular formulas (C_32_H_52_O_17_) were determined using HRMS and ^13^C NMR spectroscopy. A comparison of the NMR data for **1**, **2**, and **3** reveals that they contain the same aglycone, a glucose moiety attached at C-11, and an isovaleryl moiety attached at C-1. Moreover, **2** and **3** each contain an additional glucose unit and an additional isovaleryl substituent. The HMBC correlations [**2**: H-6″/C-1′′′′ and H-6′/C-1″] and [**3**: H-1″/C-10 and H-6′/C-1′′′′] and total correlation spectroscopy (TOCSY) correlation [**3**: H-1′/H-6′] suggest that the additional glucose moiety is attached at C-6′ in **2** and C-10 in **3**, and that the additional isovaleryl moiety is attached at C-6″ in **2** and C-6′ in **3** (Figure 2). Enzymatic hydrolysis of **2** gave **1a**, which suggests that **1** and **2** share the same absolute configuration, and the absolute configuration of **3** was deduced to be identical to that of **1** and **2**. Based on the above data, we conclude that valerianairidoids II and III (**2** and **3**, respectively) have the chemical structures shown in Figure 1.

Valerianairidoids IV and V (**6** and **7**) were isolated as amorphous solids with negative optical rotations (**6**: [*α*]^25^_D_ −21.6; **7**: [*α*]^25^_D_ −48.9 in MeOH). Their molecular formulas (**6**: C_22_H_36_O_11_ and **7**: C_28_H_46_O_16_) were determined by HRMS, high-resolution electrospray ionization MS (HRESIMS), and ^13^C NMR spectroscopy. The ^1^H and ^13^C NMR (CD_3_OD) data suggest that valerianairidoid IV (**6**) contains an iridoid moiety and a glucose moiety at C-11, in a similar manner to **1**. In addition, **6** contains a 3-methylvaleryl moiety {two methyl groups [*δ* _H_ 0.89 (t, *J* = 7.8, H-5‴) and 0.92 (d, *J* = 6.8, H-6‴)], two methylene groups [*δ*_H_ 2.12 (m, H-2‴a), 2.33 (dd, *J* = 6.0, 15.0, H-2‴b), 1.24 (m, H-4‴a) and 1.37 (m, H-4‴b)], a methine [*δ*_H_ 1.84 (m, H-3‴)], and an ester moiety [*δ*_C_ 173.5 (C-1‴)]}. The positions of the 3-methylvaleryl and glucose moieties in **6** were determined from the DQF COSY and HMBC data shown in Figure 2. A comparison of the NMR data for **6** and **7** reveals that **7** contains one more glucose moiety attached at C-6′ compared to **6**. The same aglycone (**6a**) was obtained when **6** and **7** were enzymatically hydrolyzed using *β*-glucosidase. The planar chemical structure and relative configuration of **6a** were elucidated from the NMR and MS data (Table 2). The absolute configuration of **6a** was determined to be 1*S*,5*S*,7*S*,8*S,*9*S* based on the experimental and calculated ECD spectra, in a similar manner to **1a** (Figure 4). We conclude that the chemical structures of valerianairidoids IV and V (**6** and **7**, respectively) are as shown in Figure 1.

Valerianairidoid VI (**9**) was isolated as an amorphous solid with a negative optical rotation ([α]^25^_D_ −15.4 in MeOH), and its molecular formula (C_32_H_50_O_18_) was determined by HRESIMS and ^13^C NMR spectroscopy. The ^1^H and ^13^C NMR (CD_3_OD) spectra suggest that **9** contains an iridoid moiety, a gentiobiose moiety, and two isovaleryl moieties (Table 3); their positions were determined by DQF COSY and HMBC NMR spectroscopy (Figure 2), and the relative stereochemistry of **9** was determined by NOESY spectroscopy (Figure 3). Aglycone **9a** was obtained by the enzymatic hydrolysis of **9** using cellulase and identified as the aglycone of kanokoside A [18]; however, its absolute configuration was not determined previously. The absolute configuration of **9** was further established as 1*S*,5*S*,6*S*,7*S*,8*R*,9*S* by comparing the experimental and calculated ECD spectra of **9a**. We conclude that the chemical structure of valerianairidoid VI (**9**) is as shown in Figure 1.

Valerianairidoid VII (**12**) was isolated as an amorphous powder with a negative optical rotation ([α]^25^_D_ −47.8 in MeOH); its molecular formula (C_23_H_34_O_13_) was determined by HRMS and ^13^C NMR spectroscopy, and its ^1^H and ^13^C NMR (CD_3_OD) spectra suggest that **12** contains iridoid and glucose moieties in a similar manner to **9**. In addition, **12** contains isovaleryl and acetyl moieties {a methyl [*δ* _H_ 2.05 (s, H-2″) and an ester [*δ* _C_ 172.4 (C-1″)]} (Table 3). The positions of the isovaleryl and acetyl moieties were determined by DQF COSY and HMBC NMR spectroscopy (Figure 2). The absolute configuration of the iridoid moiety was determined to be that same as in **9**. We conclude that the chemical structure of valerianairidoid VII (**12**) is as shown in Figure 1.

### 2.3. Evaluating Anti-Proliferative Activities against Non-CSCs and CSCs

The antiproliferative activities of isolated compounds **1**–**13** and aglycones **1a**, **6a**, and **9a** against MDA-MB-231 and U-251MG cells (non-CSCs) and their CSCs were evaluated. The CSCs used in this study were established using a sphere-formation assay, with sphere formation confirmed by cell morphology and the expression of the Nanog stem-cell marker in both MDA-MB-231 and U-251MG cells [13]. ADR was used as the positive control, and cell viability was evaluated using the CellTiter-Glo^®^ 3D cell viability assay. None of the isolated compounds showed any anti-proliferative activity against either the non-CSC or CSC cell line [**1**–**13**; IC_50_ > 100 μM]. On the other hand, aglycones **1a**, **6a**, and **9a** showed significant anti-proliferative activities against CSCs from MDA-MB-231 [IC_50_; **1a** (45.0 ± 5.3 μM), **6a** (17.6 ± 1.0 μM), and **9a** (47.7 ± 3.9 μM)]. Interestingly, **1a**, **6a**, and **9a** showed anti-proliferative activities against CSCs with lower IC_50_ values than non-CSCs of MDA-MB-231 [IC_50_; **1a** (>100 μM), **6a** (47.7 ± 4.2 μM), and **9a** (>100 μM)] cells (full experimental data applied for IC_50_ calculations are described in Supplementary Materials S5). On the other hand, **1a**, **6a**, and **9a** showed no activity (IC_50_ > 100 μM) against either non-CSCs or CSCs from U-251MG cells. Therefore, these aglycones may have an inhibitory effect on the characteristic signal pathway(s) in CSCs from MDA-MB-231 cells.

## 3. Materials and Methods

### 3.1. General Experimental Procedures

Specific rotations were measured using a P-2200 digital polarimeter (l = 5 cm; JASCO, Tokyo, Japan). IR spectra were measured using a JASCO FT/IR-4600 typeA spectrometer; ECD spectroscopy was performed using a JASCO J-1500 spectrometer. ESI and high-resolution ESI mass spectra were recorded on a Shimadzu LCMS-IT-TOF instrument. FAB and HRFABMS data were recorded using a JASCO SX-102A mass spectrometer. ^1^H NMR spectra were recorded on JEOL ECS400 (400 MHz) and JNM-ECA 600 (600 MHz) spectrometers (JEOL). ^13^C NMR spectroscopy was performed on a JEOL JNM-ECA 600 (150 MHz) spectrometer. 2D-NMR experiments were carried out on a JEOL JNM-ECA 600 (600 MHz) spectrometer (JEOL).

Normal-phase silica-gel column chromatography was performed using silica gel 60 (63–210 μm; Kanto Chemical Co., Tokyo, Japan). Reverse-phase silica-gel column chromatography was performed using C18-OPN gel (140 μm; Nacalai Tesque, Kyoto, Japan). HPLC was performed using an SPD-M10Avp UV-vis detector (Shimadzu, Kyoto, Japan).

### 3.2. Plant Material

The dried rhizomes and roots of *V. fauriei* from Hokkaido (Japan) were purchased from Tochimoto Tenkaido (Osaka Prefecture, Japan) in August 2020.

### 3.3. Extracting and Isolating Compounds

The methanol extract (1150 g) of the dried rhizomes and roots of *V. fauriei* (6 kg) was prepared as previously described [8]. The methanol extract was partitioned into ethyl acetate–H_2_O (1:1, *v*/*v*). The H_2_O layer was mixed at a 1:1 *v/v* ratio with *n*-BuOH to provide a BuOH-soluble fraction (Fr. B, 146.25 g, 2.4%). A portion of the BuOH-soluble fraction (69.56 g) was separated using normal-phase silica-gel column chromatography [CHCl_3_–MeOH (1:0 → 50:1 → 20:1 → 10:1 → 5:1 → 3:1 → 1:1 *v*/*v*)] to yield eight fractions (B1–B8). Fraction B6 (5844.6 mg) was separated by reverse-phase silica-gel column chromatography into ten fractions (B6-1–10). Fraction B6-4 (1830.55 mg) was purified by HPLC {COSMOSIL 5C18-MS-II (250 × 4.6 mm i.d. and 250 × 20 mm i.d.); mobile phase: H_2_O–CH_3_CN (41:9, *v*/*v*)} to yield **4** (354.9 mg). Fraction B6-5 (598.34 mg) was purified by HPLC {COSMOSIL 5C18-MS-II (250 × 4.6 mm i.d. and 250 × 20 mm i.d.); mobile phase: H_2_O–CH_3_CN (41:9, *v*/*v*)} to yield **6** (34.76 mg) and **12** (3.06 mg). Fraction B7 (7048.6 mg) was separated by reverse-phase silica-gel column chromatography into seven fractions (B7-1–7). Fraction B7-3 (2.8577 g) was purified by HPLC {COSMOSIL 5C18-MS-II (250 × 4.6 mm i.d. and 250 × 20 mm i.d.); mobile phase: H_2_O–CH_3_CN (7:3, *v*/*v*)} to yield **8** (76.69 mg) and **10** (57.57 mg). Fraction B7-5 (514.02 mg) was purified by HPLC {COSMOSIL 5C18-MS-II (250 × 4.6 mm i.d. and 250 × 20 mm i.d.); mobile phase: H_2_O–CH_3_CN (7:3, *v*/*v*)} to yield **2** (18.95 mg), **3** (72.15 mg), **9** (21.58 mg), and **13** (123.42 mg). Fraction B8 (17.87 g) was separated by reverse-phase silica-gel column chromatography to yield seven fractions (B8-1–7). Fraction B8-6 (1185.29 mg) was purified by HPLC {YMC-Actus Triart C18 (250 × 4.6 mm i.d. and 250 × 20 mm i.d.); mobile phase: H_2_O–CH_3_CN (3:2, v/v)} to yield **1** (48.23 mg), **5** (61.96 mg), **7** (77.77 mg), and **11** (102.70 mg).

### 3.4. Valerianairidoid I (***1***)

Amorphous solid, [*α*]^25^_D_ −36.3 (*c* 0.3, MeOH), ^1^H NMR (CD_3_OD, 600 MHz) and ^13^C NMR (150 MHz) (Table 1), IR (ATR) ν_max_ 3360, 2931, 1745,1665, 1375, and 1073 cm^−1^, ESI-MS *m/z* 647 [M+Na]^+^, HRESIMS *m/z* 647.2534 [M+Na]^+^ (calcd. for C_27_H_44_O_16_Na, 647.2522).

### 3.5. Valerianairidoid II (***2***)

Amorphous solid, [*α*]^25^_D_ −31.3 (*c* 0.1, MeOH), ^1^H NMR (CD_3_OD, 600 MHz), ^13^C NMR (150 MHz) (Table 1), IR (ATR) ν_max_ 3359, 2957, 1731, 1369, 1294, and 1033 cm^−1^, FABMS *m/z* 731 [M+Na]^+^, HRFABMS *m/z* 731.3092 [M+Na]^+^ (calcd. for C_32_H_52_O_17_Na, 731.3102).

### 3.6. Valerianairidoid III (***3***)

Amorphous solid, [*α*]^25^_D_ −20.5 (*c* 0.5, MeOH), ^1^H NMR (CD_3_OD, 600 MHz) and ^13^C NMR (150 MHz) (Table 1), IR (ATR) ν_max_ 3363, 2960, 1734, 1367, 1294, and 1018 cm^−1^, FABMS *m/z* 731 [M+Na]^+^, HRFABMS *m/z* 731.3105 [M+Na]^+^ (calcd. for C_32_H_52_O_17_Na, 731.3102).

### 3.7. Valerianairidoid IV (***6***)

Amorphous solid, [*α*]^25^_D_ −21.6 (*c* 0.5, MeOH), ^1^H NMR (CD_3_OD, 600 MHz) and ^13^C NMR (150 MHz) (Table 2), IR (ATR) ν_max_ 3363, 2927, 1748, 1664, 1362, and 1076 cm^−1^, ECD (MeOH) [201.0 nm (Δε −18.8)], FABMS *m/z* 499 [M+Na]^+^, HRFABMS *m/z* 499.2159 [M+Na]^+^ (calcd. for C_22_H_36_O_11_Na, 499.2155).

### 3.8. Valerianairidoid V (***7***)

Amorphous solid, [*α*]^25^_D_ −48.9 (*c* 0.3, MeOH), ^1^H NMR (CD_3_OD, 600 MHz) and ^13^C NMR (150 MHz) (Table 2); IR (ATR) ν_max_ 3336, 2923, 1741, 1362, 1267, and 1032 cm^−1^, ESI-MS *m/z* 661 [M+Na]^+^, HRESIMS *m/z* 661.2681 [M+Na]^+^ (calcd. for C_28_H_46_O_16_Na, 661.2678).

### 3.9. Valerianairidoid VI (***9***)

Amorphous solid, [*α*]^25^_D_ −15.4 (*c* 0.1, MeOH), ^1^H NMR (CD_3_OD, 600 MHz) and ^13^C NMR (150 MHz) (Table 3), IR (ATR) ν_max_ 3366, 2958, 1731, 1370, 1294, and 1032 cm^−1^, FABMS *m/z* 745 [M+Na]^+^, HRFABMS *m/z* 745.2881 [M+Na]^+^ (calcd. for C_32_H_50_O_18_Na, 745.2895).

### 3.10. Valerianairidoid VII (***12***)

Amorphous solid, [*α*]^25^_D_ −47.8 (*c* 0.2, MeOH), ^1^H NMR (CD_3_OD, 600 MHz) and ^13^C NMR (150 MHz) (Table 3), IR (ATR) ν_max_ 3356, 2924, 1745, 1670, 1362, and 1032 cm^−1^, ESIMS *m/z* 541 [M+Na]^+^, HRESIMS *m/z* 541.1893 [M+Na]^+^ (calcd. for C_23_H_34_O_13_Na, 541.1892).

### 3.11. Enzymatic Hydrolysis of Valerianairidoids I, II, IV, V, and VI (***1***, ***2***, ***6***, ***7***, and ***9***)

Enzymes [**1**, **6**, and **7**: *β*-glucosidase (5 mg, from sweet almond); **2** and **9**: cellulase (10 mg, from *Aspergillus niger*)] were added to a solution of the valerianairidoid [**1** and **7**: 5 mg in 100 mM acetate buffer (5.0 mL, pH 5.5); **2**: 5 mg in 50 mM citric acid–NaOH buffer (5.0 mL, pH 4.0); **6**: 10 mg in 100 mM acetate buffer (5.0 mL, pH 5.5); **9**: 10 mg in 50 mM citric acid–NaOH buffer (5.0 mL, pH 4.0)] and the mixture was stirred at 35 °C for 24 h. The supernatant solution was concentrated under vacuum to obtain a residue, which was subjected to HPLC {COSMOSIL 5C18-MS-II (250 × 4.6 mm), mobile phase: H_2_O–CH_3_CN (7:3, *v*/*v*)} to obtain the aglycone [**1**: **1a** (0.26 mg); **2**: **1a** (0.85 mg); **6**: **6a** (3.56 mg); **7**: **6a** (0.88 mg); **9**: **9a** (3.15 mg)].

### 3.12. Aglycone of Valerianairidoid I (***1a***)

Amorphous solid, [*α*]^25^_D_ −73.1 (*c* 0.1, MeOH), ^1^H NMR (CDCl_3_, 600 MHz) and ^13^C NMR (150 MHz) are identical to patrinoside-aglucone [16], FABMS *m/z* 323 [M+Na]^+^, HRFABMS *m/z* 323.1484 [M+Na]^+^ (calcd. for C_15_H_24_O_6_Na, 323.1471).

### 3.13. Aglycone of Valerianairidoid IV (***6a***)

Amorphous solid, [*α*]^25^_D_ −51.9 (*c* 0.1, MeOH), ^1^H NMR (CDCl_3_, 600 MHz), and ^13^C NMR (150 MHz) spectra (Table 2), FABMS *m/z* 337 [M+Na]^+^, HRFABMS *m/z* 337.1637 [M+Na]^+^ (calcd. for C_16_H_26_O_6_Na, 337.1627)

### 3.14. Aglycone of Valerianairidoid VI (***9a***)

Amorphous solid, [*α*]^25^_D_ −139.5 (*c* 0.1, MeOH), ^1^H NMR (CDCl_3_, 600 MHz) and ^13^C NMR (150 MHz) are identical with the aglycone of kanokoside A [18], FABMS *m/z* 337 [M+Na]^+^, HRFABMS *m/z* 337.1275 [M+Na]^+^ (calcd. for C_16_H_26_O_6_Na, 337.1263)

### 3.15. Calculating the Theoretical ECD Spectra of ***1a***, ***6a***, and ***9a***

The initial geometries of the conformers of 1*S*,5*S*,7*S*,8*S,*9*S*-**1a** were generated and their geometries optimized under vacuum conditions using the Merck molecular force field (MMFF) as implemented in the Spartan ’16 program [19]. Stable conformers with Boltzmann distributions over 1% were further optimized at the CAM-B3LYP/def2-TZVP level of density functional theory (DFT). Normal mode analysis was performed at the same level to confirm that none of the conformers show any imaginary frequencies and to obtain Gibbs free energies (*G*) [4,20]. Low free-energy conformers with Boltzmann distributions over 1% (Supplementary Materials) were subjected to ECD calculations using time-dependent DFT (TD-DFT) at the CAM-B3LYP/def2-TZVPP level. All DFT and TD-DFT calculations were performed using the integral equation formalism polarizable continuum model (IEFPCM) in MeOH within Gaussian 16 [21]. The resultant rotatory strengths of the lowest 30 excited states of each conformer were converted into Gaussian-type curves with half-bands (0.30 eV) using SpecDis v1.71 [22]. Theoretical ECD spectra were obtained after correction based on the Boltzmann distribution of the conformers and their relative free energies (Δ*G*). The ECD spectra of 1*S*,5*S*,7*S*,8*S,*9*S*-**6a** and 1*S*,5*S*,6*S*,7*S*,8*R,*9*S*-**9a** were also calculated according to the procedure described above (Figure 4).

### 3.16. Acid Hydrolyses of ***1***–***3***, ***6***, ***7***, ***9***, and ***12***

We used the method reported by Tanaka et al. [8] with slight modifications to determine the absolute configurations of the monosaccharide constituents of **1**–**3**, **6**, **7**, **9,** and **12**. A sample of **1**–**3**, **6**, **7**, **9**, or **12** (0.5 mg) was dissolved in 5% aqueous H_2_SO_4_–1,4-dioxane (1:1, *v*/*v*, 6.0 mL) and heated at 90 °C for 3 h. The solution was partitioned into ethyl acetate–H_2_O and the H_2_O fraction was neutralized. After drying in vacuo, the residue was dissolved in pyridine (0.5 mL) containing l-cysteine methyl ester hydrochloride (1.0 mg), and the mixture was heated at 60 °C for 1 h, after which phenyl isothiocyanate was added, and the mixture was heated at 60 °C for 1 h. The mixture was analyzed by reverse-phase HPLC {COSMOSIL 5C18-AR-II (250 × 4.6 mm i.d.), mobile phase: 0.3% CH_3_COOH–CH_3_CN (8:2, *v*/*v*); detection: UV (254 nm), flow rate: 1.0 mL/min, column temperature: 25 °C} to identify the d-glucose derivative by comparing the retention time with that of authentic samples (retention time: d-glucose, 33.4 min; l-glucose, 31.5 min).

### 3.17. Cells

Breast cancer (MDA-MB-231) and glioblastoma (U-251 MG) cells (American Type Culture Collection, Manassas, VA, USA) were cultured in DMEM/high glucose and DMEM/low glucose (Wako, Osaka, Japan), respectively, containing 10% fetal bovine serum (FBS; Sigma–Aldrich, St. Louis, MO, USA) and 1% penicillin/streptomycin (PC/SM; Wako).

### 3.18. Cell Viability Assay for non-CSCs

MDA-MB-231 and U-251 MG cells were seeded at a density of 3.0 × 10^3^ cells/90 μL per well in 96-well plates (3596; Corning, NY, USA) and treated with the test compounds (10 μL per well) 24 h after seeding. After 3 d, CellTiter-Glo^®^ 3D Reagent (Promega, Madison, WI, USA) was added to each well at an equivalent volume to the cell culture medium in the well, mixed by shaking for 5 min at room temperature, and incubated for 25 min at 37 °C under 5% CO_2_. The 100 μL supernatants were then transferred in technical replicates to 96-well white plates (136101; Thermo Fisher Scientific), and their luminescence was measured using a luminometer (GloMax^®^ Discover System; Promega).

### 3.19. Cell Viability Assay for CSCs

CSCs of MDA-MB-231 and U-251 MG were prepared using a sphere-formation assay, as described previously [13]. Briefly, cells were cultured in DMEM/F12 (Thermo Fisher Scientific, Waltham, MA, USA) containing 1% PC/SM, 2% B-27 [B-27^®^ Serum-Free Supplement (50×); Thermo Fisher Scientific], 20 ng/mL epidermal growth factor (EGF; Peprotech, Rocky Hill, NJ, USA), and 20 ng/mL basic fibroblast growth factor (b-FGF; Peprotech) for 7 d in ultra-low attachment six-well plates (Corning International, Corning, NY, USA). Cell viability was evaluated using the CellTiter-Glo^®^ 3D Reagent in the same manner as described for the non-CSCs.

### 3.20. Statistics

Statistical analyses were performed using GraphPad Prism 8.21 software using one-way Analysis Of Variance (ANOVA) followed by the Dunnett’s test to analyze differences between the treatment groups. Differences were considered significant at: * *p* < 0.05, ** *p* <0.01, or *** *p* < 0.001.

## 4. Conclusions

We isolated seven new iridoid glucosides, namely the valerianairidoids I–VII (**1**–**3**, **6**, **7**, **9**, and **12**), and six known compounds from the MeOH extract of the dried rhizomes and roots of *V. fauriei*. Chemical structures, including the absolute configurations of the new compounds, were elucidated by NMR, MS, and ECD spectroscopy, and derivatization. The isolated iridoid glycosides did not show any anti-proliferative activity against MDA-MB-231 and U-251MG cells or their CSCs. On the other hand, aglycones **1a**, **6a**, and **9a** showed selective anti-proliferative activities against CSCs from MDA-MB-231 cells. Therefore, these aglycones have potential as breast cancer and preventive agents that may inhibit the characteristic signaling pathway(s) in CSCs.

## Figures and Tables

**Table 1:** ^13^C (150 MHz) and ^1^H (600 MHz) NMR data for valerianairidoids I–III (**1**–**3**) in CD_3_OD.

Position	1		2		3	
	*δ*C	*δ*H *(J* in Hz)	*δ*C	*δ*H *(J* in Hz)	*δ*C	*δ*H *(J* in Hz)
1	93.6	5.89 (d, 5.5)	93.5	5.91 (d, 5.5)	93.3	5.93 (d, 5.5)
3	140.1	6.37 (s)	140.2	6.39 (s)	140.2	6.41 (s)
4	116.4		116.3		116.2	
5	34.1	3.01 (dd-like, 7.6, 15.8)	34.1	3.01 (m)	33.9	3.03 (dd-like, 8.0, 15.6)
6	40.9	*α* 1.82 (m) *β* 2.06 (m)	40.9	*α* 1.84 (m) *β* 2.05 (m)	40.7	*α* 1.87 (m) *β* 2.09 (m)
7	73.3	4.33 (m)	73.3	4.32 (m)	72.4	4.26 (m)
8	49.8	1.94 (m)	49.0 (overlapping)	1.95 (m)	43.4	2.16 (m)
9	42.6	2.17 (m)	42.7	2.18 (m)	46.2	2.09 (m)
10	62.2	a 3.73 (dd, 5.5, 11.0) b 3.80 (m)	62.3	a 3.69 (dd, 5.5, 11.0) b 3.81 (dd, 5.5, 11.0)	69.8	a 3.79 (d-like, 11.6) b 4.14 (d-like, 11.6)
11	69.8	a 4.08 (d, 11.0) b 4.23 (d, 11.0)	69.8	a 4.06 (d, 11.7) b 4.25 (d, 11.7)	69.7	a 4.06 (d, 11.7) b 4.26 (d, 11.7)
1′	103.2	4.31 (d, 7.6)	103.3	4.28 (d, 7.6)	103.3	4.29 (d, 8.4)
2′	74.9	3.20 (m)	75.0	3.21 (m)	75.0	3.21 (m)
3′	76.4	3.52 (t, 7.6)	77.8	3.28 (m)	77.0	3.46 (m)
4′	80.7	3.57 (t, 7.6)	71.6	3.30 (m)	71.5	3.31 (m)
5′	78.1	3.35 (m)	77.0	3.47 (m)	77.9	3.35 (m)
6′	62.4	a 3.65 (m) b 3.66 (m)	70.2	a 3.73 (dd, 6.9, 11.7) b 4.09 (dd, 2.0, 11.7)	64.7	a 4.20 (dd, 7.2, 11.2) b 4.29 (d-like, 11.2)
1″	104.6	4.40 (d, 7.6)	105.0	4.39 (d, 7.5)	104.8	4.40 (d, 8.2)
2″	74.8	3.20 (m)	75.1	3.21 (m)	75.0	3.21 (m)
3″	77.8	3.31 (m)	78.0	3.28 (m)	77.0	3.46 (m)
4″	71.3	3.28 (m)	71.7	3.34 (m)	71.5	3.35 (m)
5″	76.4	3.38 (m)	75.4	3.46 (m)	77.9	3.28 (m)
6″	61.9	a 3.73 (m) b 3.81 (m)	64.5	a 4.19 (dd, 6.2, 11.7) b 4.42 (dd, 2.0, 11.7)	62.7	a 3.68 (dd, 9.0, 11.0) b 3.86 (d-like, 11.0)
1‴	173.3		173.3		173.2	
2‴	44.1	2.23 (d, 6.9)	44.2	2.23 (d, 7.6)	44.2	2.25 (d, 7.2)
3‴	26.8	2.09 (m)	26.8	2.08 (m)	26.7	2.09 (m)
4‴	22.6	0.96 (d, 6.9)	22.7	0.97 (d, 6.8)	22.6	0.95 (d, 6.9)
5‴	22.6	0.96 (d, 6.9)	22.7	0.97 (d, 6.8)	22.6	0.95 (d, 6.9)
1′′′′			174.7		174.8	
2′′′′			44.2	2.14 (d, 6.9)	44.1	2.21 (d, 7.0)
3′′′′			26.8	1.94 (m)	26.7	2.09 (m)
4′′′′			22.7	0.87 (d, 6.9)	22.6	0.95 (d, 6.9)
5′′′′			22.7	0.87 (d, 6.9)	22.6	0.95 (d, 6.9)

**Table 2:** ^13^C (150 MHz) and ^1^H (600 MHz) NMR data for valerianairidoid IV (**6**), its aglycone **6a**, and valerianairidoid V (**7**).

Position	6	(CD_3_OD)	6a	(CDCl_3_)	7	(CD_3_OD)
	*δ*C	*δ*H *(J* in Hz)	*δ*C	*δ*H *(J* in Hz)	*δ*C	*δ*H *(J* in Hz)
1	93.6	5.88 (d, 5.5)	91.6	5.85 (d, 5.5)	93.5	5.90 (d, 5.5)
3	140.1	6.35 (s)	138.3	6.34 (s)	140.2	6.40 (s)
4	116.4		117.2		116.3	
5	34.1	2.99 (dd-like, 7.8, 15.6)	32.5	3.03 (dd, 7.8, 15.1)	34.1	3.01 (dd-like, 7.6, 15.8)
6	40.9	*α* 1.79 (m) *β* 2.04 (ddd, 3.0, 7.8, 13.8)	40.4	*α* 1.87 (m) *β* 2.07 (ddd, 3.4, 6.9, 13.1)	40.9	*α* 1.86 (m) *β* 2.07 (ddd, 2.0, 6.8, 13.2)
7	73.3	4.30 (m)	74.1	4.48 (dd-like, 5.5, 8.9)	73.3	4.33 (m)
8	49.0 (overlapping)	1.93 (m)	46.3	2.00 (m)	49.0 (overlapping)	1.95 (m)
9	42.2	2.14 (m)	39.9	2.42 (m)	42.6	2.18 (m)
10	62.2	a 3.63 (dd, 5.4, 10.8) b 3.70 (dd, 5.4, 10.8)	61.8	a 3.83 (m) b 4.00 (m)	62.2	a 3.73 (dd, 5.5, 10.3) b 3.82 (dd, 7.6, 10.3)
11	69.7	a 4.05 (d, 11.4) b 4.24 (d, 11.4)	62.5	a 4.00 (m) b 4.09 (d, 6.8)	69.8	a 4.07 (d, 11.7) b 4.25 (d, 11.7)
1′	103.4	4.26 (d, 7.2)			103.3	4.29 (d, 8.2)
2′	75.1	3.17 (dd, 7.2, 9.0)			75.0	3.21 (m)
3′	78.0	3.24 (m)			77.9	3.37 (m)
4′	71.7	3.28 (dd, 1.8, 3.6)			71.5	3.31 (m)
5′	78.1	3.32 (m)			77.0	3.46 (m)
6′	62.8	a 3.81 (m) b 3.83 (m)			69.9	a 3.77 (dd, 6.2, 10.3) b 4.14 (d-like, 10.3)
1″					104.9	4.40 (d, *J* = 7.6)
2″					75.0	3.21 (m)
3″					77.9	3.37 (m)
4″					71.5	3.31 (m)
5″					77.9	3.37 (m)
6″					62.7	a 3.67 (dd, 5.5, 11.0) b 3.86 (d-like, 11.0)
1‴	173.5		172.3		173.6	
2‴	42.7	a 2.12 (m) b 2.33 (d, 6.0, 15.0)	41.4	2.17 (dd, 8.3, 15.1) 2.36 (dd, 6.2, 15.1)	42.2	2.18 (m) 2.35 (dd, 6.2, 15.1)
3‴	33.2	1.84 (m)	31.8	1.89 (m)	33.1	1.86 (m)
4‴	30.3	a 1.24 (m) b 1.37 (m)	29.2	a 1.25 (m) b 1.38 (m)	30.2	a 1.25 (m) b 1.39 (m)
5‴	11.6	0.89 (t, 7.6)	11.2	0.90 (t, 7.6)	11.6	0.91 (t, 6.9)
6‴	19.5	0.92 (d, 6.8)	19.2	0.95 (d, 6.8)	19.5	0.95 (d, 6.8)

**Table 3:** ^13^C (150 MHz) and ^1^H NMR (600 MHz) data for valerianairidoids VI and VII (**9** and **12**) recorded in CD_3_OD.

Position	9		12	
	*δ*C	*δ*H *(J* in Hz)	*δ*C	*δ*H *(J* in Hz)
1	90.5	6.30 (br-s)	89.9	6.38 (d, 5.5)
3	142.8	6.35 (s)	141.4	6.35 (s)
4	109.2		108.3	
5	35.4	2.98 (d-like, 8.3)	34.3	2.96 (d-like, 8.3)
6	59.7	3.95 (d-like, 2.1)	58.8	3.82 (m)
7	60.2	3.26 (m)	59.7	3.36 (d, 2.8)
8	80.1		79.4	
9	43.4	1.93 (m)	42.7	2.00 (m)
10	67.0	a 3.59 (d, 5.5) b 3.59 (d, 5.5)	66.5	a 3.68 (m) b 3.68 (m)
11	69.7	a 4.12 (d, 11.6) b 4.24 (d, 11.6)	69.0	a 4.22 (d, 11.7) b 4.30 (d, 11.7)
1′	102.2	4.29 (d, 8.3)	99.6	4.60 (d, 8.4)
2′	75.1	3.13 (m)	74.8	4.74 (dd, 8.4, 9.6)
3′	77.9	3.27 (m)	75.6	3.53 (dd, 8.9, 9.6)
4′	71.6	3.21 (m)	71.2	3.34 (m)
5′	77.0	3.38 (m)	77.5	3.33 (m)
6′	70.0	a 3.64 (dd, 2.0, 11.7) b 4.01 (dd, 6.2, 11.7)	62.1	a 3.67 (m) b 3.87 (dd, 2.4, 12.0)
1″	104.9	4.30 (d, 7.6)	172.4	
2″	75.1	3.12 (m)	20.4	2.05 (s)
3″	78.1	3.10 (m)		
4″	71.7	3.21 (m)		
5″	75.4	3.36 (m)		
6″	64.5	a 4.12 (dd, 2.0, 8.3) b 4.33 (dd, 2.0, 11.6)		
1‴	173.0		171.3	
2‴	44.1	2.08 (d, 7.6)	43.5	2.17 (dd, 4.1, 6.9)
3‴	26.9	1.99 (m)	26.3	2.02 (m)
4‴	22.8	0.84 (d, 6.8)	22.0	0.94 (d, 6.9)
5‴	22.8	0.84 (d, 6.8)	22.0	0.94 (d, 6.9)
1′′′′	174.7			
2′′′′	44.2	2.14 (d, 6.9)		
3′′′′	26.8	1.94 (m)		
4′′′′	22.6	0.84 (d, 6.9)		
5′′′′	22.6	0.84 (d, 6.9)		

## Data Availability

Data supporting the findings of this study are available from the corresponding author upon reasonable request.

## Supplementary Materials

The following supporting information can be downloaded at: https://www.mdpi.com/article/10.3390/ijms232214206/s1.
