# Supplementary material for: Anti-Proliferative Effects of Iridoids from Valeriana fauriei on Cancer Stem Cells
Source: Int J Mol Sci. 2022 Nov 17;23(22):14206. doi: 10.3390/ijms232214206 (PMC9698980; doi:10.3390/ijms232214206)
Supplement: Supplementary file 1 [file ijms-23-14206-s001.zip › Supplementary Materials.pdf]

Supplementary Materials

Anti-proliferative effects on cancer stem cells of derivatives of iridoid glucosides isolated from *Valeriana fauriei*

Hayato Yoshikawa[1], Takahiro Matsumoto[1*], Takahiro Kitagawa[1], Tomoe Ohta[2], and Tatsusada Yoshida[2], Tetsushi Watanabe[a*]

[1]*Kyoto Pharmaceutical University, 1 Misasagi-Shichono-cho, Yamashina-ku, Kyoto 607-8412, Japan.*

[2]*Faculty of Pharmaceutical Sciences, Nagasaki International University, Nagasaki 859-3298, Japan*

List of Supplementary Materials

S1. ^{1}H NMR spectra of new compounds **1**–**3**, **6**, **6a,** **7**, **9**, and **12**

S2. ^{13}C NMR spectra of new compounds **1**–**3**, **6**, **6a**, **7**, **9**, and **12**

S3. 2D NMR spectra of new compounds **1**–**3**, **6**, **6a**, **7**, **9**, and **12**

S4. Optimized geometries, the minimum value of frequency, relative free-energies, and Boltzmann distributions of conformers of **1a**, **6a**, and **9a**.

S5. Anti-proliferative activity of **1a**, **6a**, and **9a** against non-CSCs and CSCs from MDA-MB-231 cells.

S1. ^{1}H NMR spectra of new compounds 1–3, 6, 6a, 7, 9, and 12

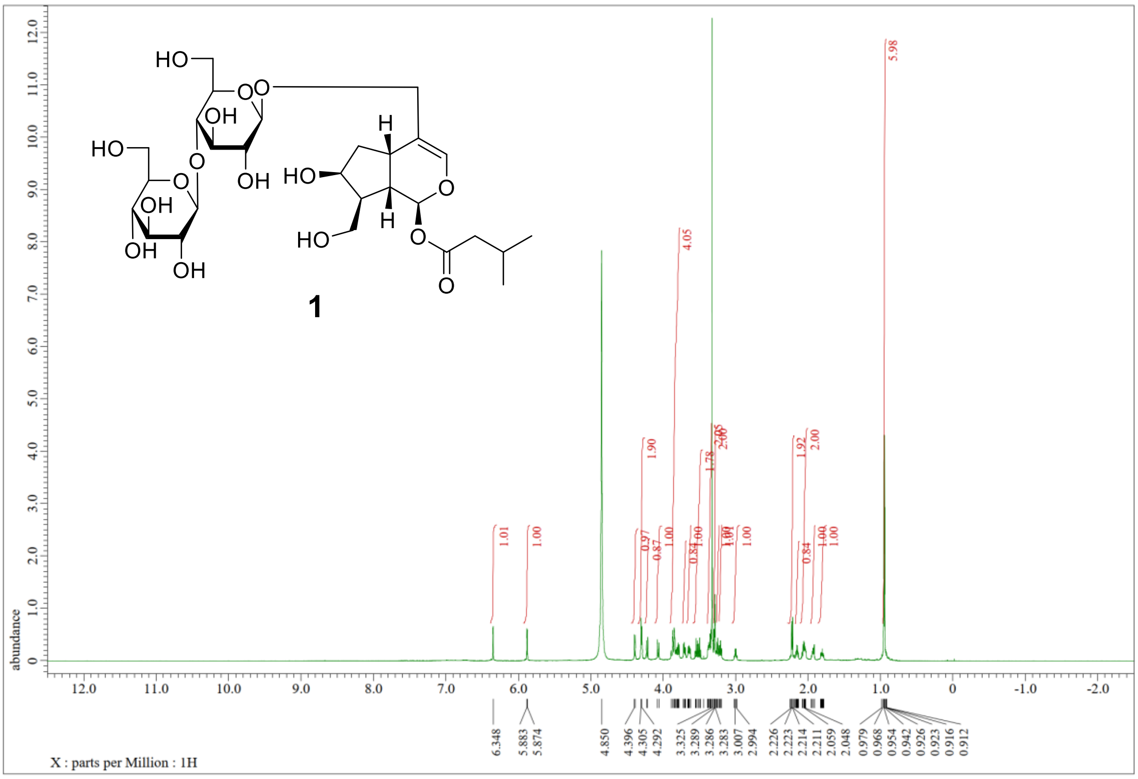

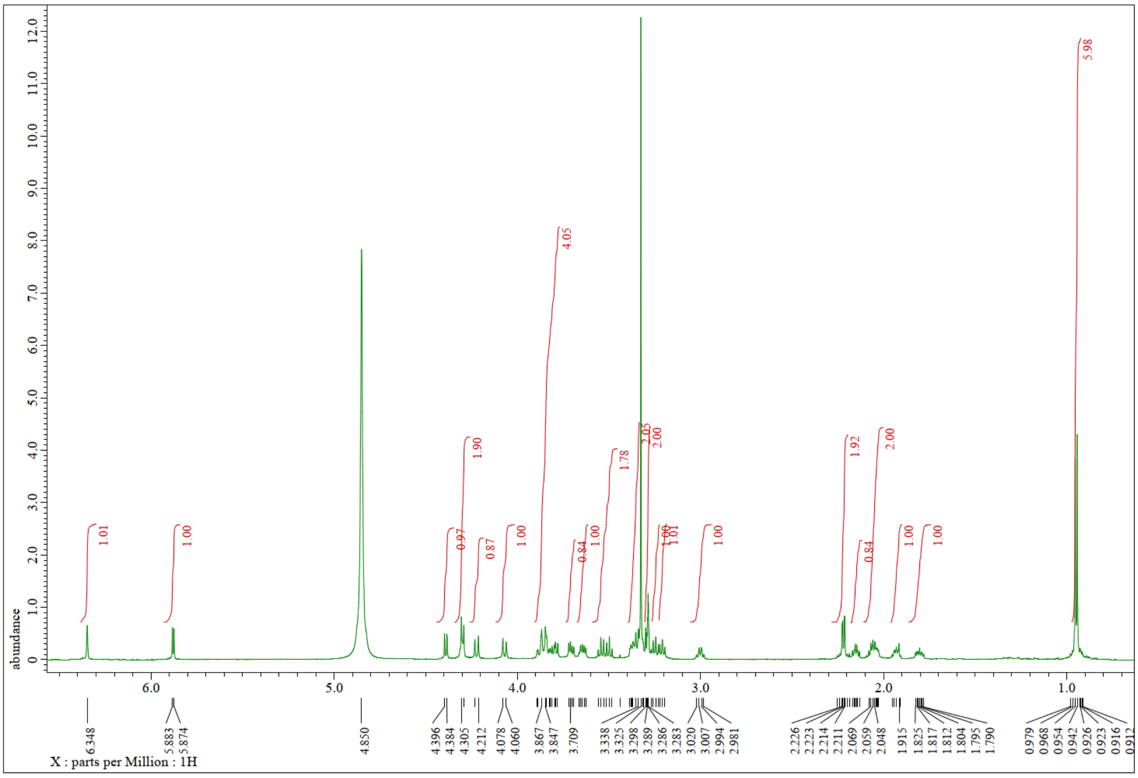

^{1}H NMR spectrum of **1**

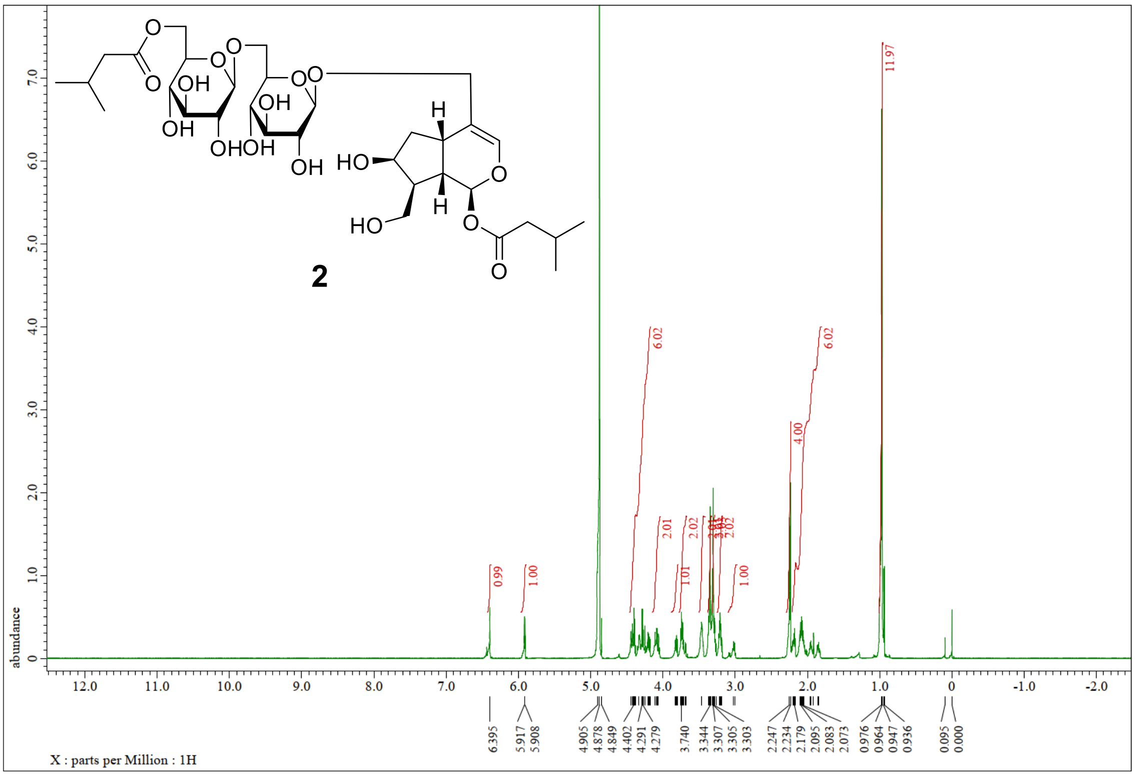

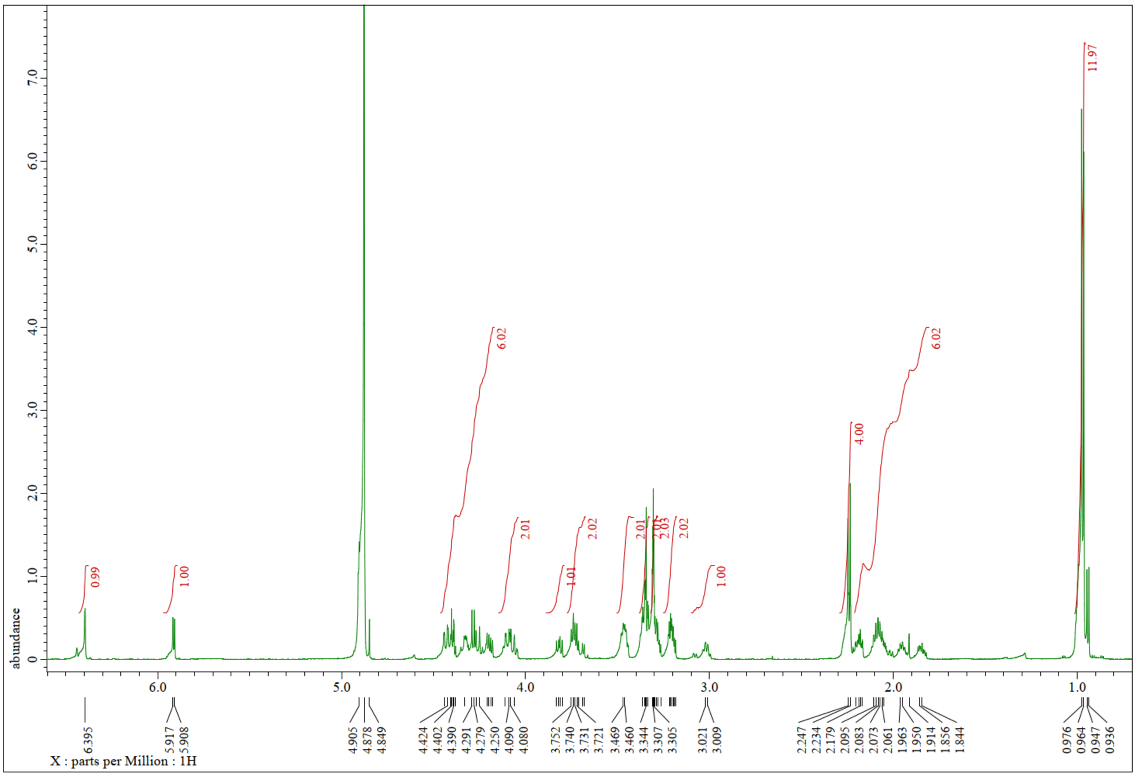

^{1}H NMR spectrum of **2**

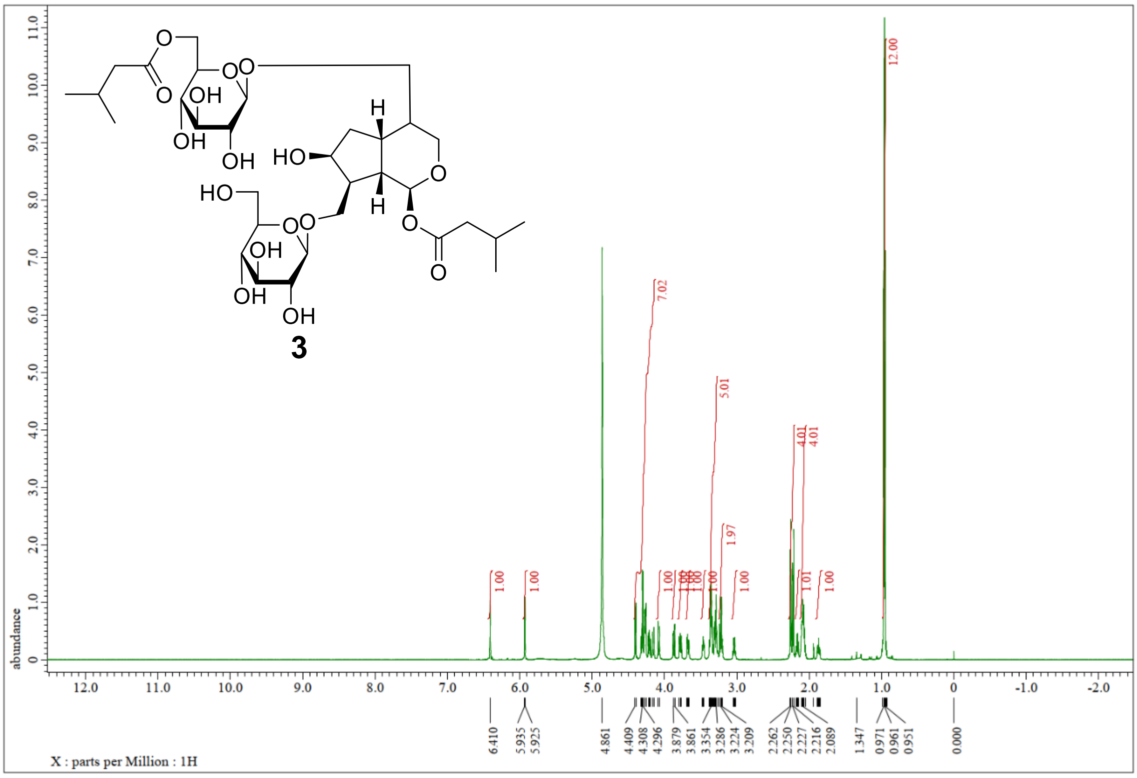

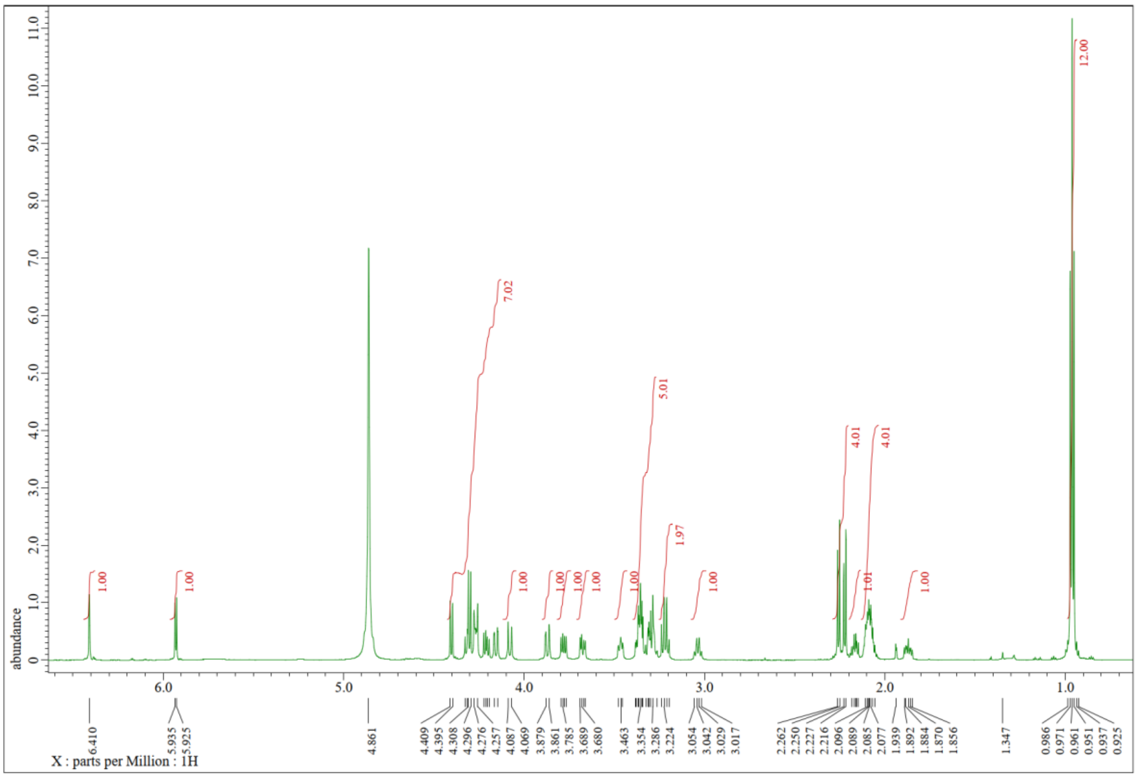

^{1}H NMR spectrum of **3**

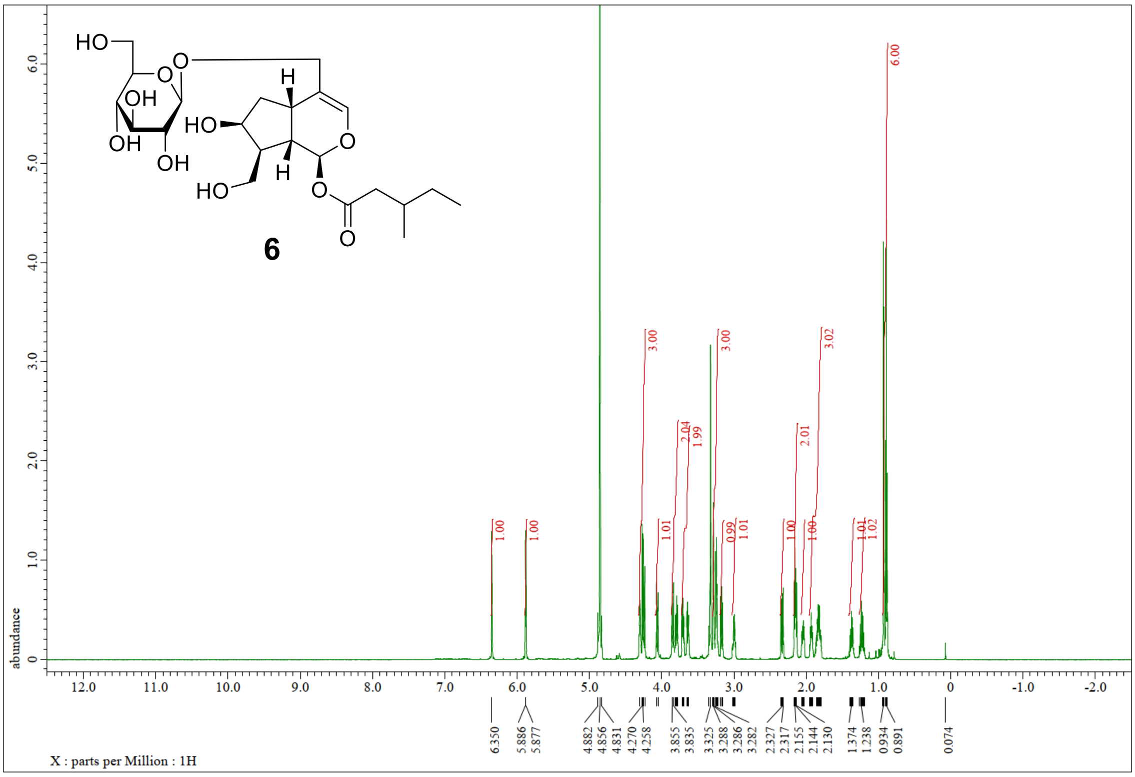

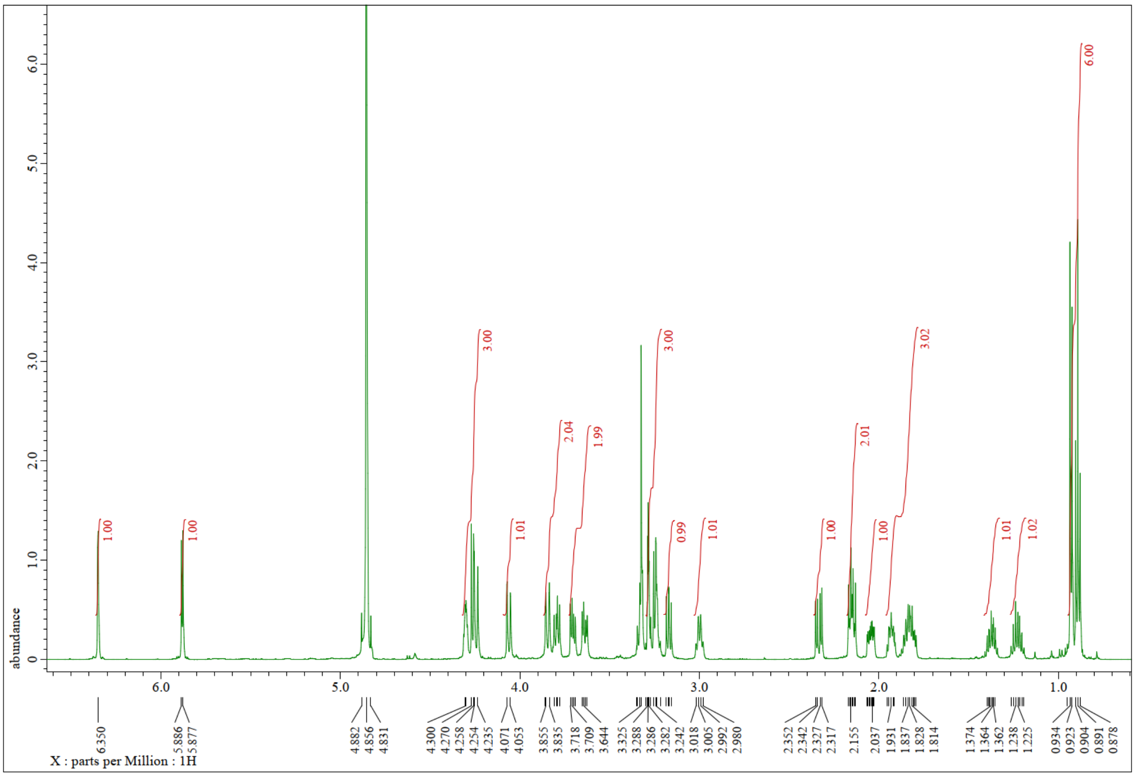

^{1}H NMR spectrum of **6**

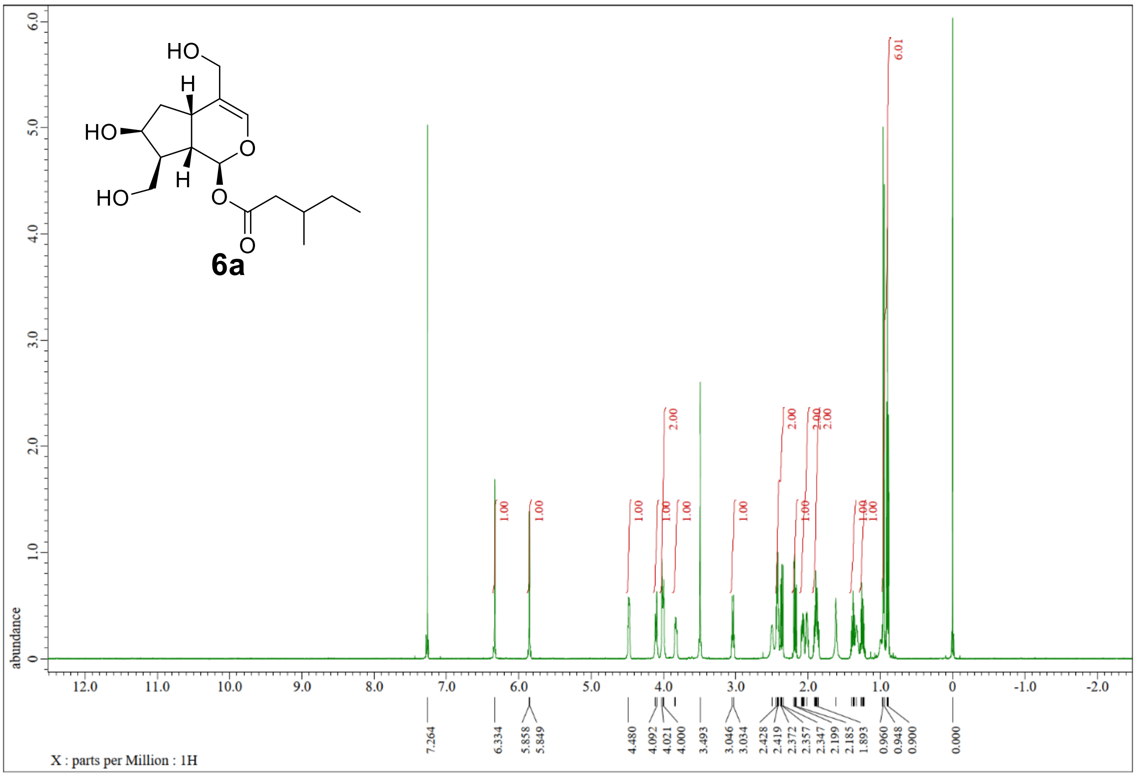

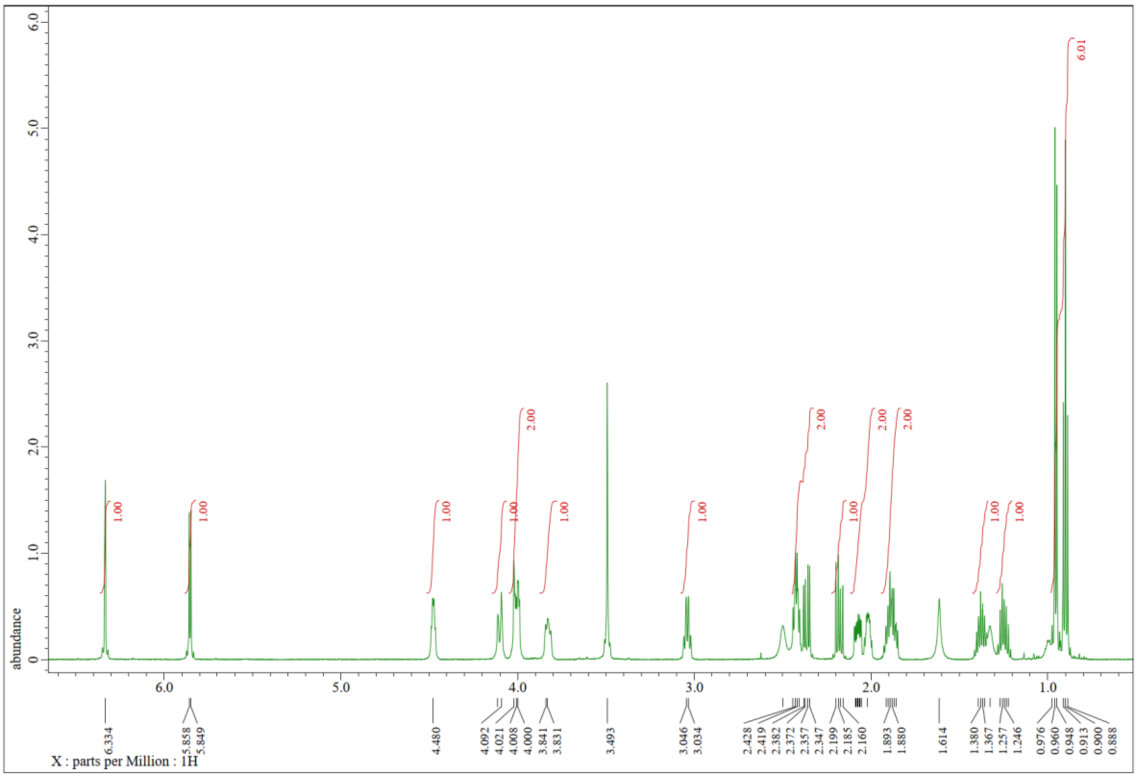

^{1}H NMR spectrum of **6a**

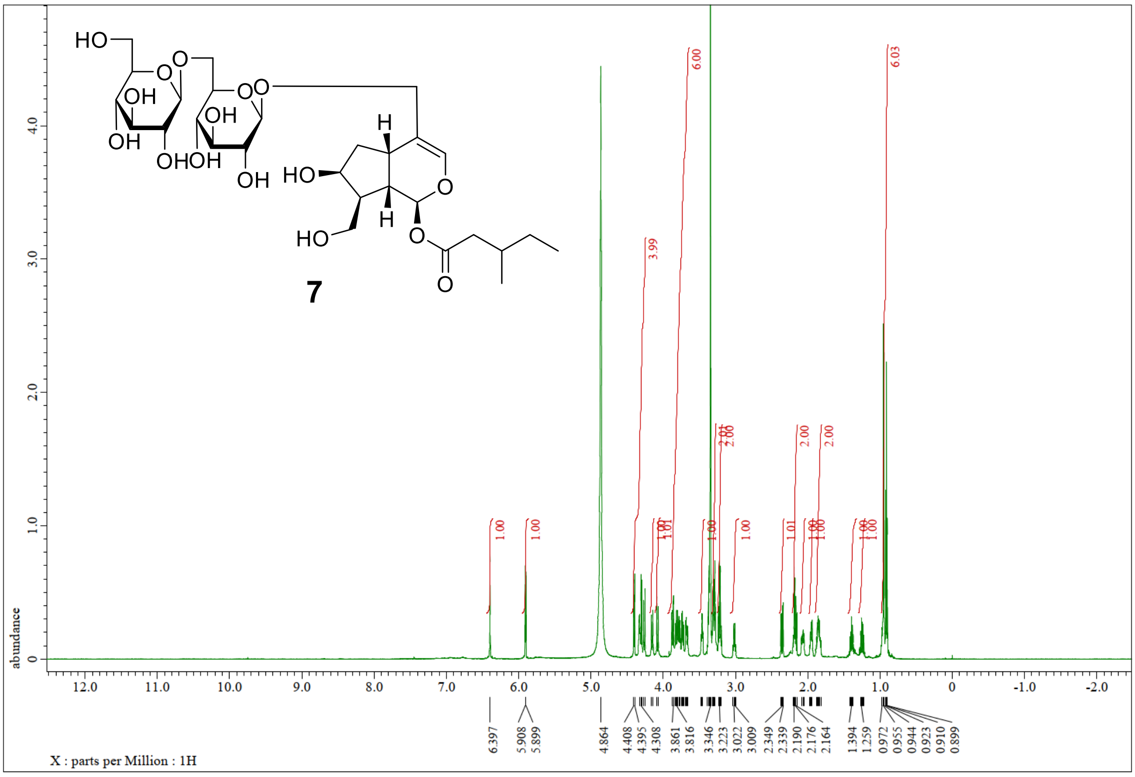

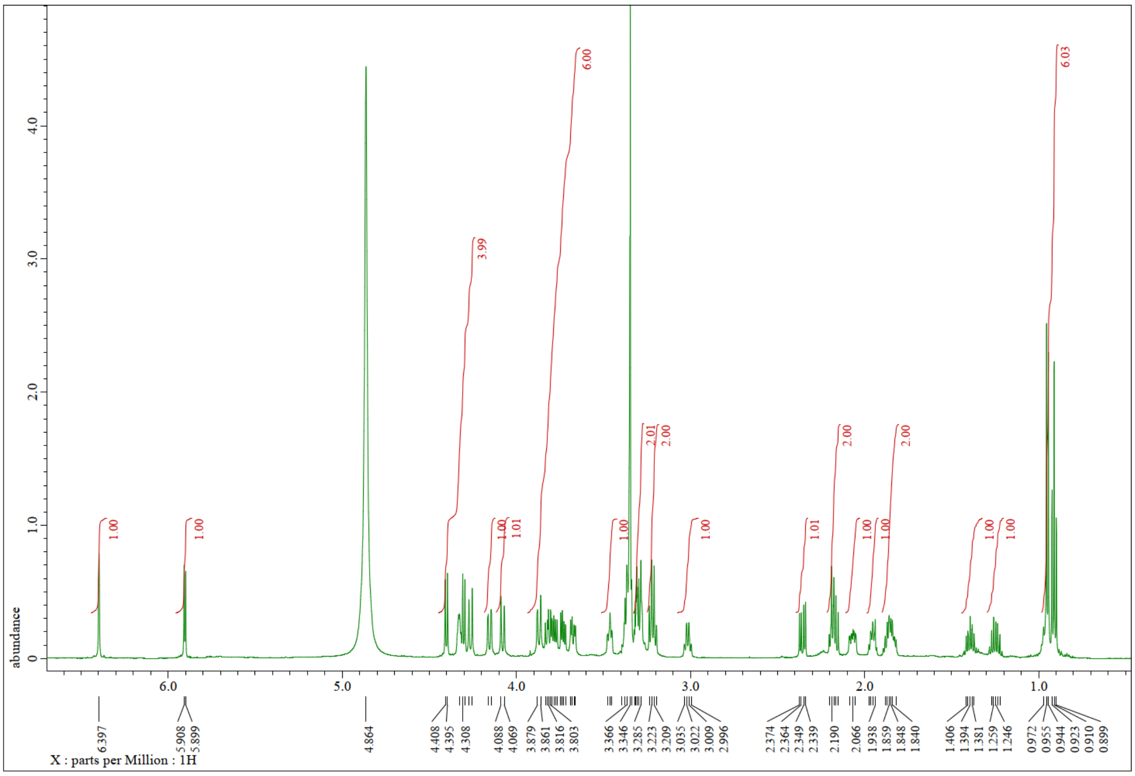

^{1}H NMR spectrum of **7**

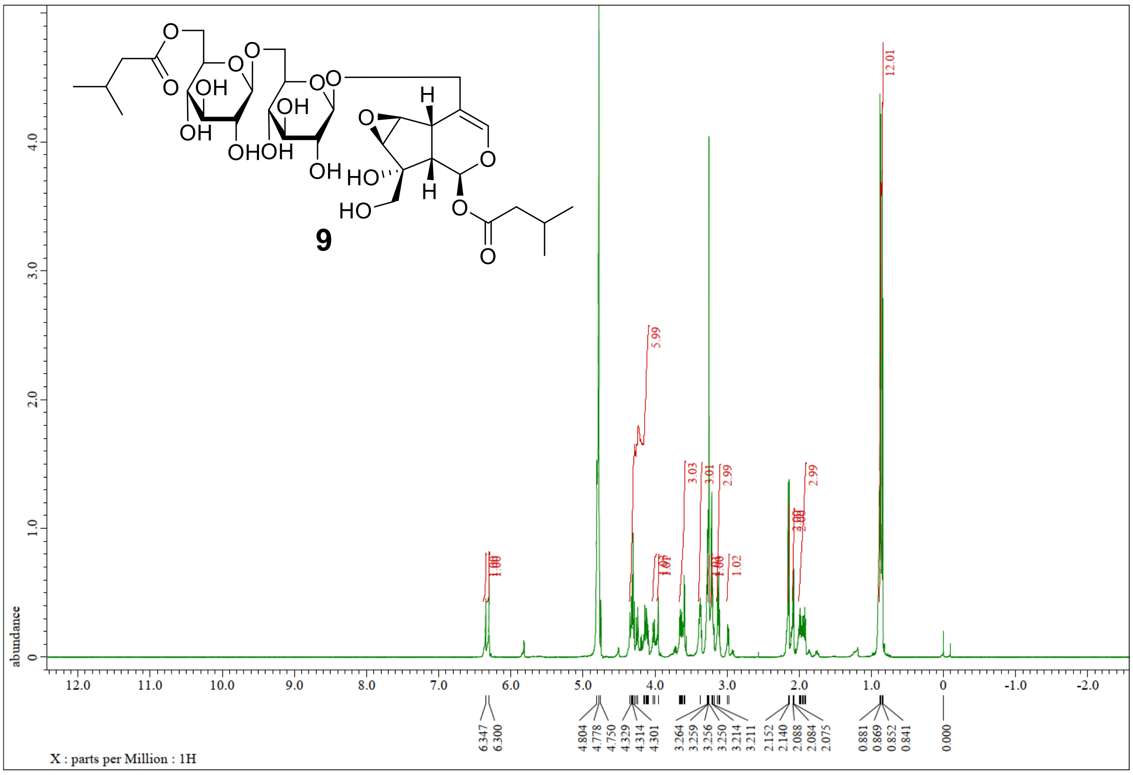

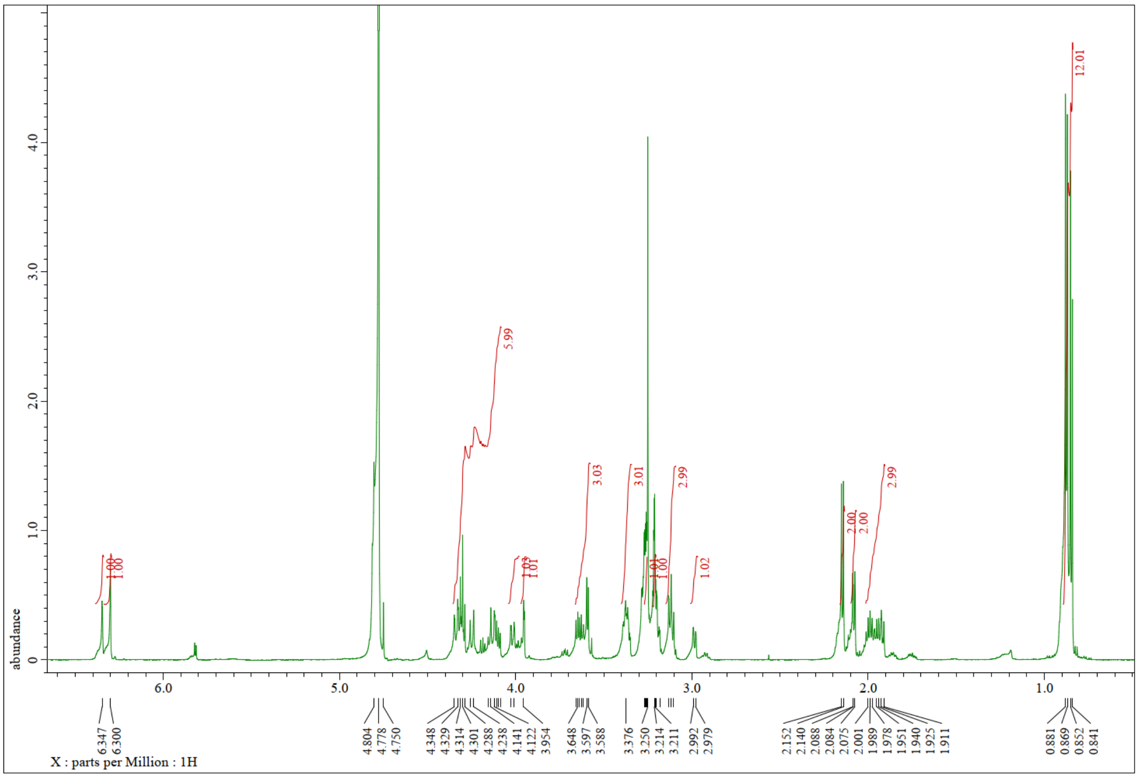

^{1}H NMR spectrum of **9**

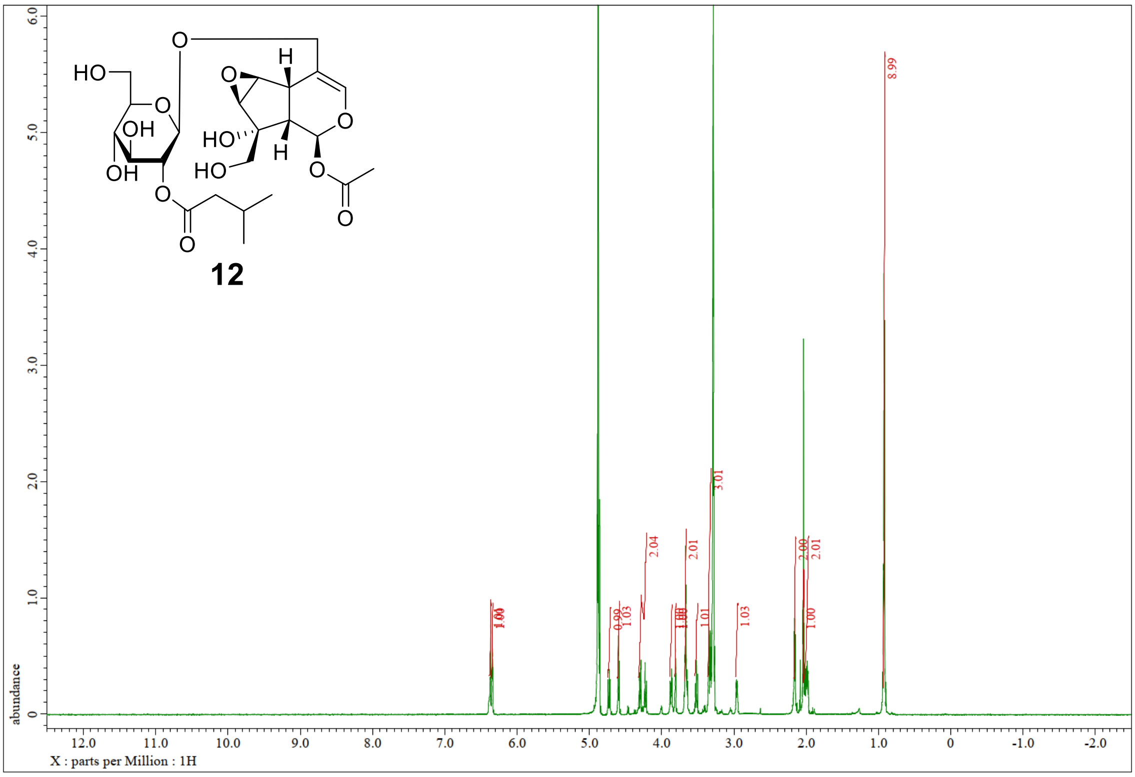

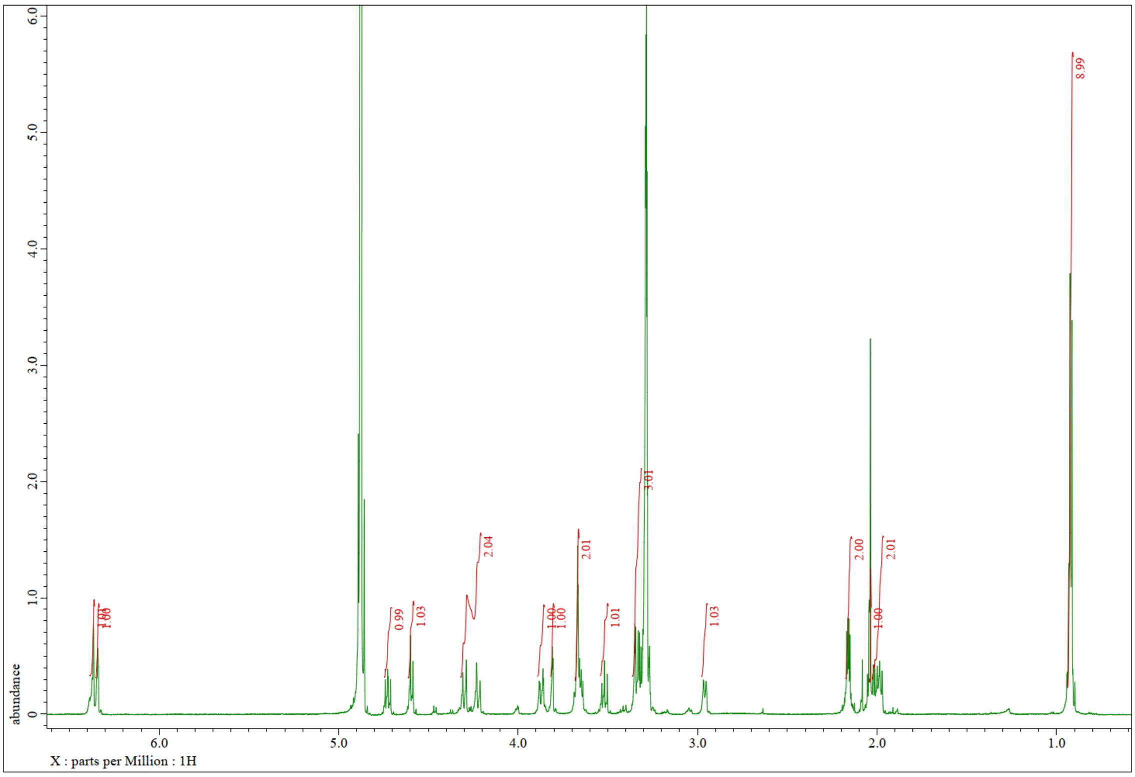

^{1}H NMR spectrum of **12**

S2. ^{13}C NMR spectra of new compounds 1–3, 6, 6a,7, 9, and 12

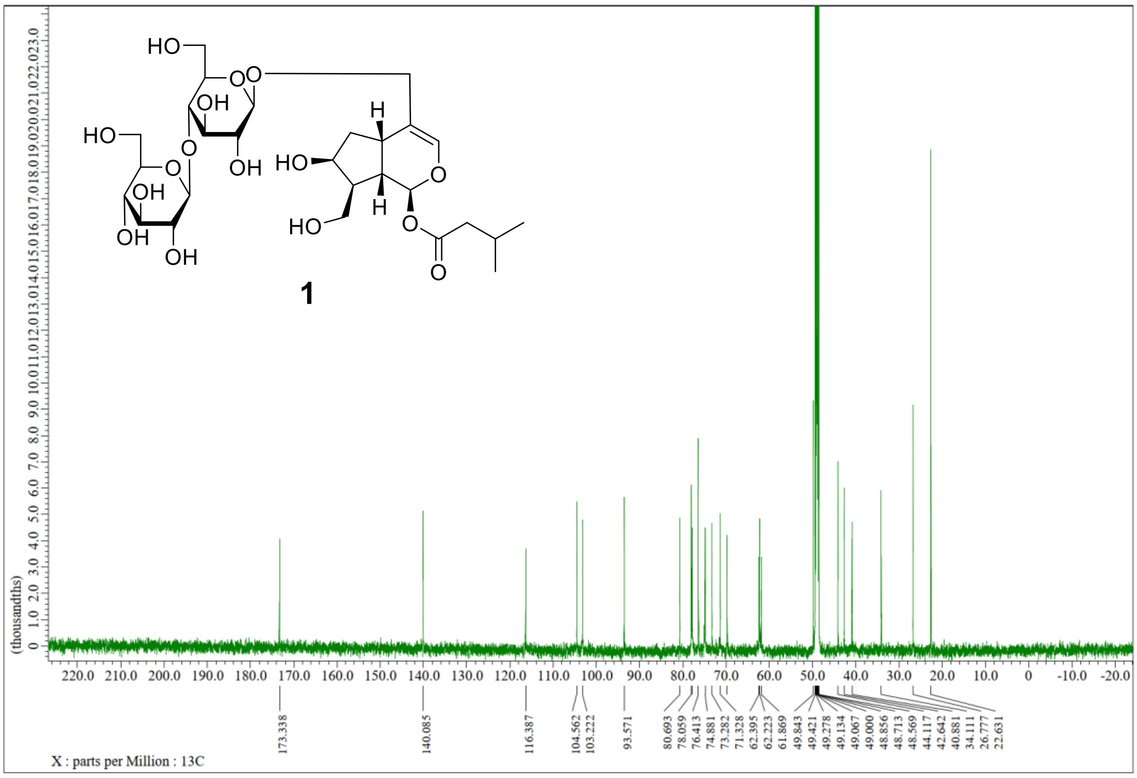

^{13}C NMR spectrum of **1**

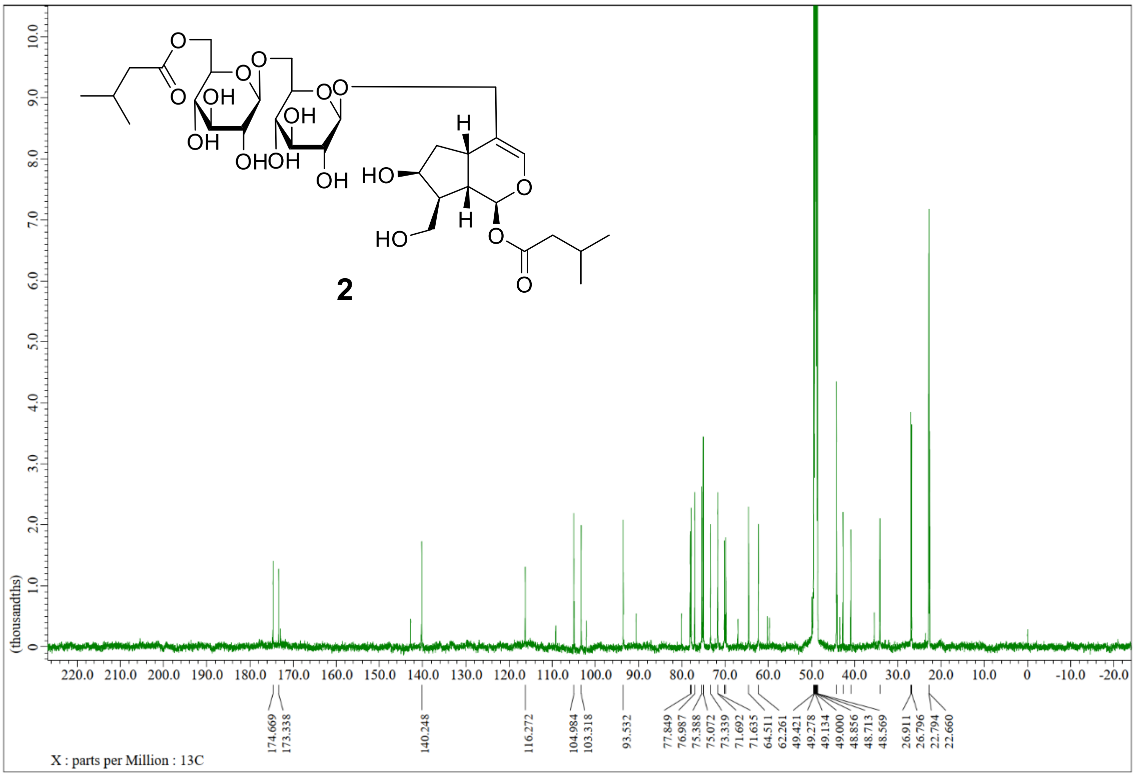

^{13}C NMR spectrum of **2**

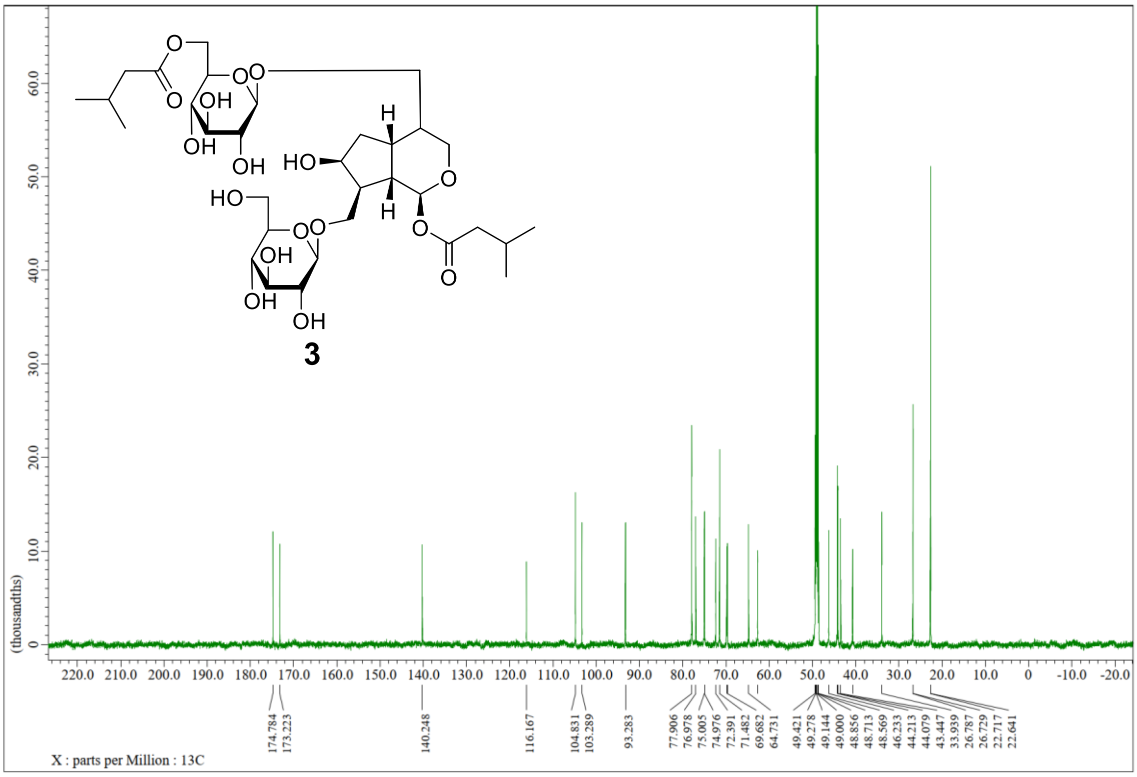

^{13}C NMR spectrum of **3**

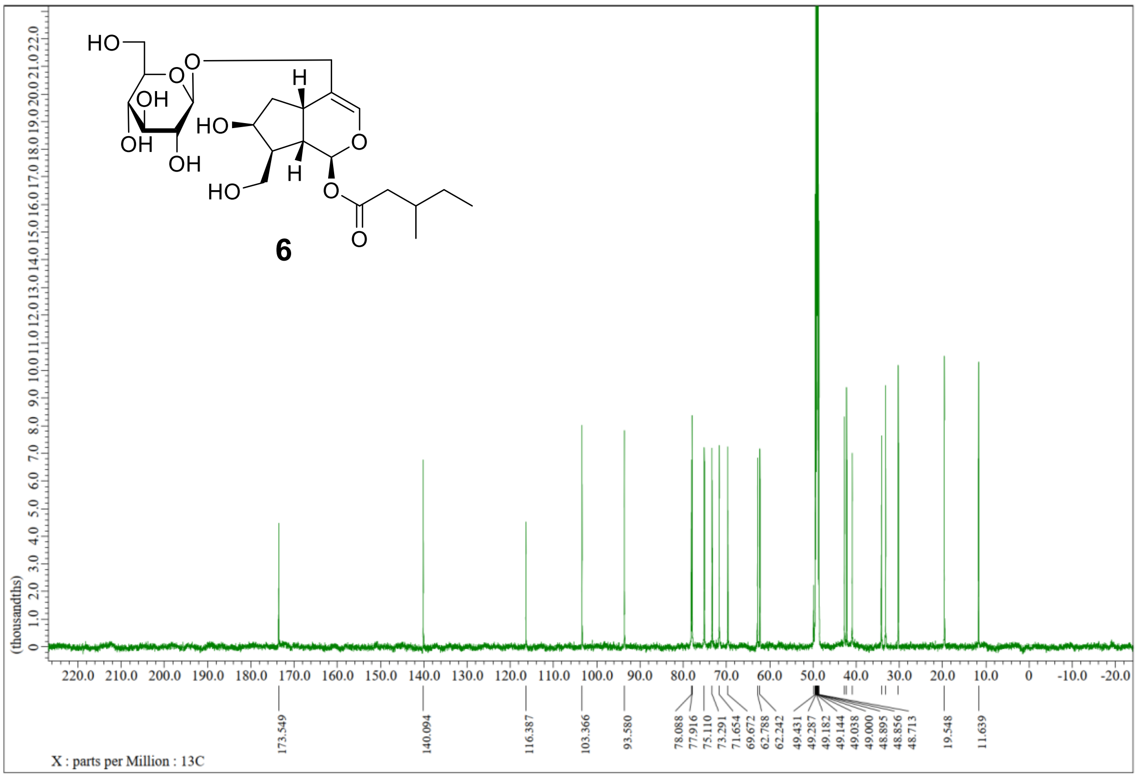

^{13}C NMR spectrum of **6**

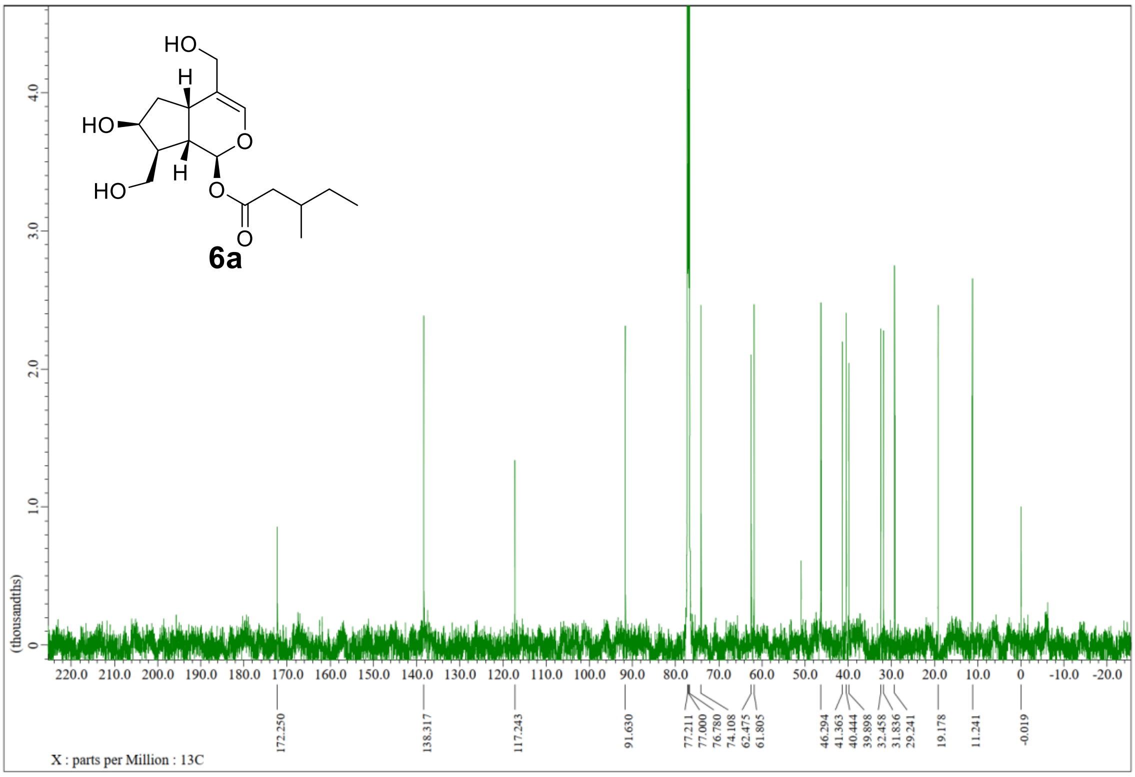

^{13}C NMR spectrum of **6a**

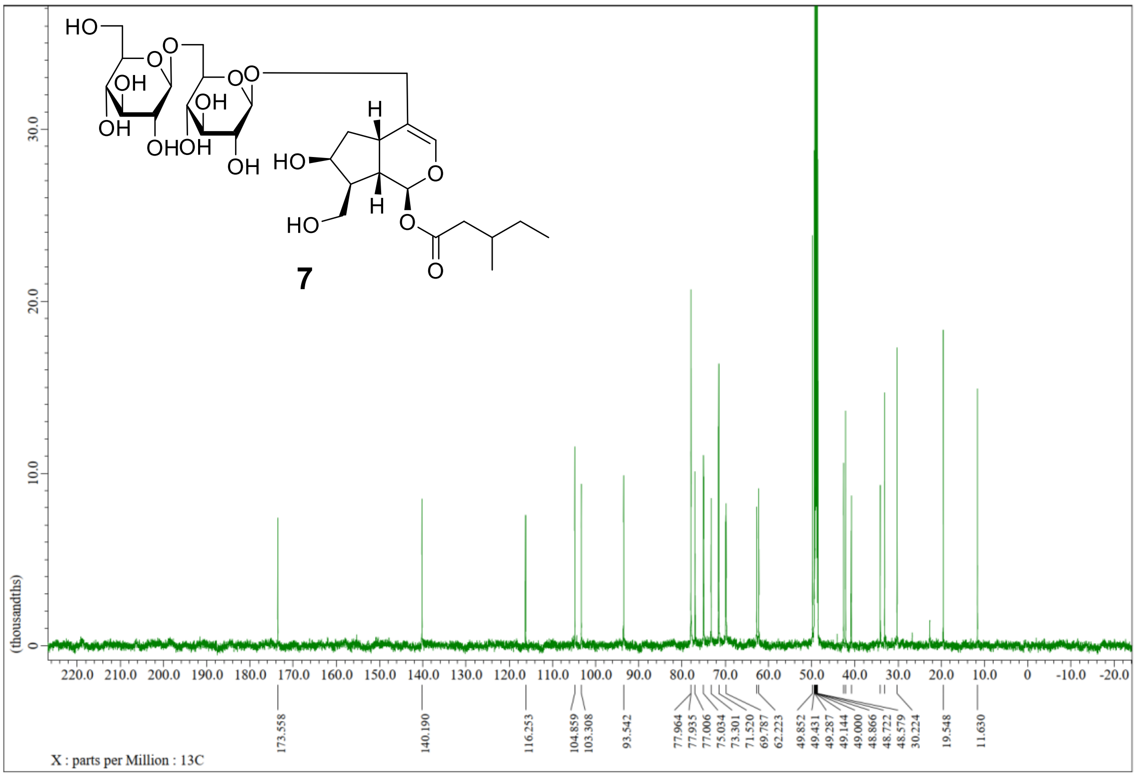

^{13}C NMR spectrum of **7**

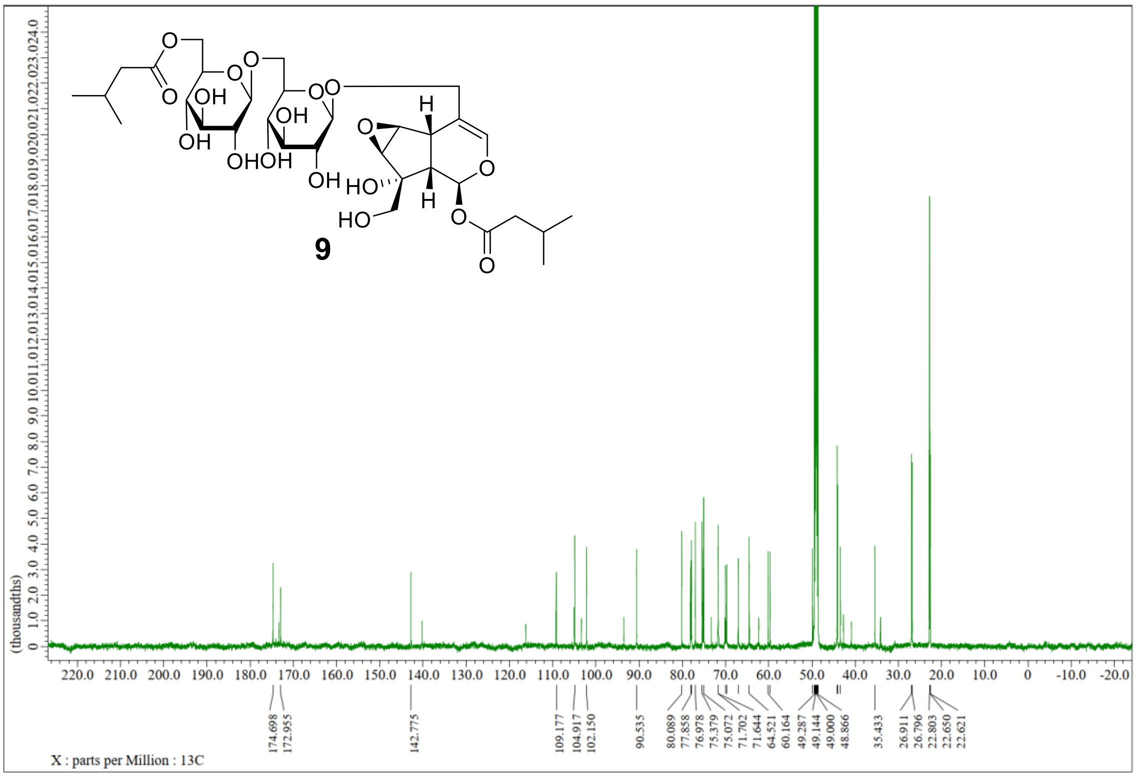

^{13}C NMR spectrum of **9**

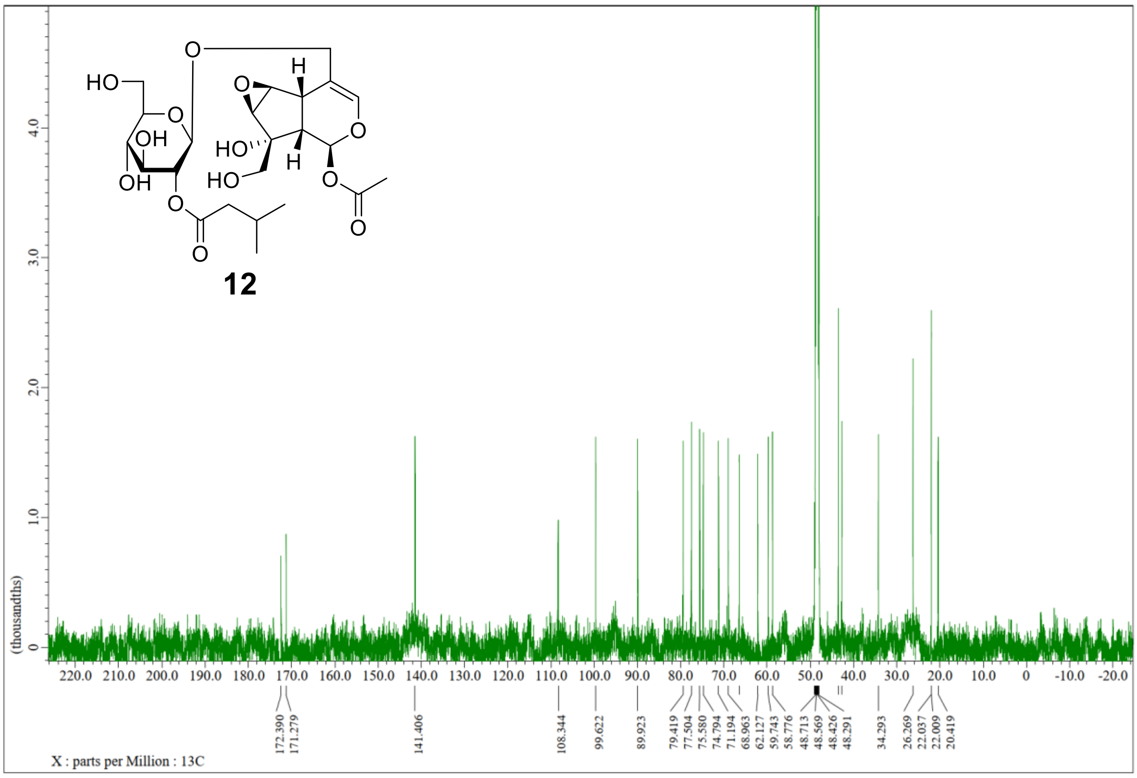

^{13}C NMR spectrum of **12**

S3. 2DNMR spectra of new compounds 1–3, 6, 6a, 7, 9, and 12

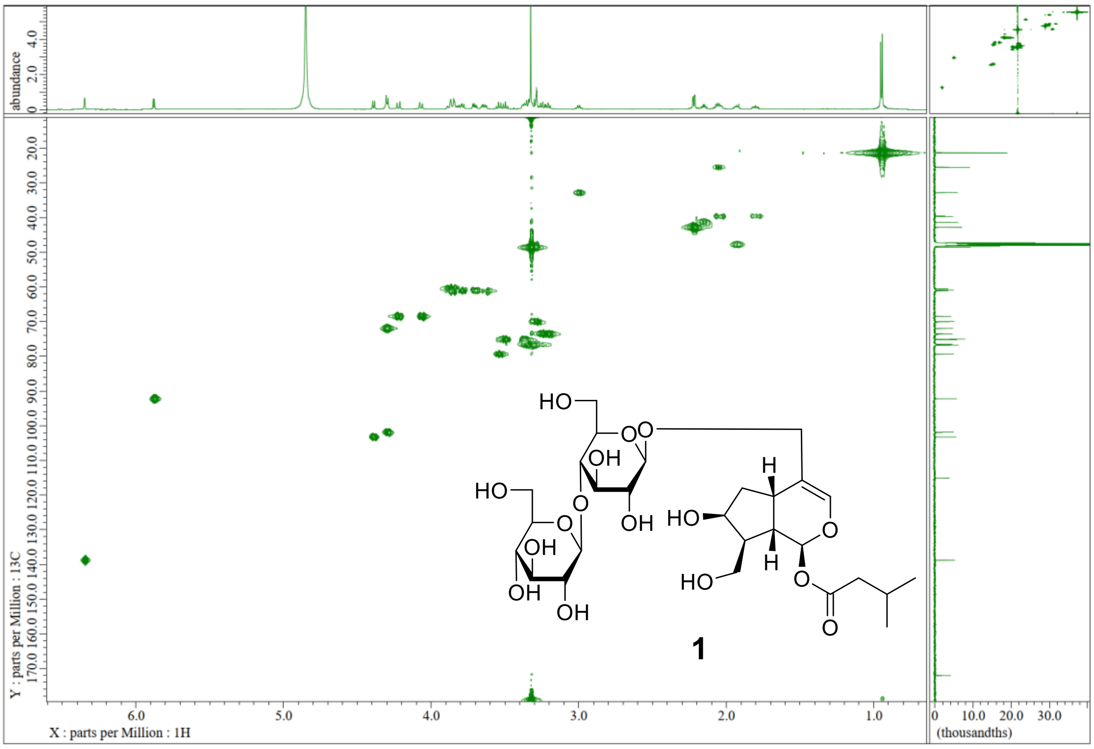

HMQC spectrum of **1**

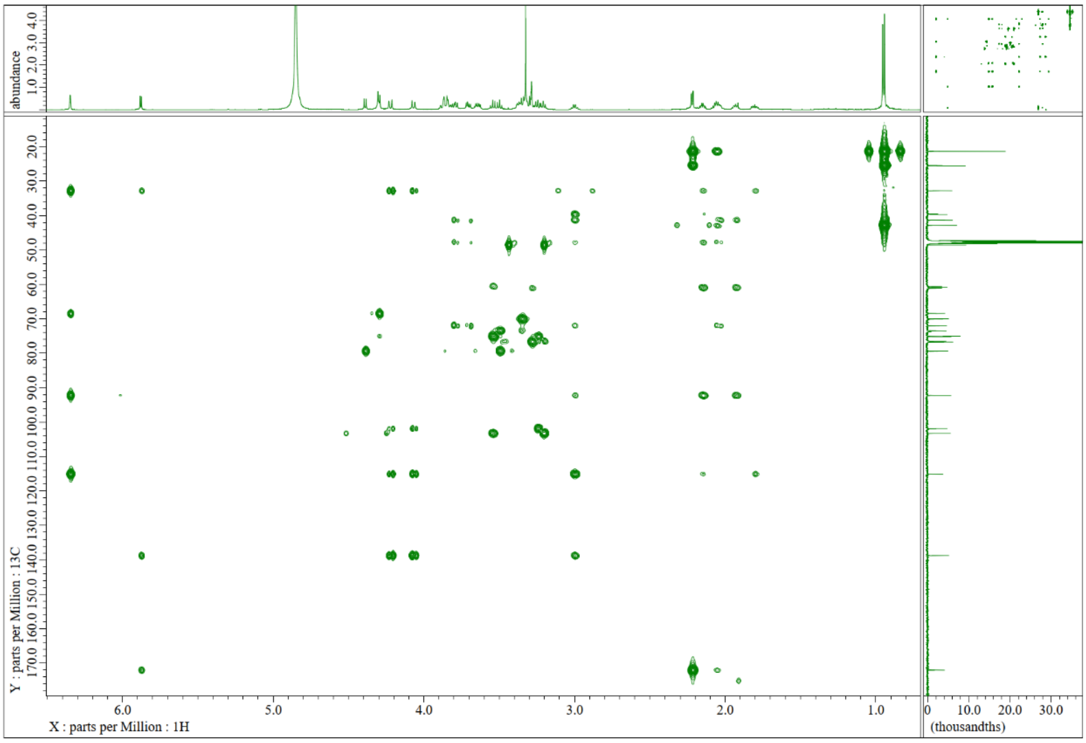

HMBC spectrum of **1**

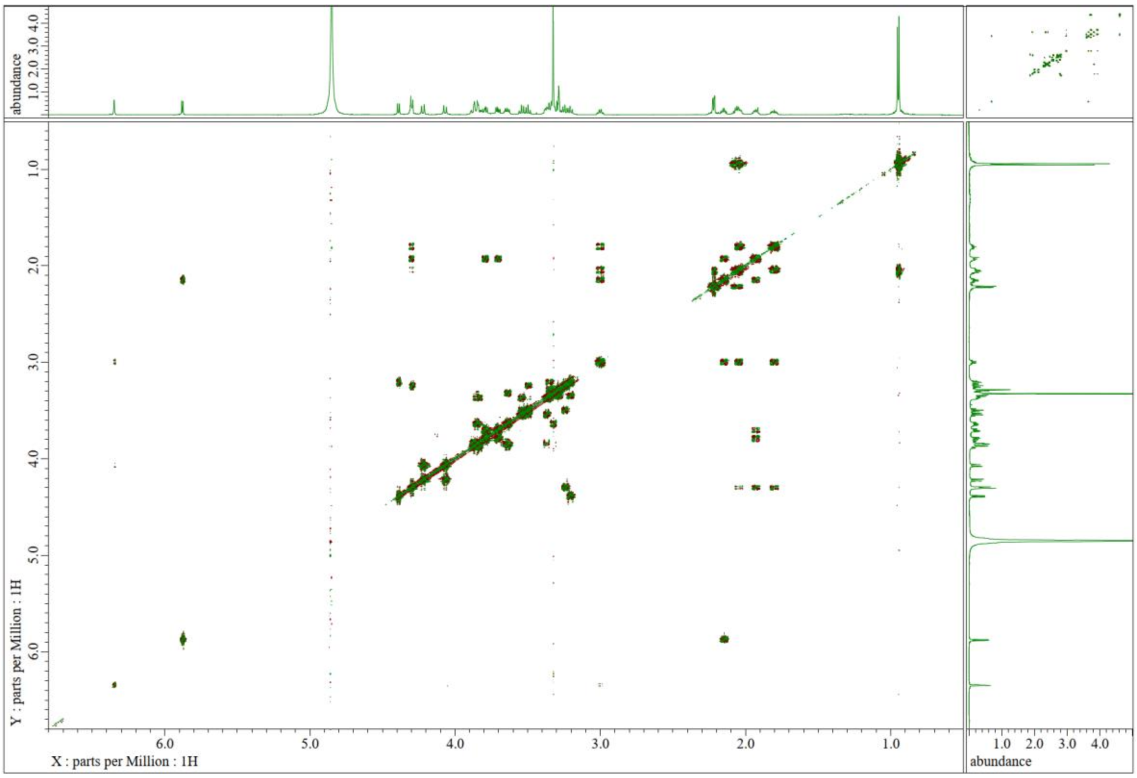

DQF spectrum of **1**

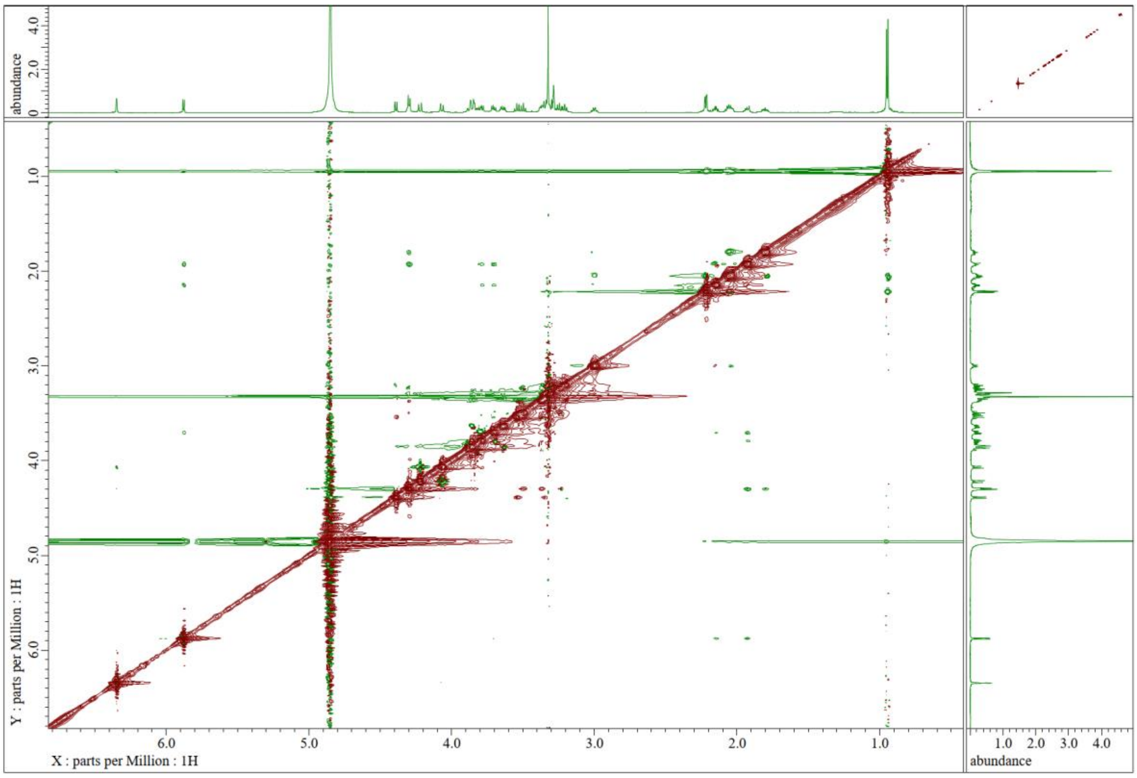

NOESY spectrum of **1**

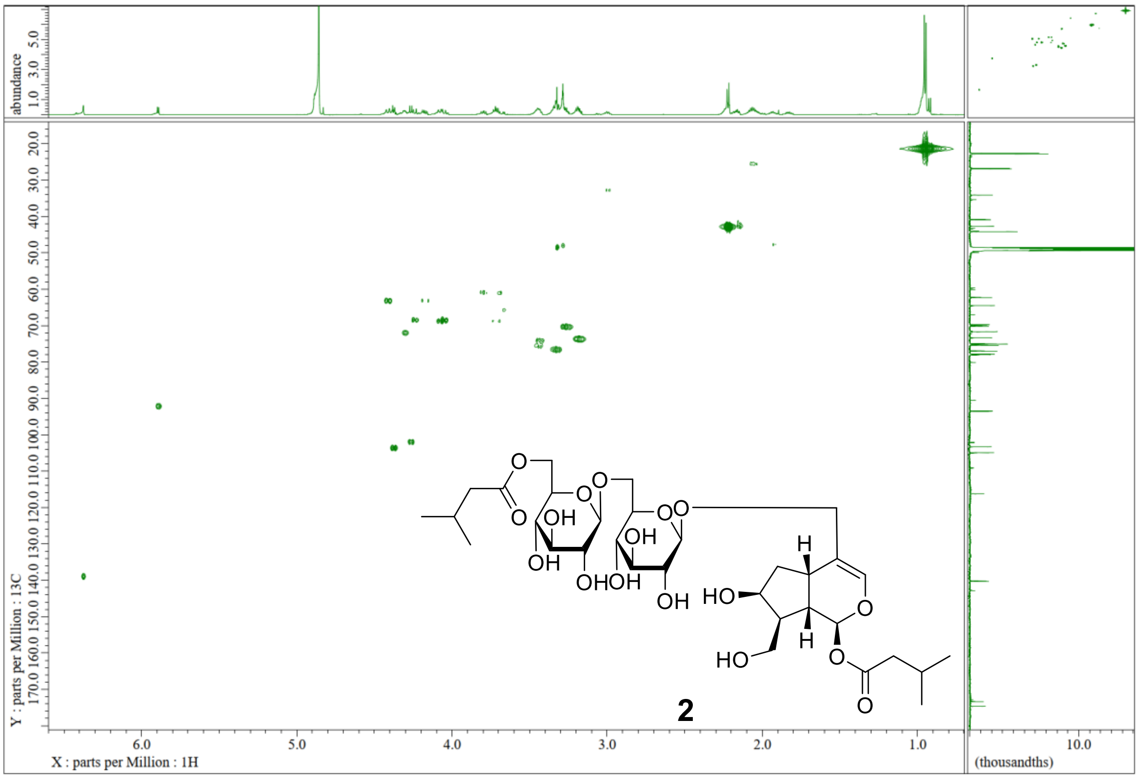

HMQC spectrum of **2**

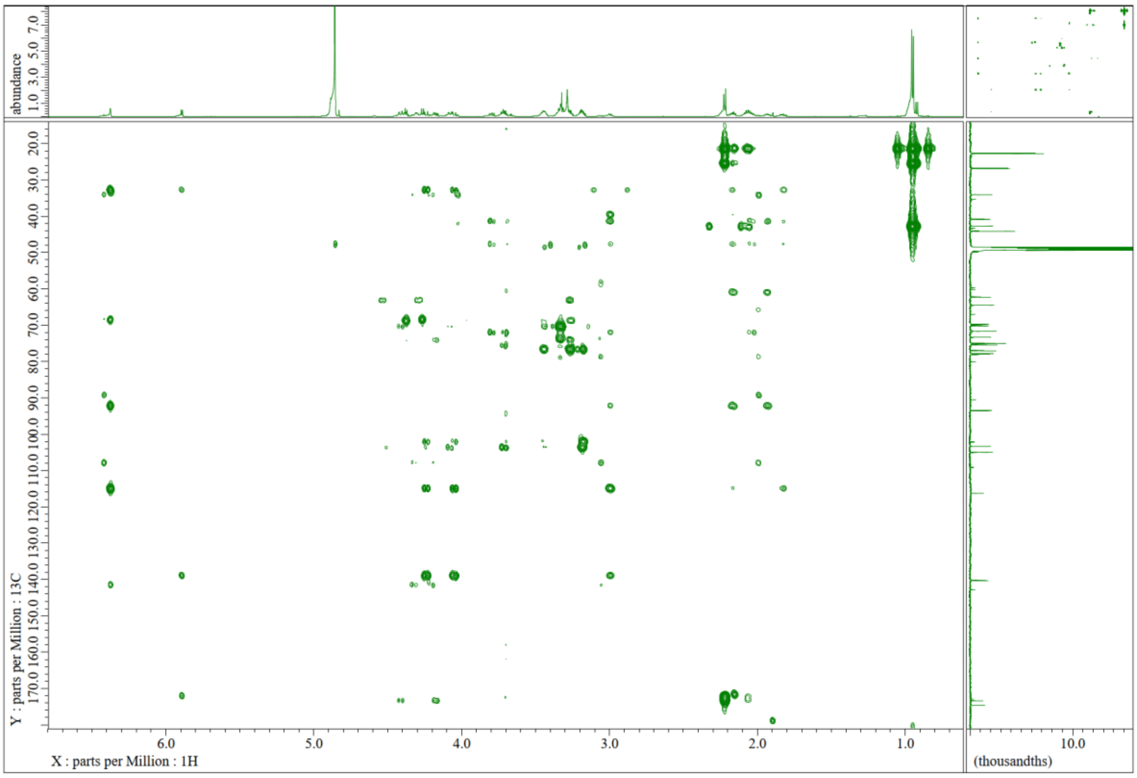

HMBC spectrum of **2**

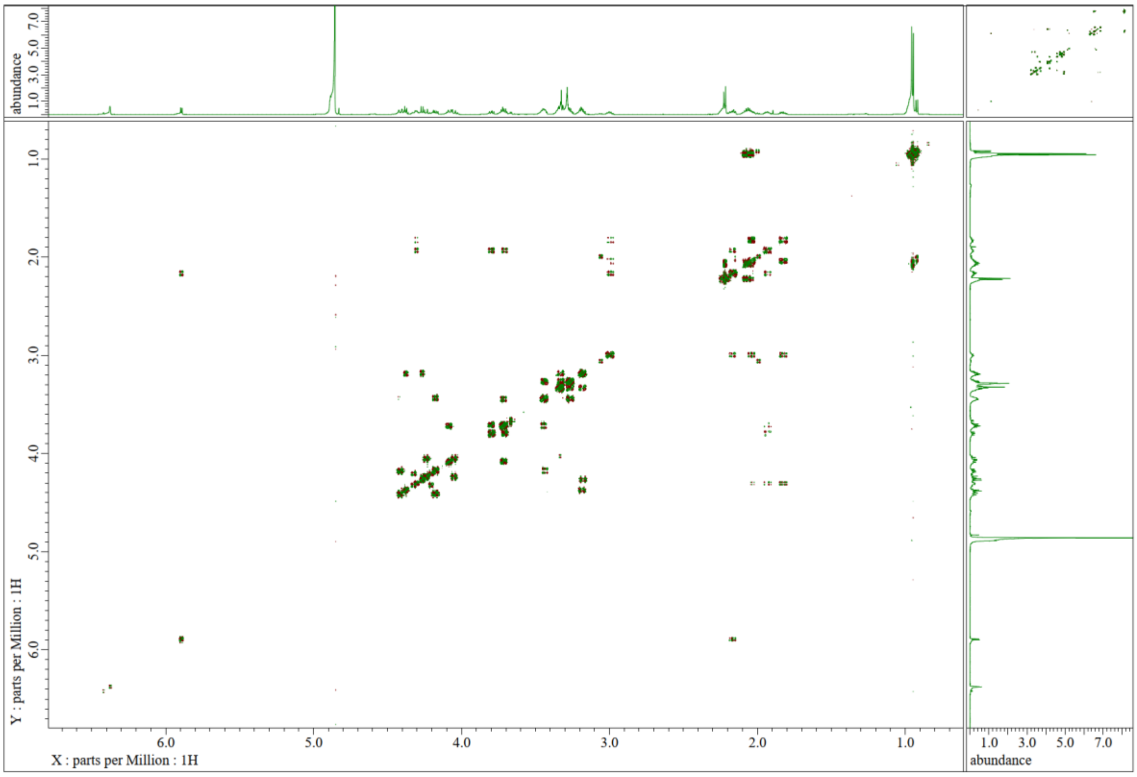

DQF spectrum of **2**

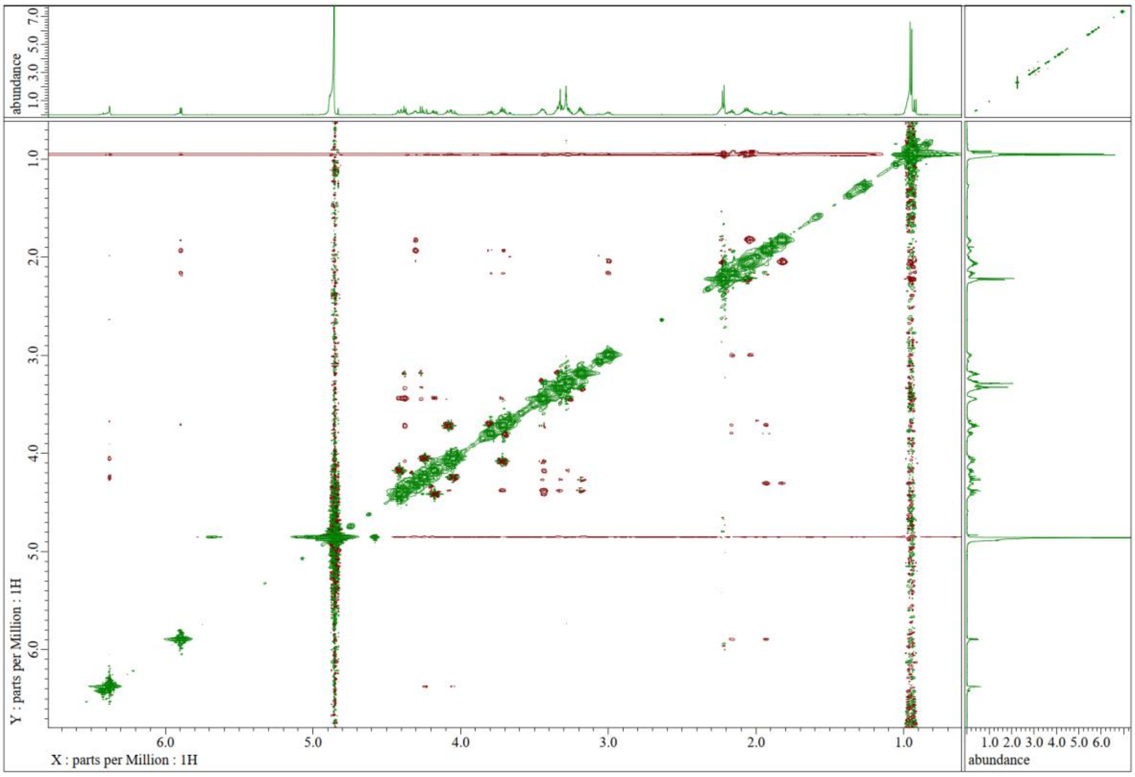

NOESY spectrum of **2**

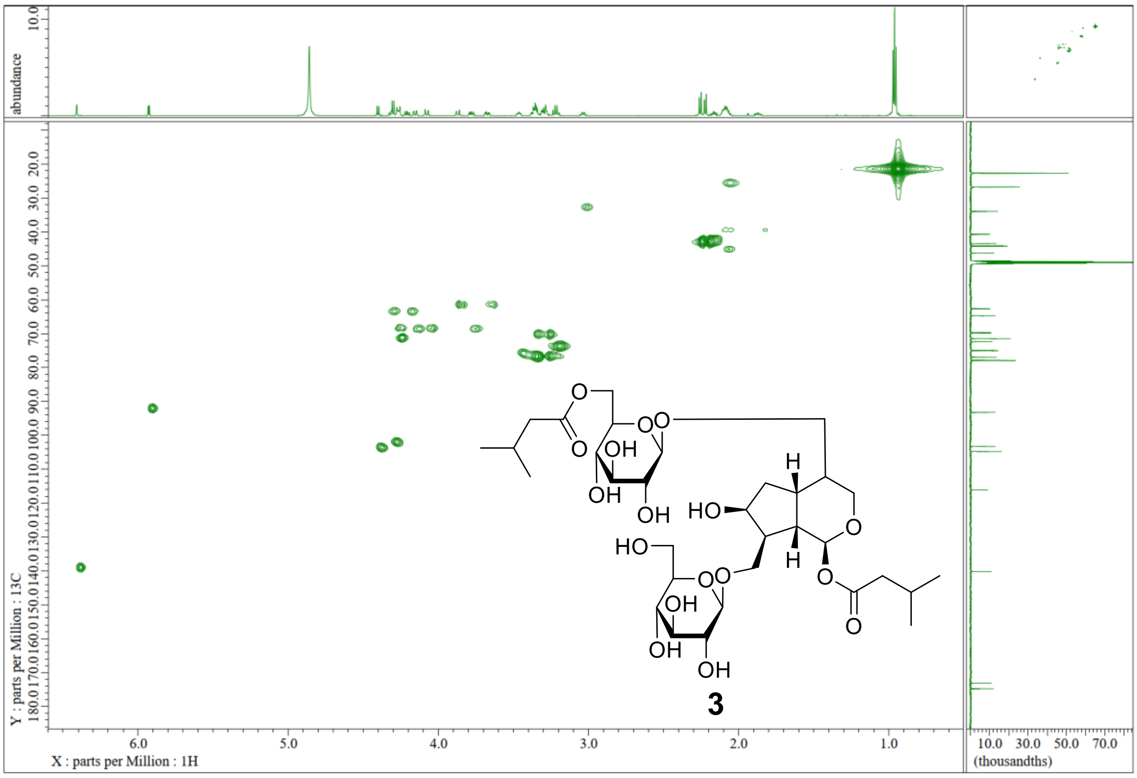

HMQC spectrum of **3**

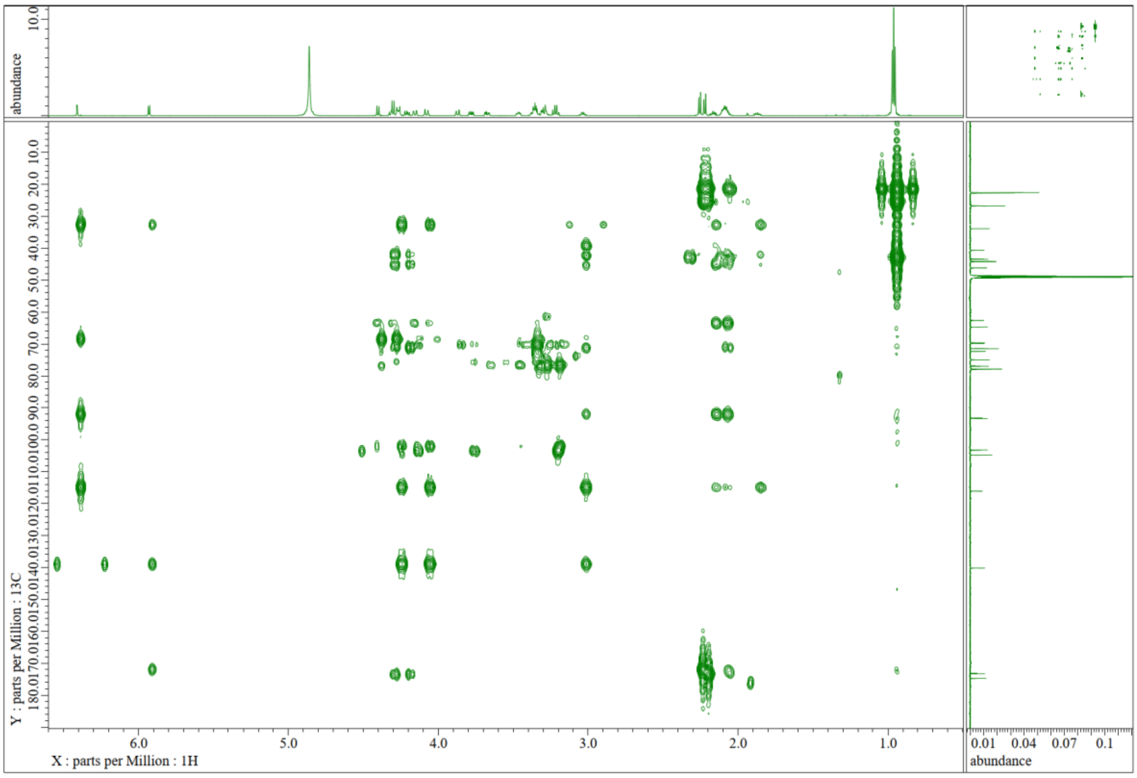

HMBC spectrum of **3**

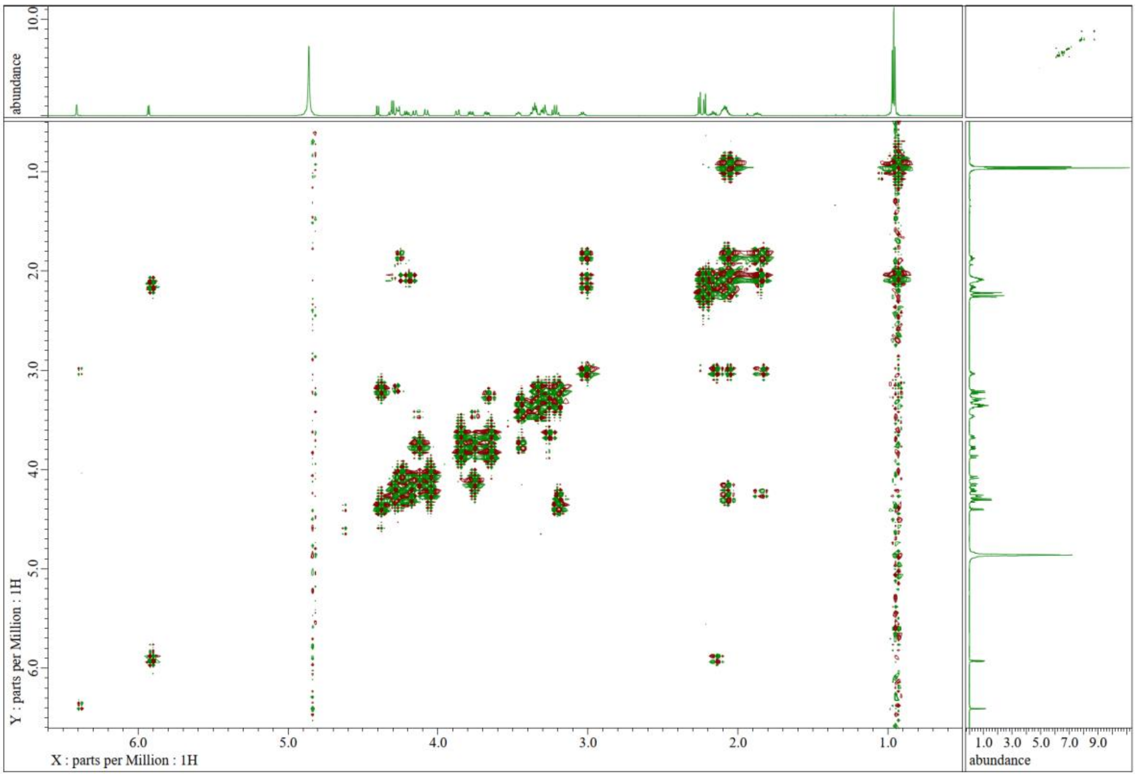

DQF spectrum of **3**

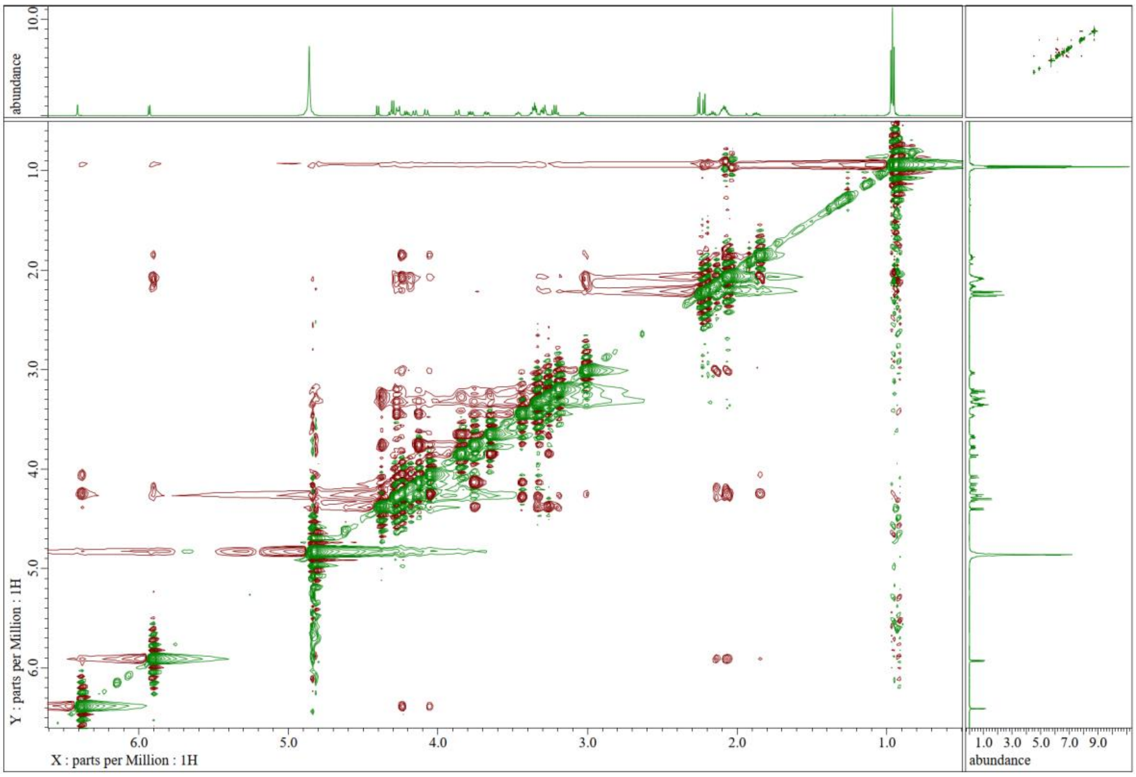

NOESY spectrum of **3**

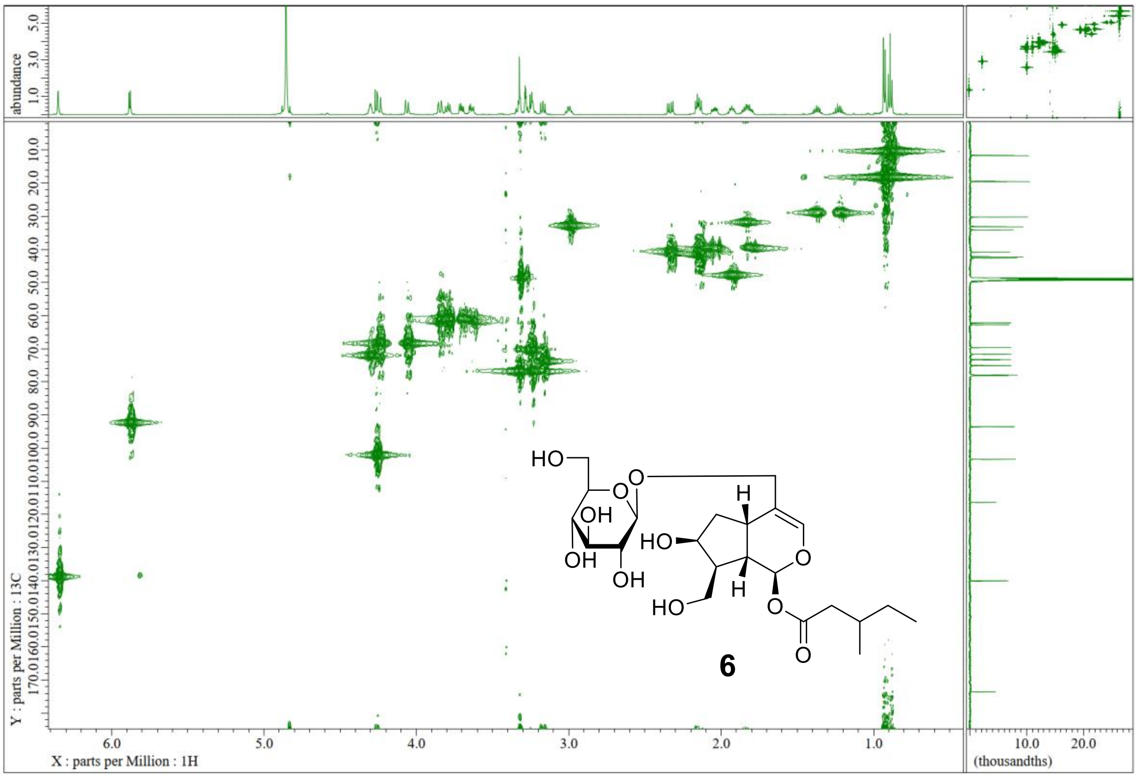

HMQC spectrum of **6**

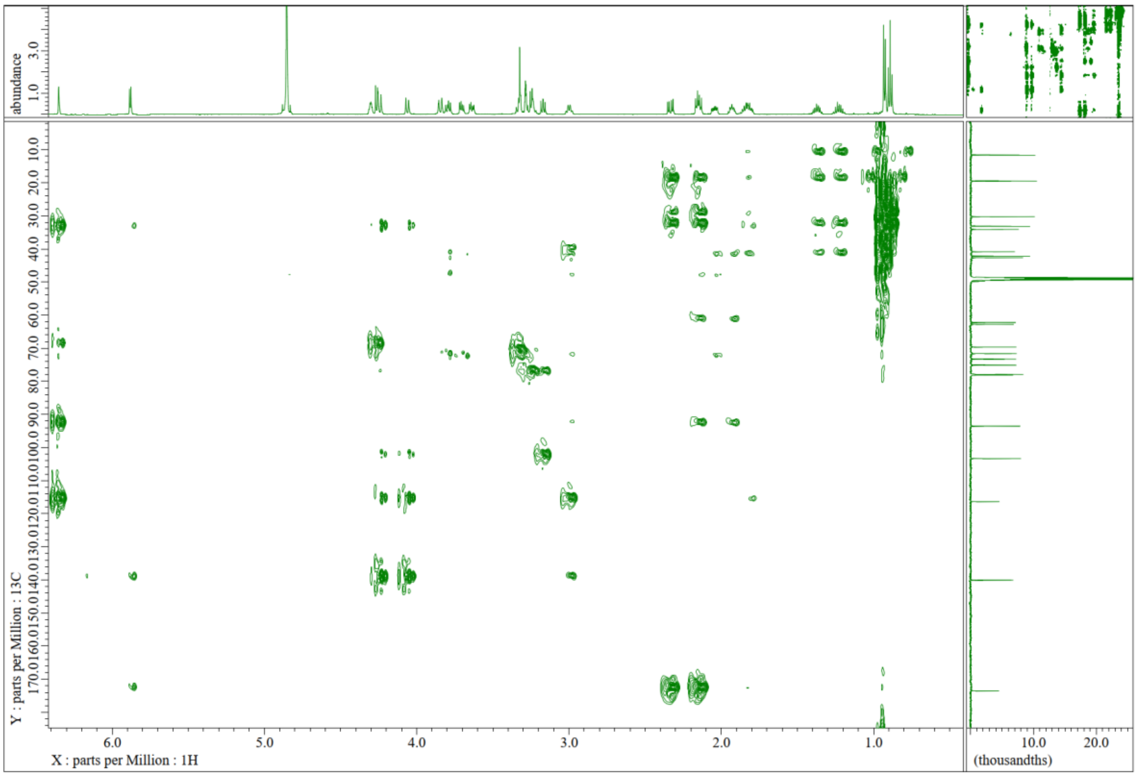

HMBC spectrum of **6**

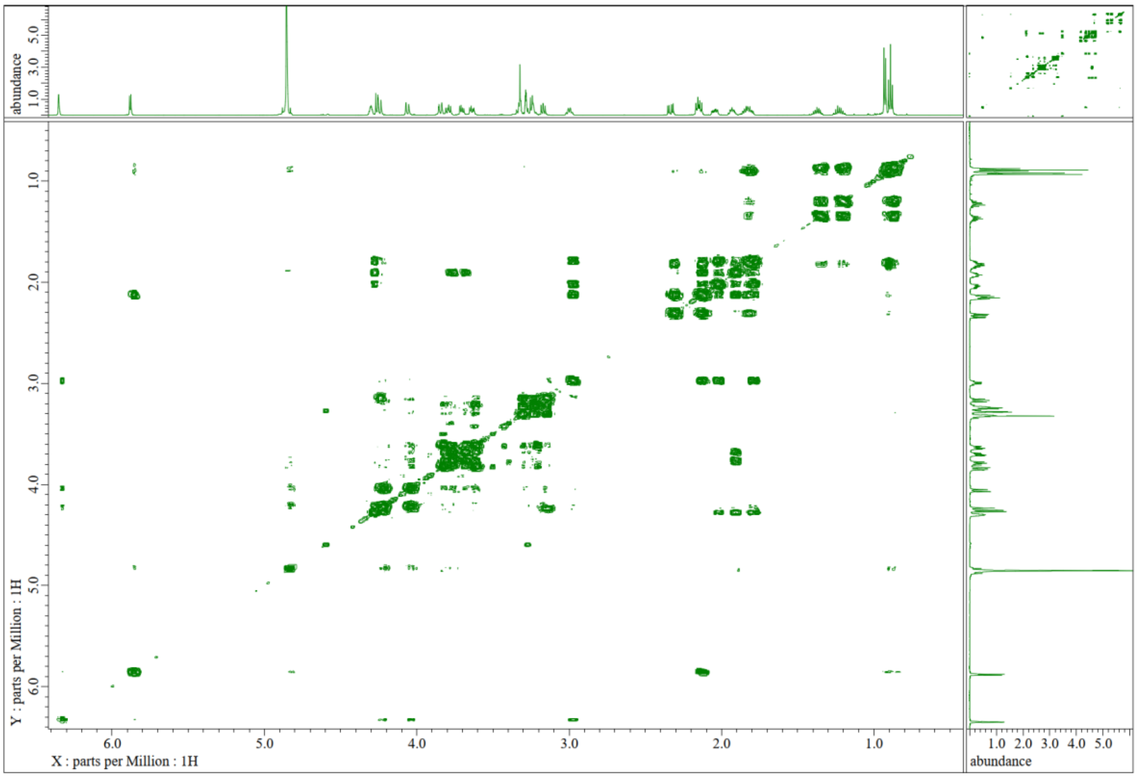

DQF spectrum of **6**

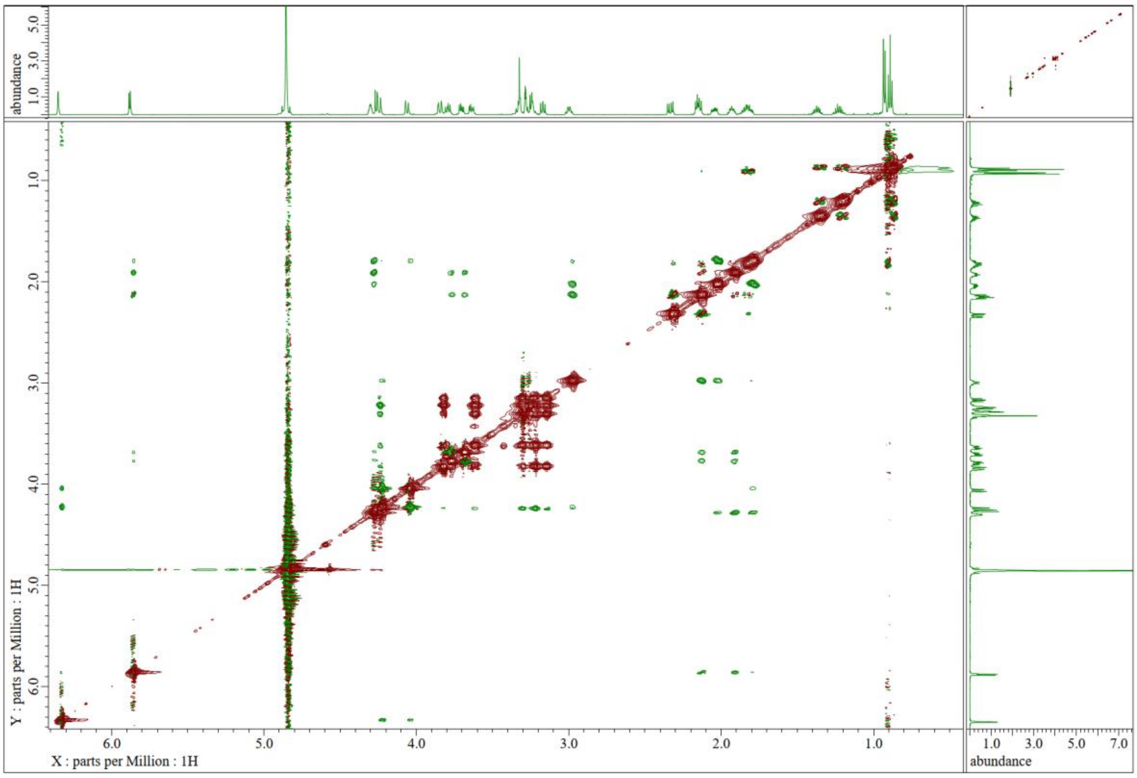

NOESY spectrum of **6**

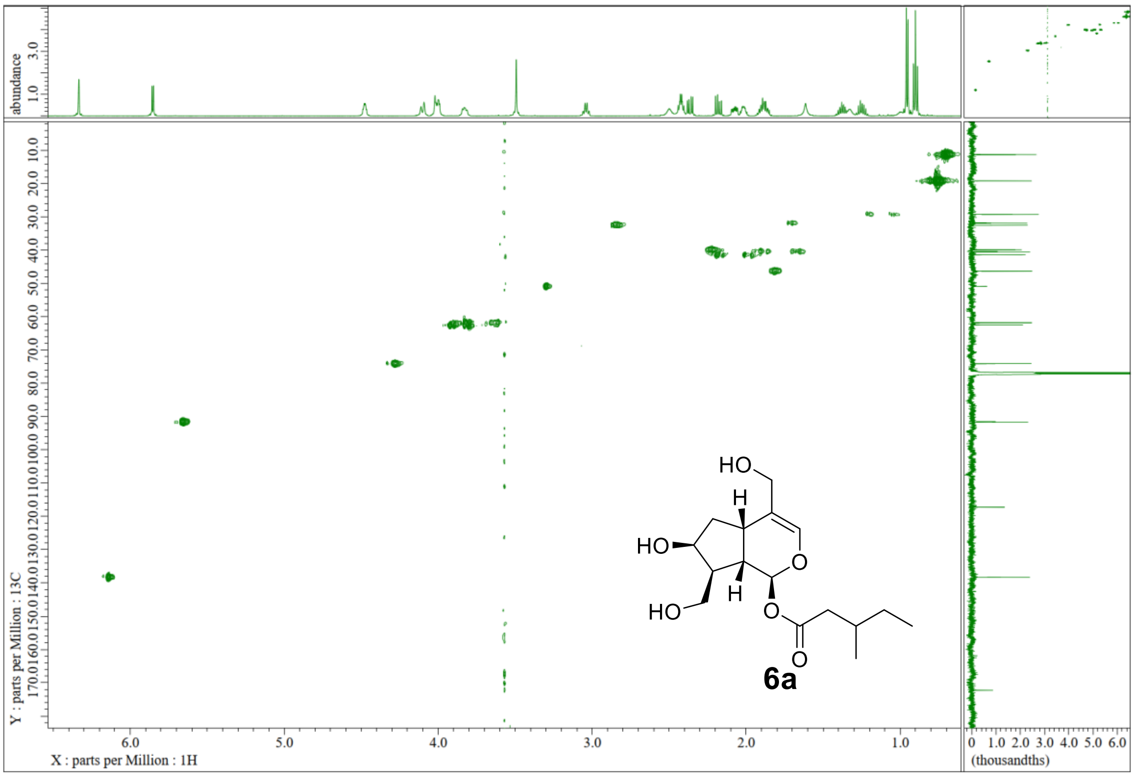

HMQC spectrum of **6a**

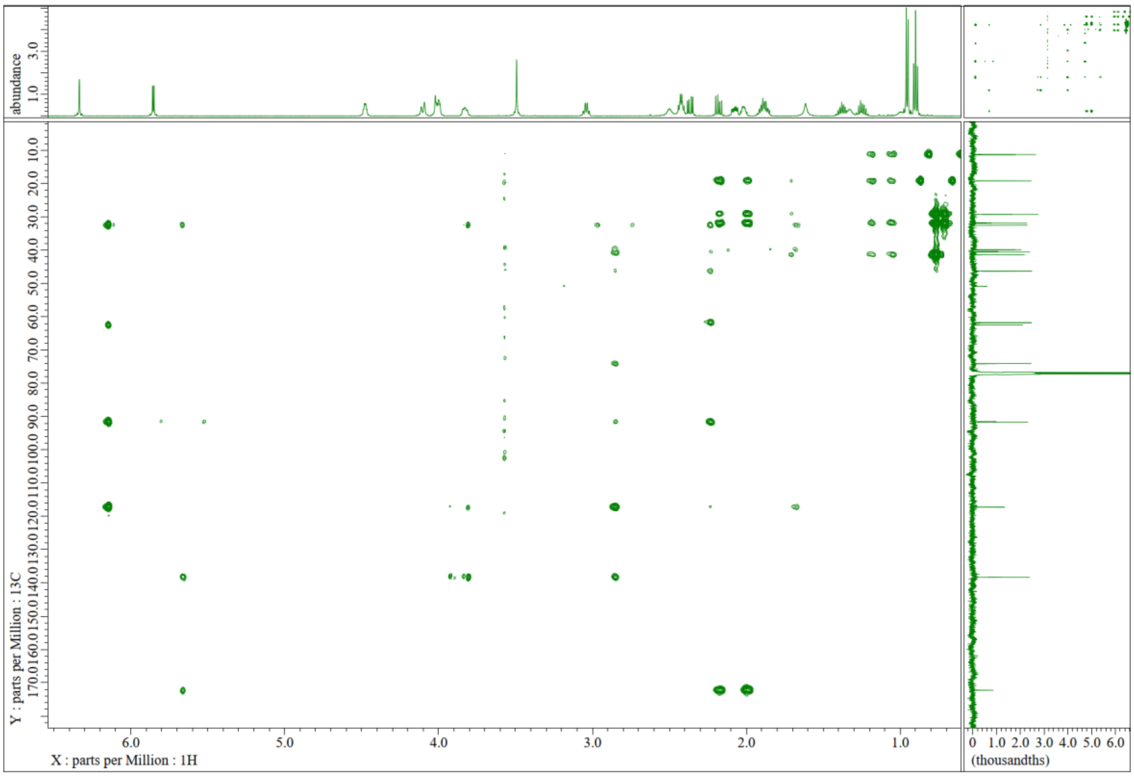

HMBC spectrum of **6a**

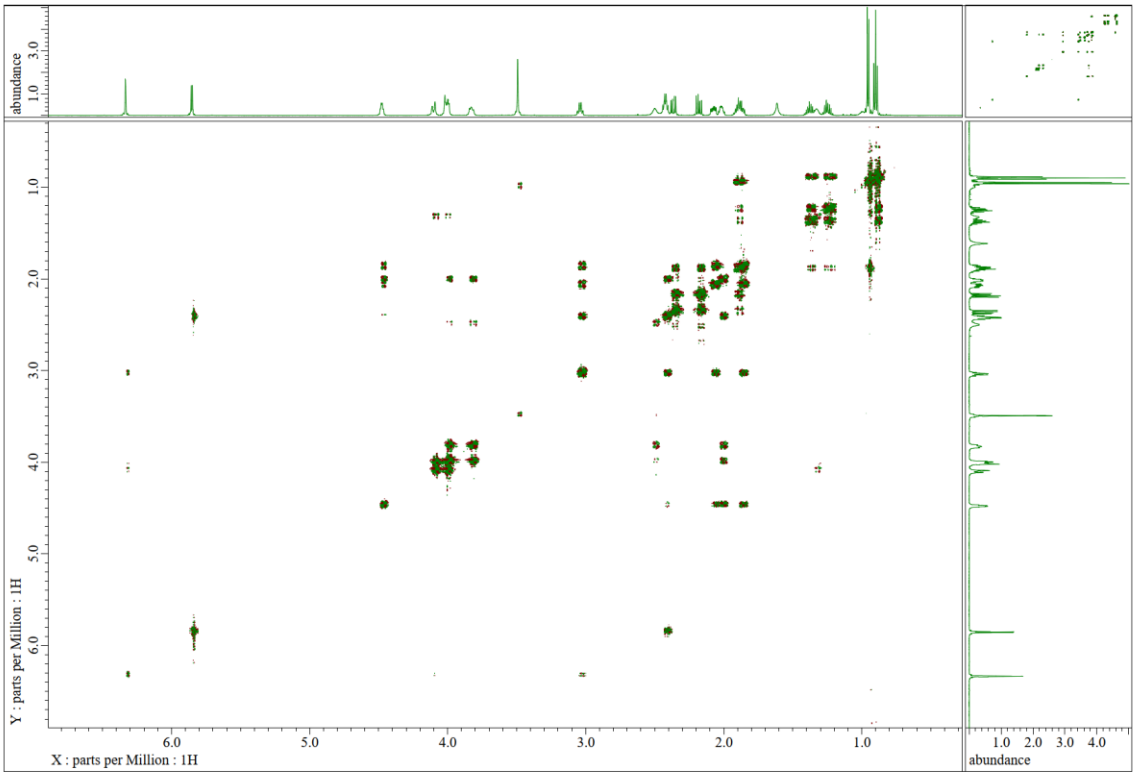

DQF spectrum of **6a**

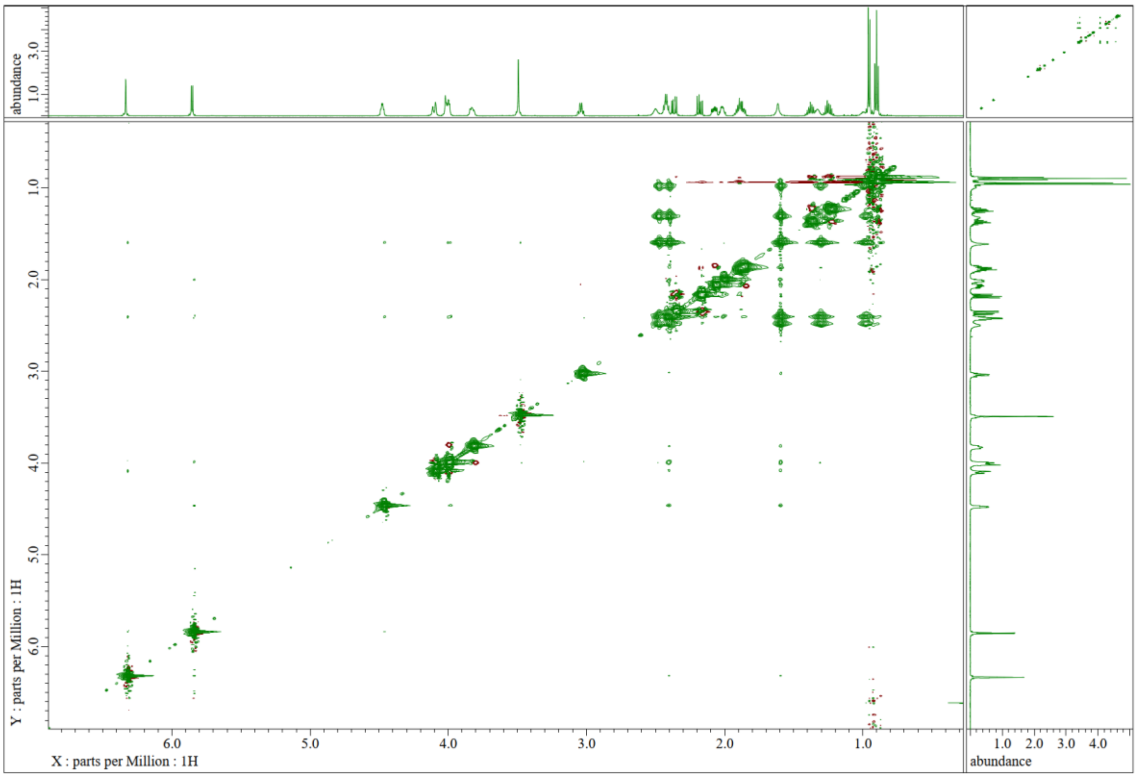

NOESY spectrum of **6a**

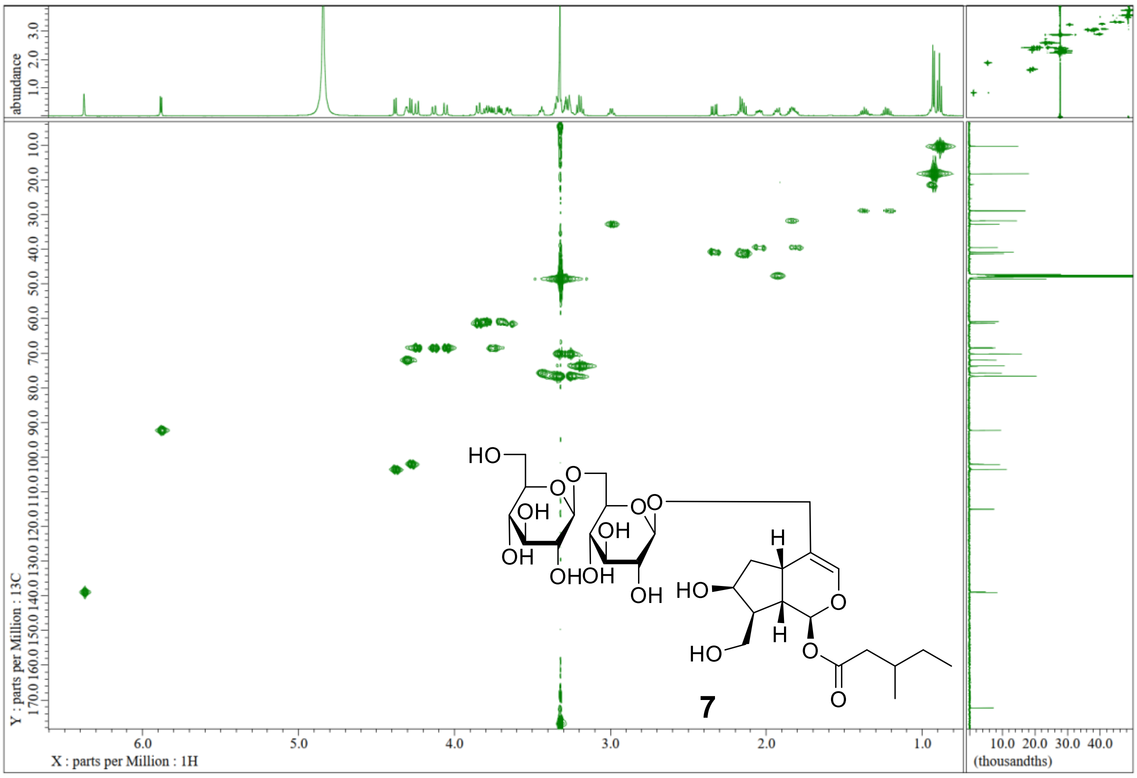

HMQC spectrum of **7**

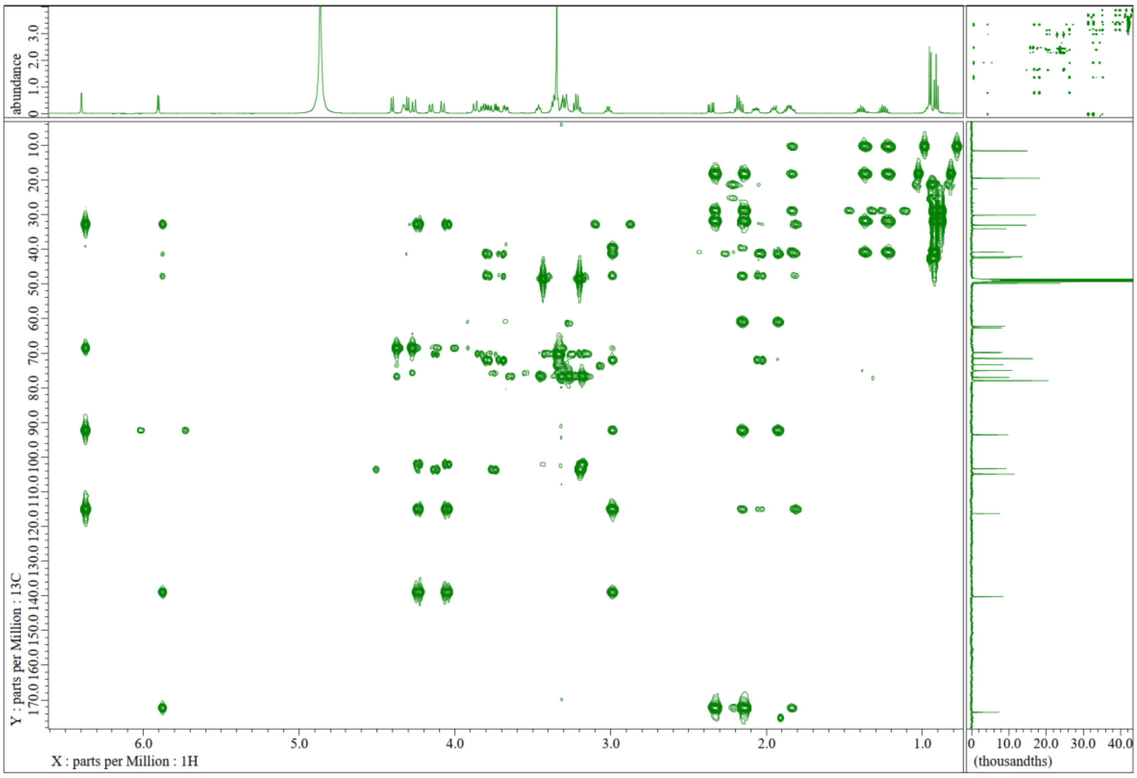

HMBC spectrum of **7**

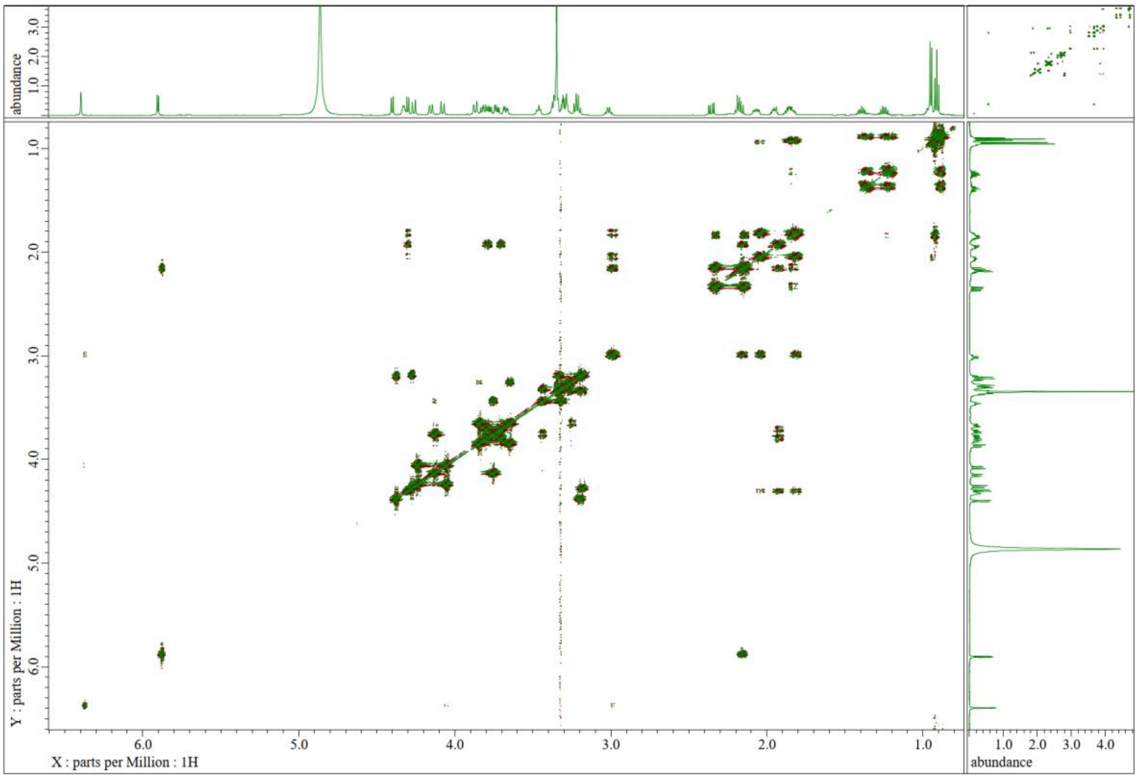

DQF spectrum of **7**

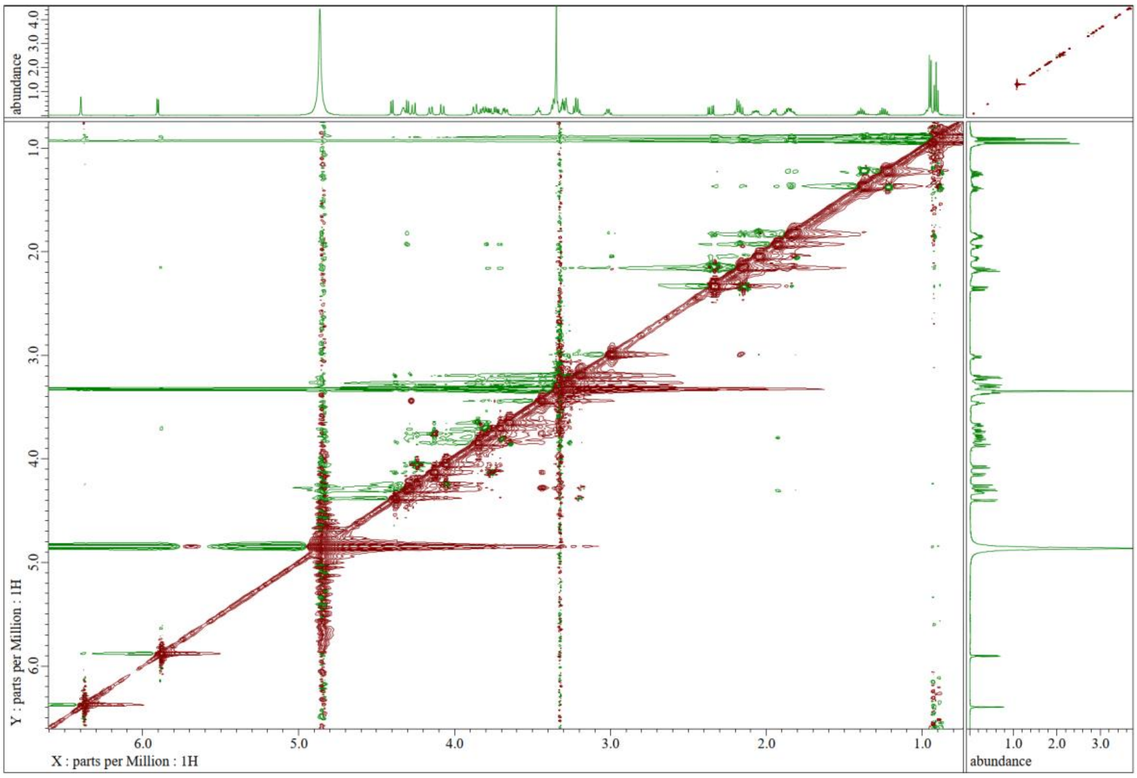

NOESY spectrum of **7**

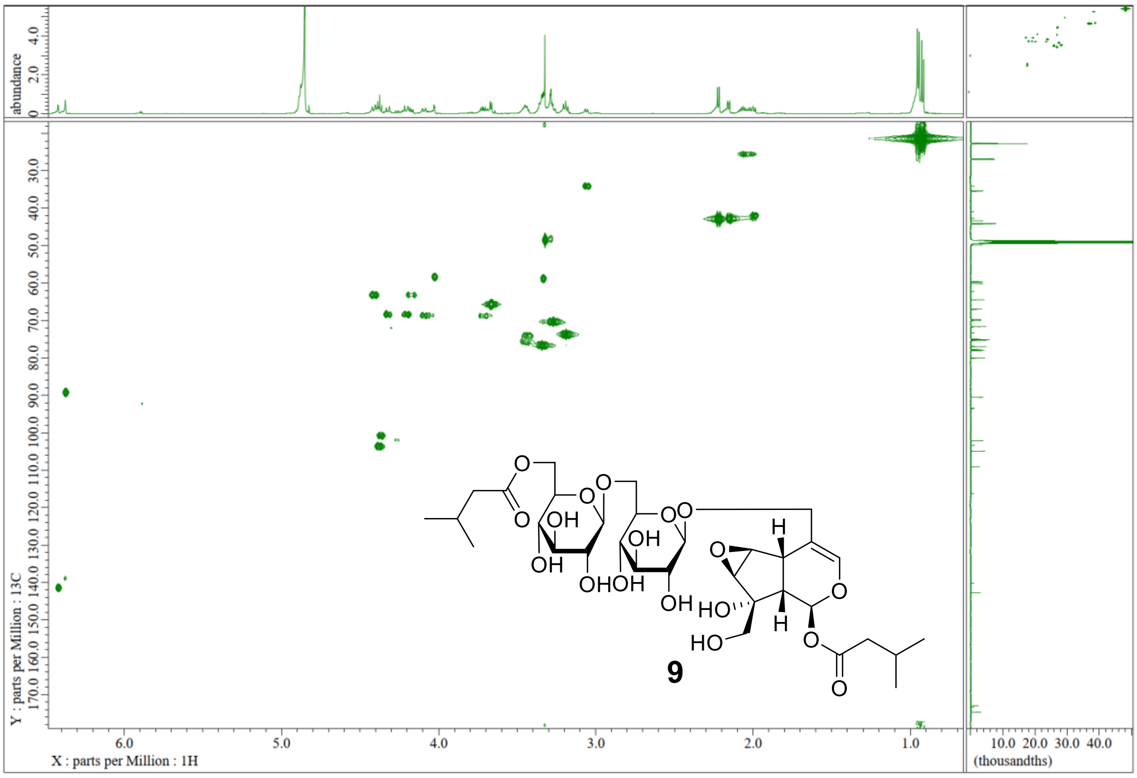

HMQC spectrum of **9**

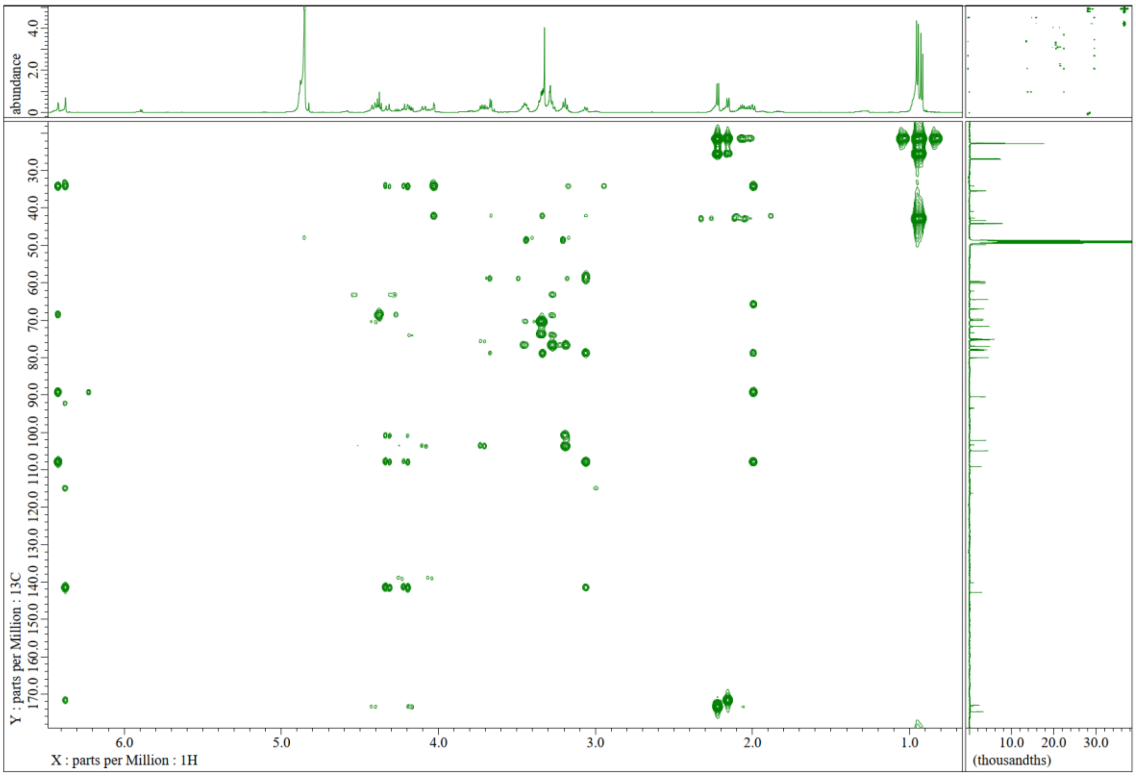

HMBC spectrum of **9**

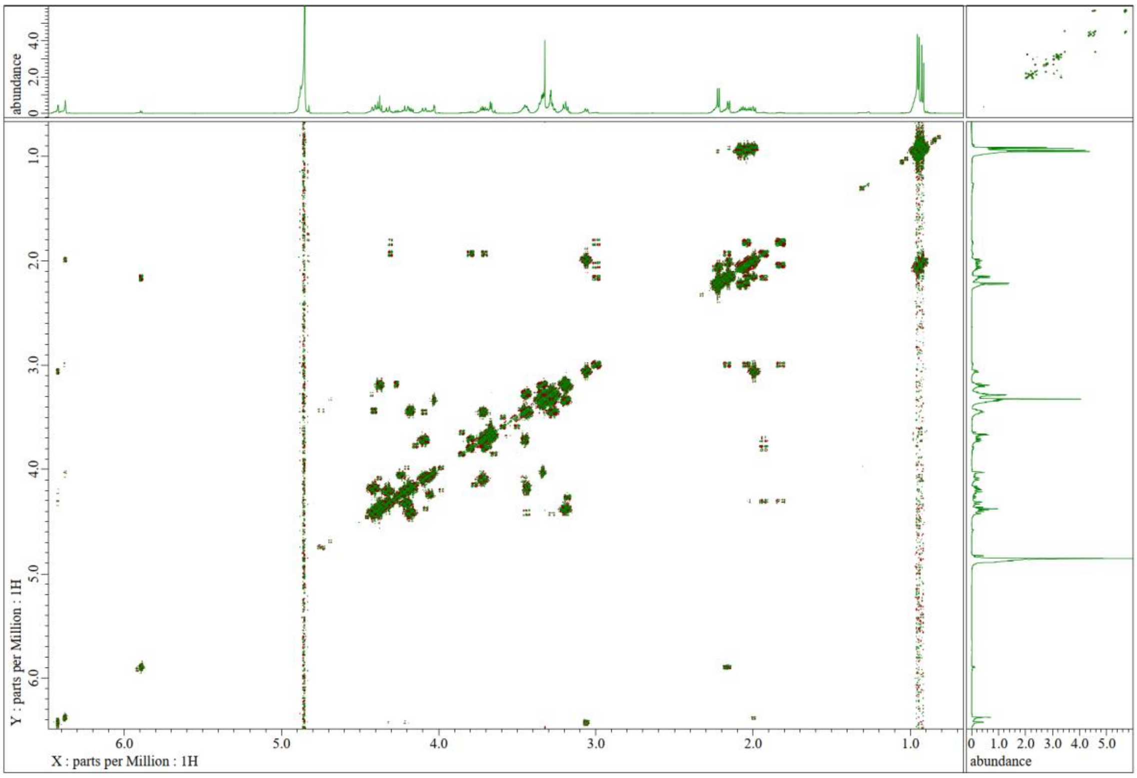

DQF spectrum of **9**

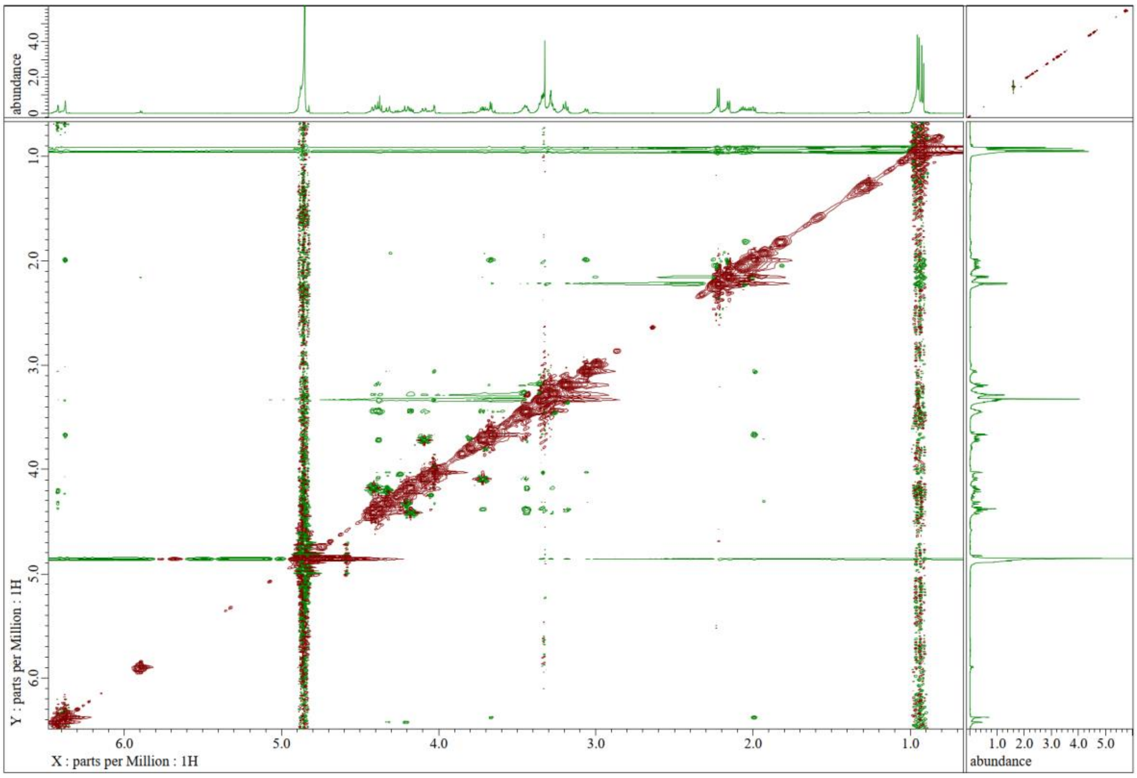

NOESY spectrum of **9**

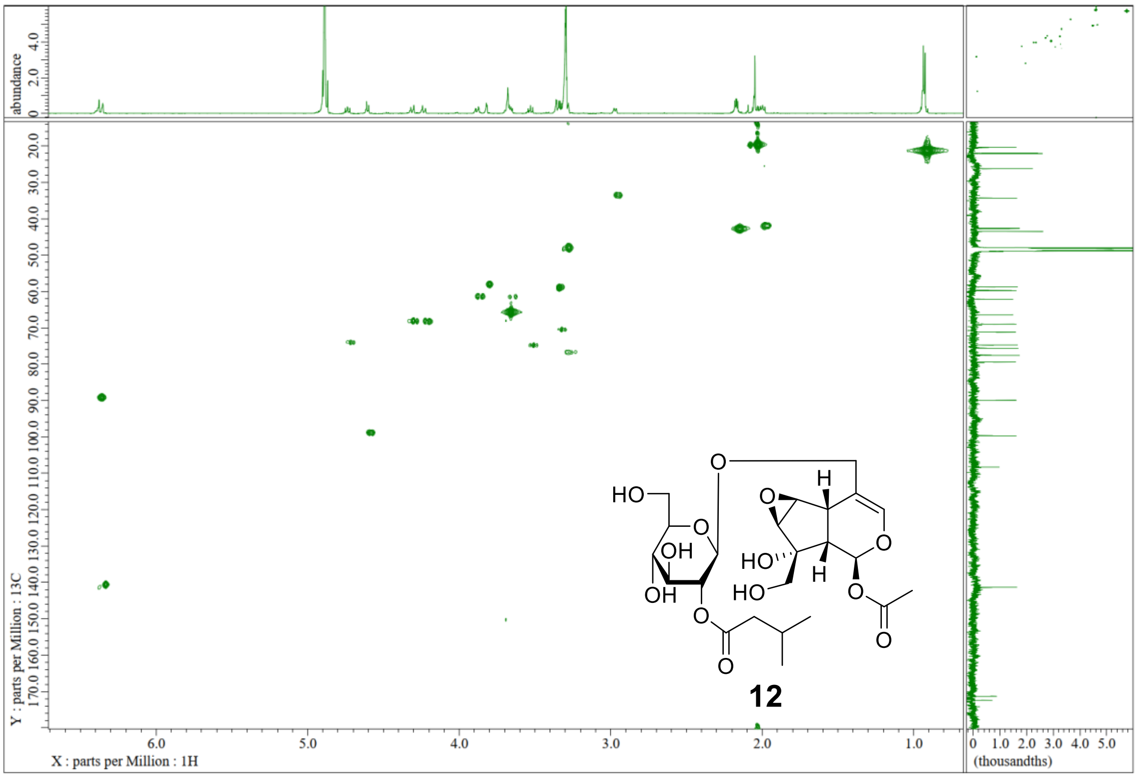

HMQC spectrum of **12**

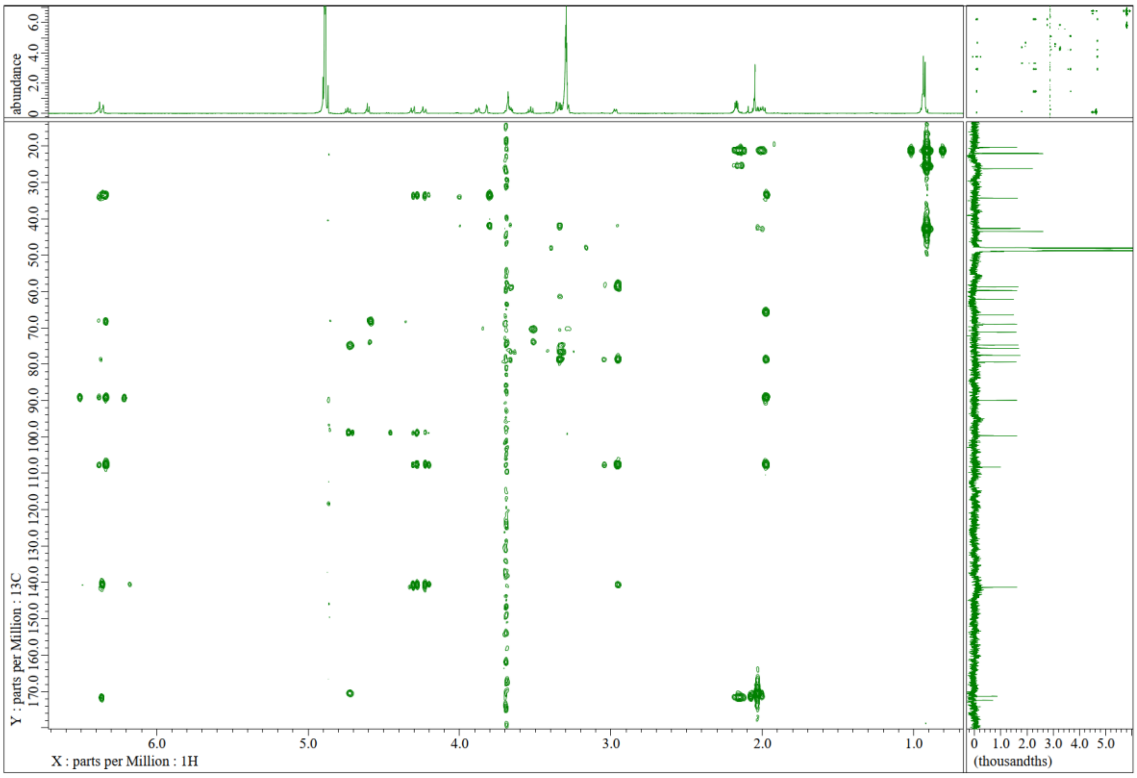

HMBC spectrum of **12**

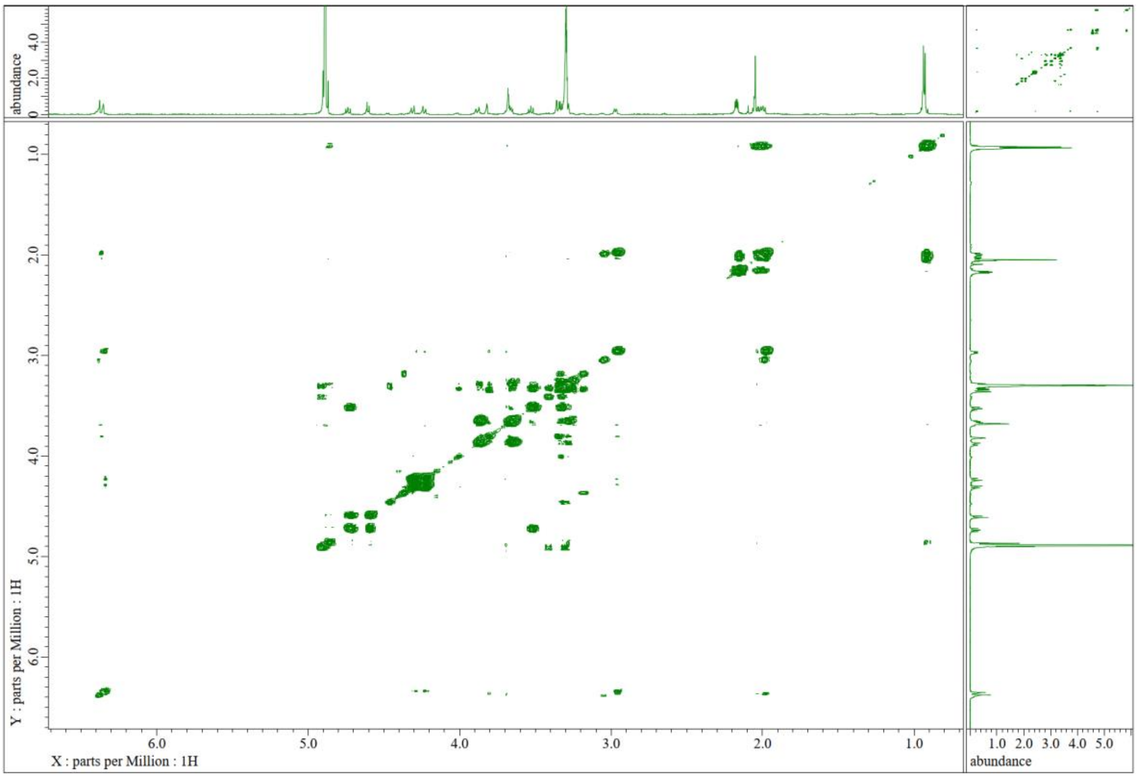

DQF spectrum of **12**

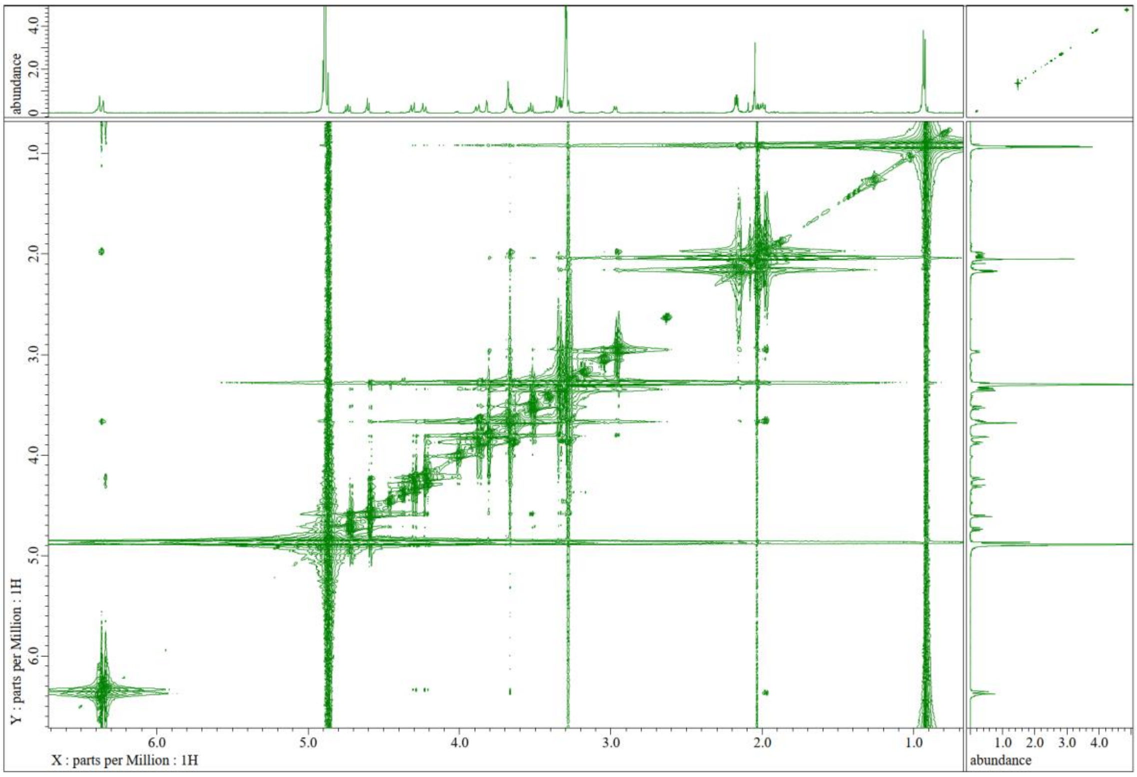

NOESY spectrum of **12**

S4. Optimized geometries, the minimum value of frequency, relative free-energies, and Boltzmann distributions of conformers of 1a, 6a, and 9a.

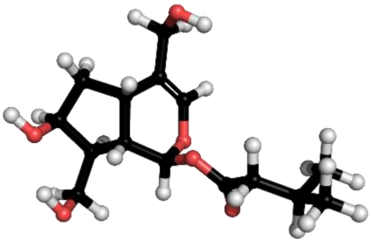

Conf. 1
[10.50]
ΔG = 0.00
P = 52.76

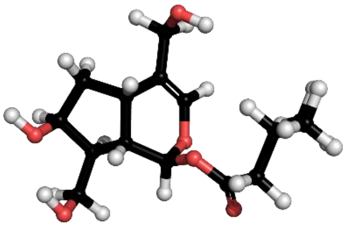

Conf. 2
[15.47]
ΔG = 0.64
P = 18.03

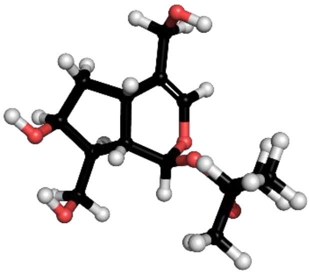

Conf. 3
[19.56]
ΔG = 0.89
P = 11.75

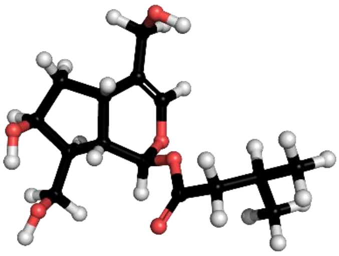

Conf. 4
[11.55]
ΔG = 1.41
P = 4.92

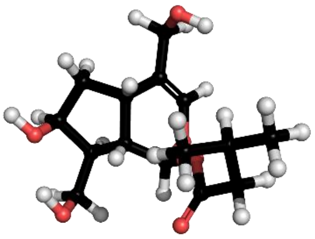

Conf. 5
[19.04]
ΔG = 1.45
P = 4.57

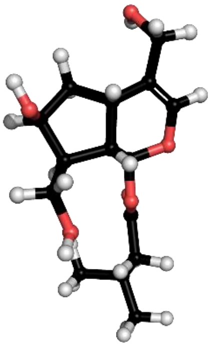

Conf. 6
[15.95]
ΔG = 1.55
P = 3.85

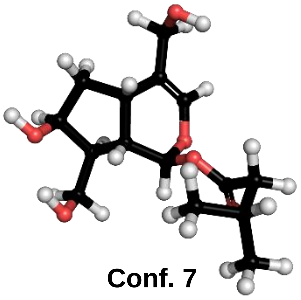

Conf. 7
[17.97]
ΔG = 2.22
P = 1.24

Figure S4-1 The optimized structures of 7 conformers of **1a** with the minimum value of frequency [in brackets, cm^{-1}], relative free-energy (Δ*G*, kcal/mol), and Boltzmann distribution (P, %), at 298.15 K, calculated at the CAM-B3LYP/def2-TZVP level in MeOH.

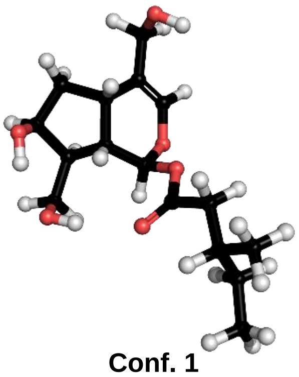

Conf. 1
[8.62]
ΔG = 0.00
P = 36.82

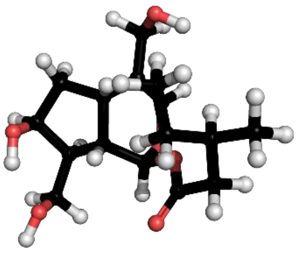

Conf. 2
[12.74]
ΔG = 0.10
P = 30.81

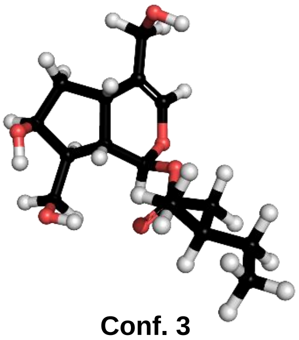

Conf. 3
[19.56]
ΔG = 0.77
P = 10.06

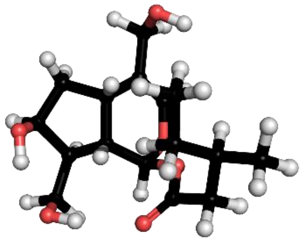

Conf. 4
[16.04]
ΔG = 0.82
P = 9.22

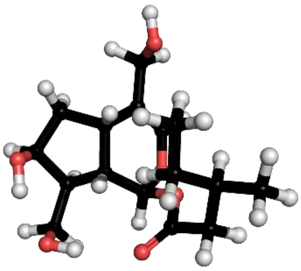

Conf. 5
[18.47]
ΔG = 1.26
P = 4.37

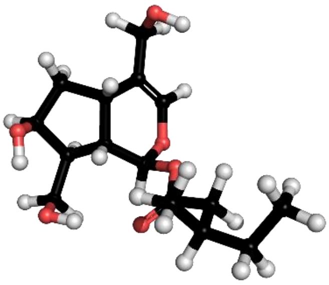

Conf. 6
[16.94]
ΔG = 1.34
P = 3.84

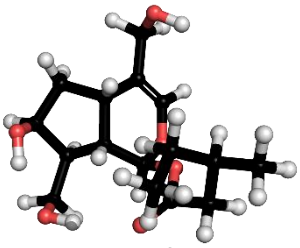

Conf. 7
[19.24]
ΔG = 1.91
P = 1.47

Figure S4-2 The optimized structures of 7 conformers of **6a** with the minimum value of frequency [in brackets, cm^{-1}], relative free-energy (ΔG, kcal/mol), and Boltzmann distribution (P, %), at 298.15 K, calculated at the CAM-B3LYP/def2-TZVP level in MeOH.

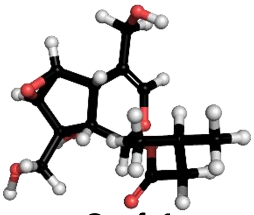

Conf. 1
[18.42]
ΔG = 0.00
P = 18.82

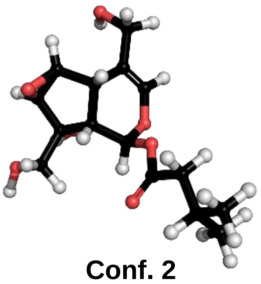

Conf. 2
[24.91]
ΔG = 0.14
P = 14.78

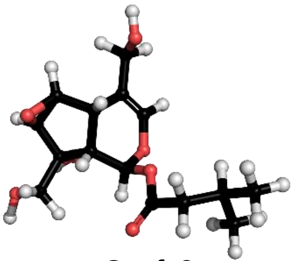

Conf. 3
[12.55]
ΔG = 0.16
P = 14.26

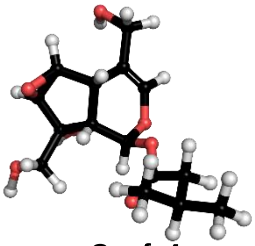

Conf. 4
[18.09]
ΔG = 0.23
P = 12.66

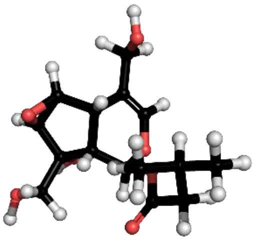

Conf. 5
[15.49]
ΔG = 0.25
P = 12.33

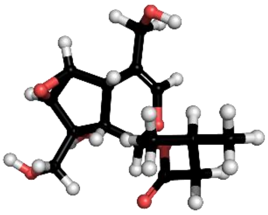

Conf. 6
[20.54]
ΔG = 0.55
P = 7.49

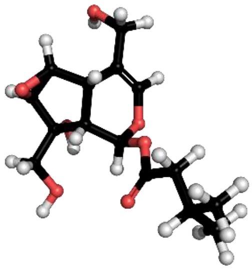

Conf. 7
[18.13]
ΔG = 1.01
P = 3.43

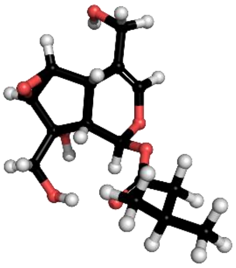

Conf. 8
[19.47]
ΔG = 1.01
P = 3.41

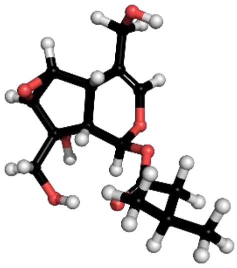

Conf. 9
[20.04]
ΔG = 1.13
P = 2.77

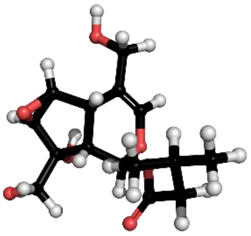

Conf. 10
[21.36]
ΔG = 1.14
P = 2.73

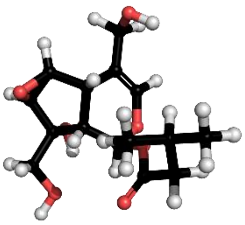

Conf. 11
[11.54]
ΔG = 1.31
P = 2.05

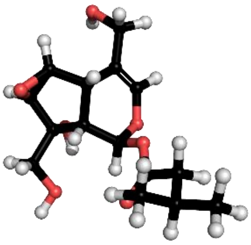

Conf. 12
[17.81]
ΔG = 1.34
P = 1.97

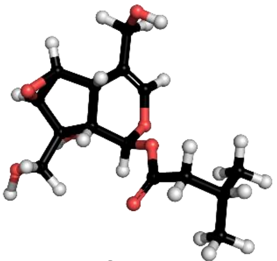

Conf. 13
[19.08]
ΔG = 1.52
P = 1.44

Figure S4-3 The optimized structures of 13 conformers of **9a** with the minimum value of frequency [in brackets, cm^{-1}], relative free-energy (ΔG, kcal/mol), and Boltzmann distribution (P, %), at 298.15 K, calculated at the CAM-B3LYP/def2-TZVP level in MeOH.

S5. Anti-proliferative activity of 1a, 6a, and 9a against non-CSCs and CSCs from MDA-MB-231 cells.

Table S5-1 Inhibition (%) of cell proliferation of **1a**, **6a**, and **9a** against non-CSCs (MDA-MB-231 calls). ADR (positive control) inhibited 27.9±6.7% at 0.05 μM. Inhibition ratio (%) of relative cell proliferation are shown as means ±SD of three replicates. Statical significance was analyzed using the Dunnett's test (**P* < 0.05, ***P* < 0.01).

Compounds	Concentrations of test compounds (μM)							IC$_{50}$ (μM)
	Control	20 μM	40 μM	60 μM	80 μM	100 μM	120 μM	
**1a**	0±5.7	-	-	16.0±4.3*	38.8±10.8**	48.6±6.8**	72.3±1.7**	> 100
**6a**	0±6.0	4.2±4.8	38.5±3.3**	64.3±3.6**	79.2±3.6**	77.7±3.0**		47.7±4.2
**9a**	0±5.7	-	-	8.7±11.4	46.5±6.8**	30.3±3.6**	70.9±5.3**	> 100

Table S5-2 Inhibition (%) of cell proliferation of **1a**, **6a**, and **9a** against CSCs from MDA-MB-231 cells. ADR (positive control) inhibited 50.3±2.4% at 0.05 μM. Inhibition ratio (%) of relative cell proliferation are shown as means ±SD of three replicates. Statical significance was analyzed using the Dunnett's test (**P* < 0.05, ***P* < 0.01).

Compounds	Concentrations of test compounds (μM)								IC$_{50}$ (μM)
	Control	10 μM	20 μM	40 μM	60 μM	80 μM	100 μM	120 μM	
**1a**	0±5.9	-	-	37.4±6.9**	70.5±2.1**	93.3±2.2**	94.3±0.1**	97.4±0.4**	45.0±5.3
**6a**	0±1.2	16.2±3.6**	60.4±1.9**	84.3±1.2**	96.5±0.4**	98.6±0.2**	98.1±4.6**		17.6±1.0
**9a**	0±5.9	-	-	38.1±6.1**	55.8±1.0**	91.8±2.5**	98.5±0.3**	98.5±0.3**	47.7±3.9
